# Supplementary figures and images for: DNAJ heat shock protein family member C1 can regulate proliferation and migration in hepatocellular carcinoma
Source: PeerJ. 2023 Jul 26;11:e15700. doi: 10.7717/peerj.15700 (PMC10386825; doi:10.7717/peerj.15700)

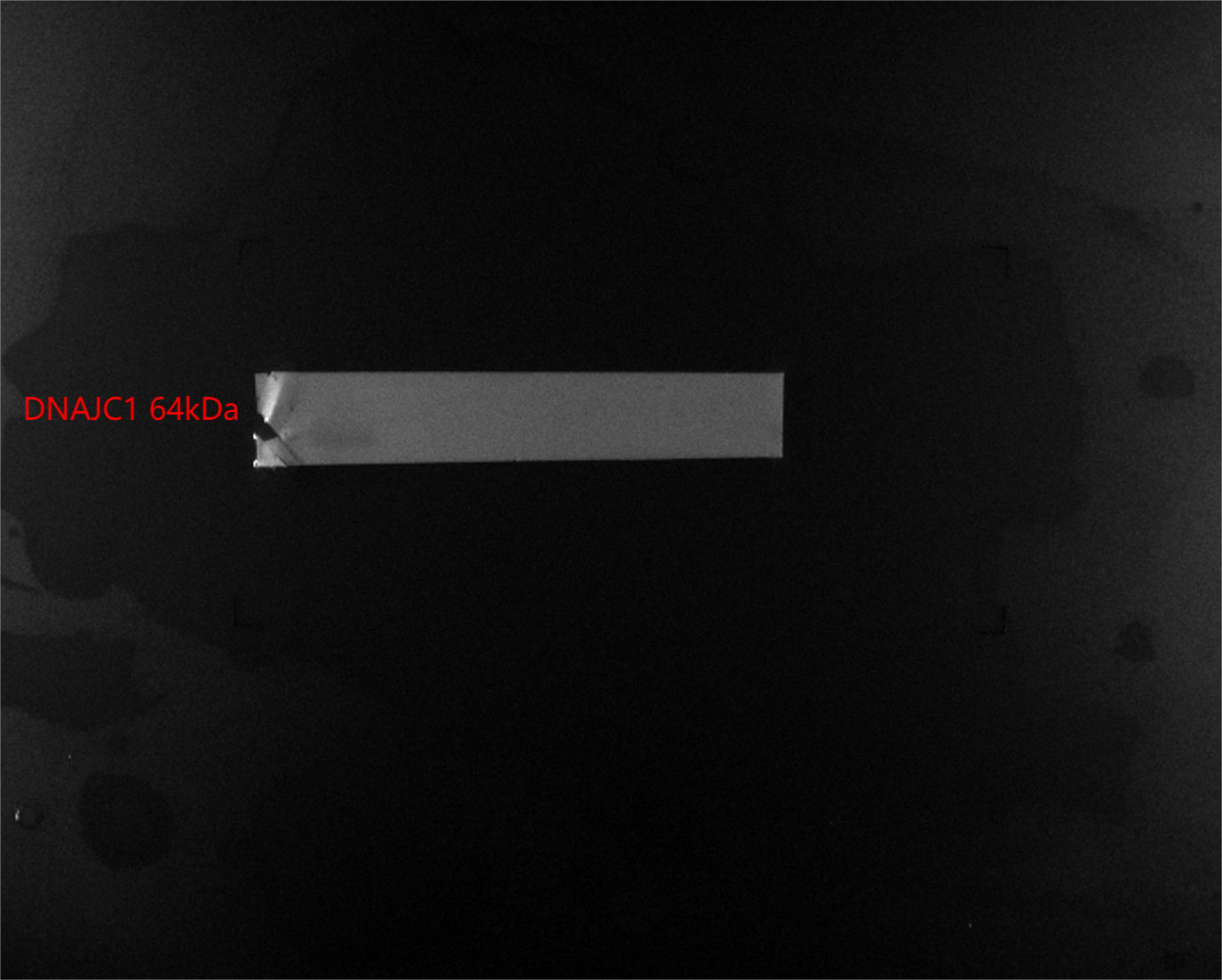

Supplement: Supplemental Information 1 [file peerj-11-15700-s001.zip › raw data 1-western blot/Original Image for Fig 2A/DNAJC1-original drawing.png]

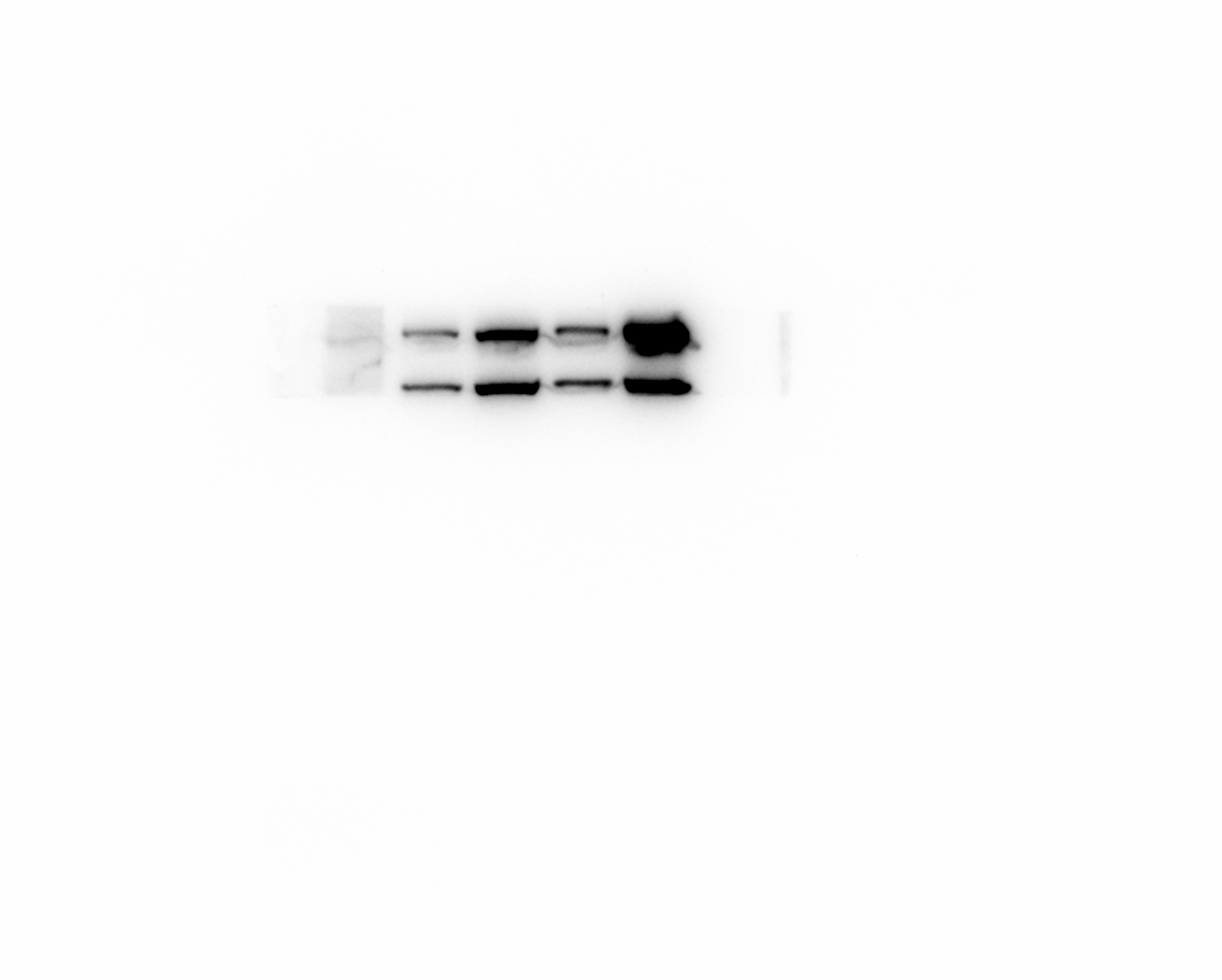

Supplement: Supplemental Information 1 [file peerj-11-15700-s001.zip › raw data 1-western blot/Original Image for Fig 2A/DNAJC1.tif]

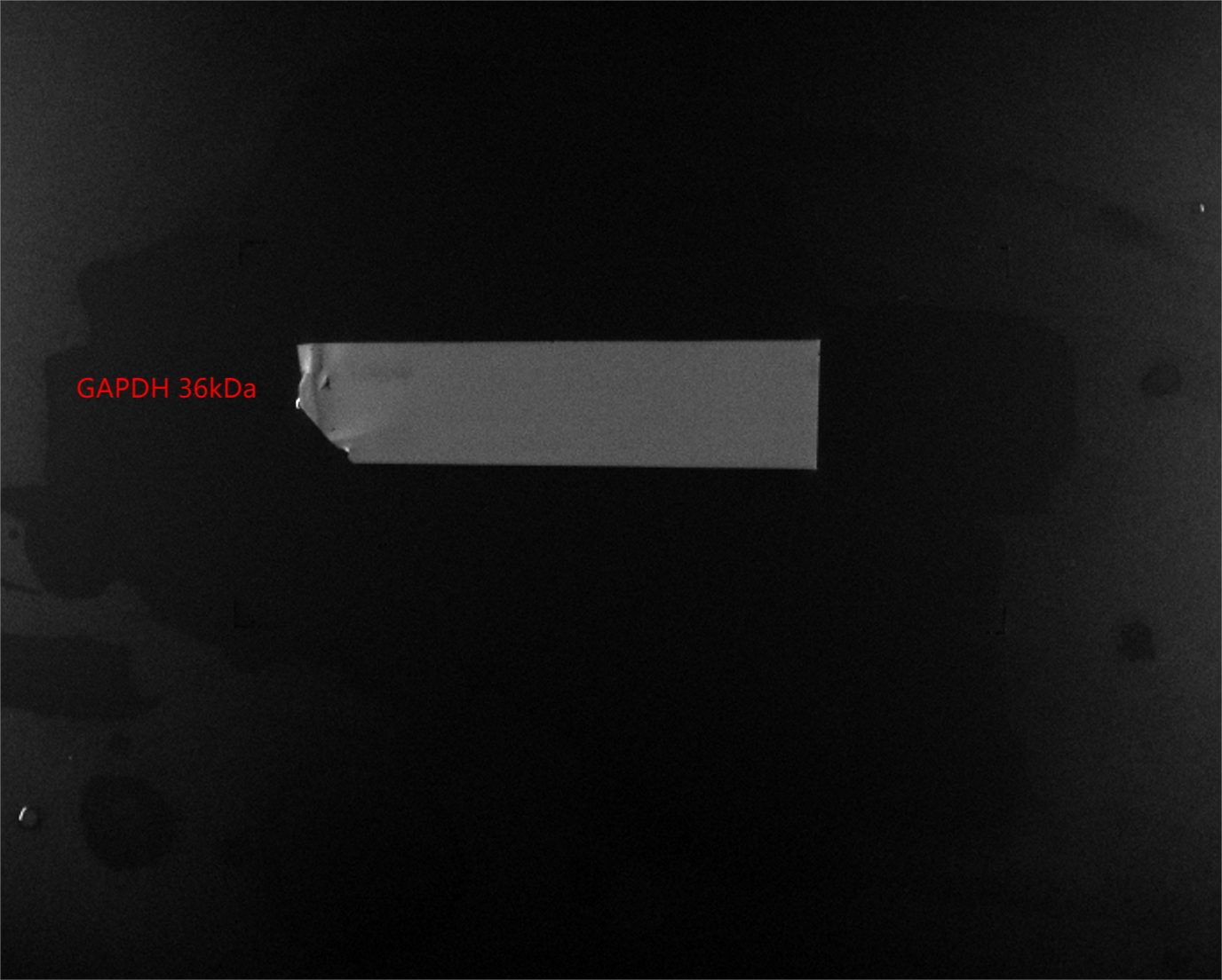

Supplement: Supplemental Information 1 [file peerj-11-15700-s001.zip › raw data 1-western blot/Original Image for Fig 2A/GAPDH-original drawing.png]

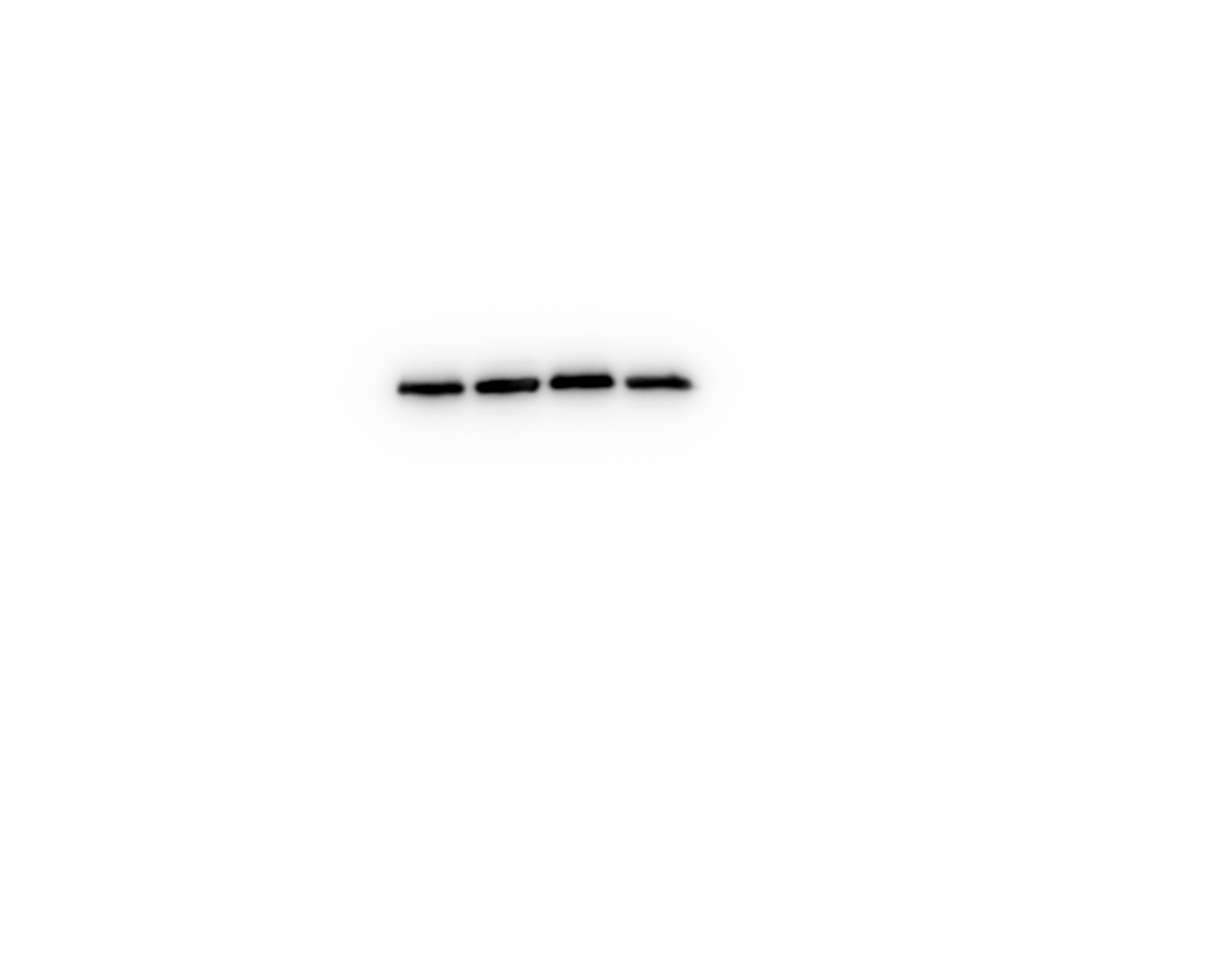

Supplement: Supplemental Information 1 [file peerj-11-15700-s001.zip › raw data 1-western blot/Original Image for Fig 2A/GAPDH.tif]

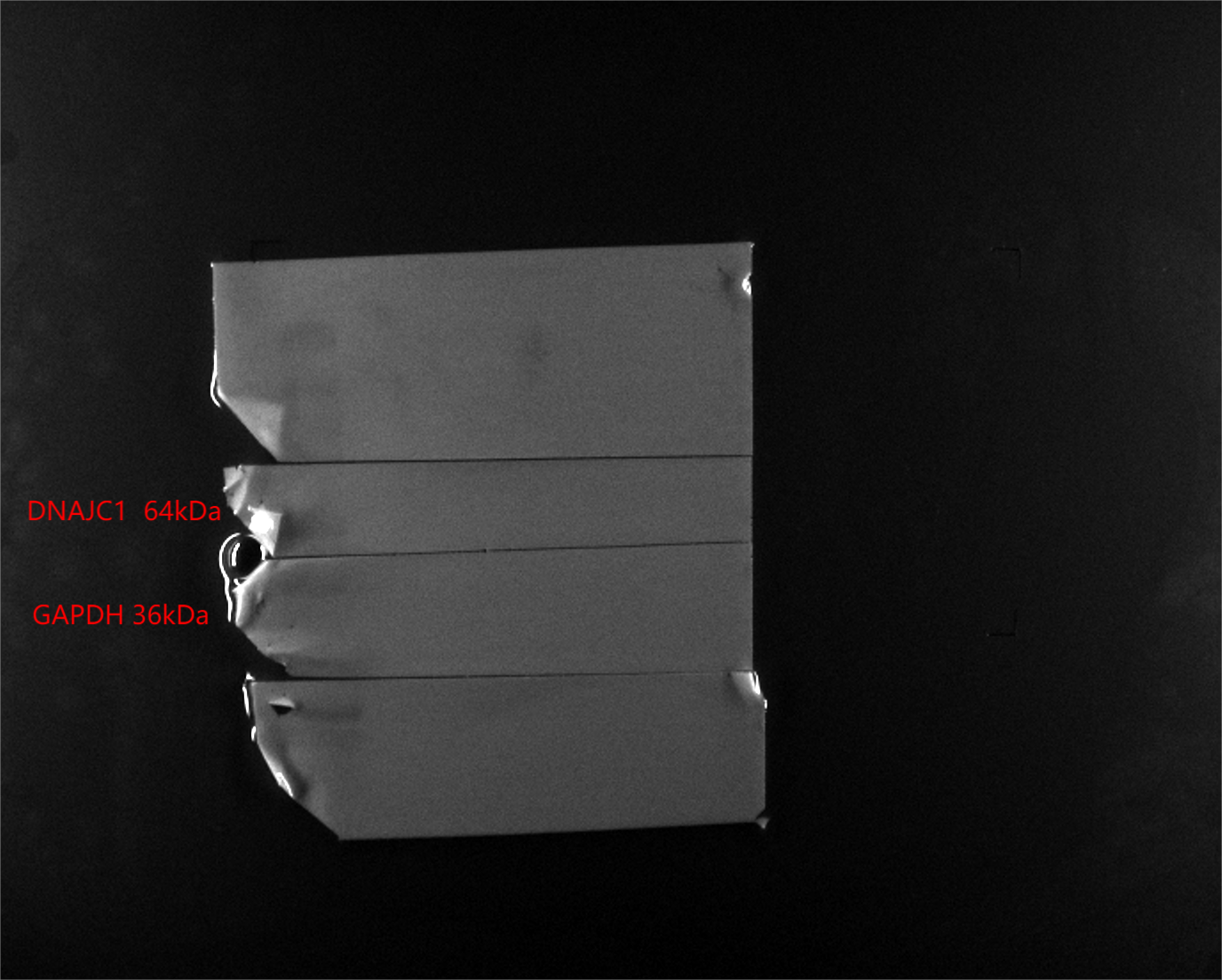

Supplement: Supplemental Information 1 [file peerj-11-15700-s001.zip › raw data 1-western blot/Original Image for Fig 2A/Intact membrane.png]

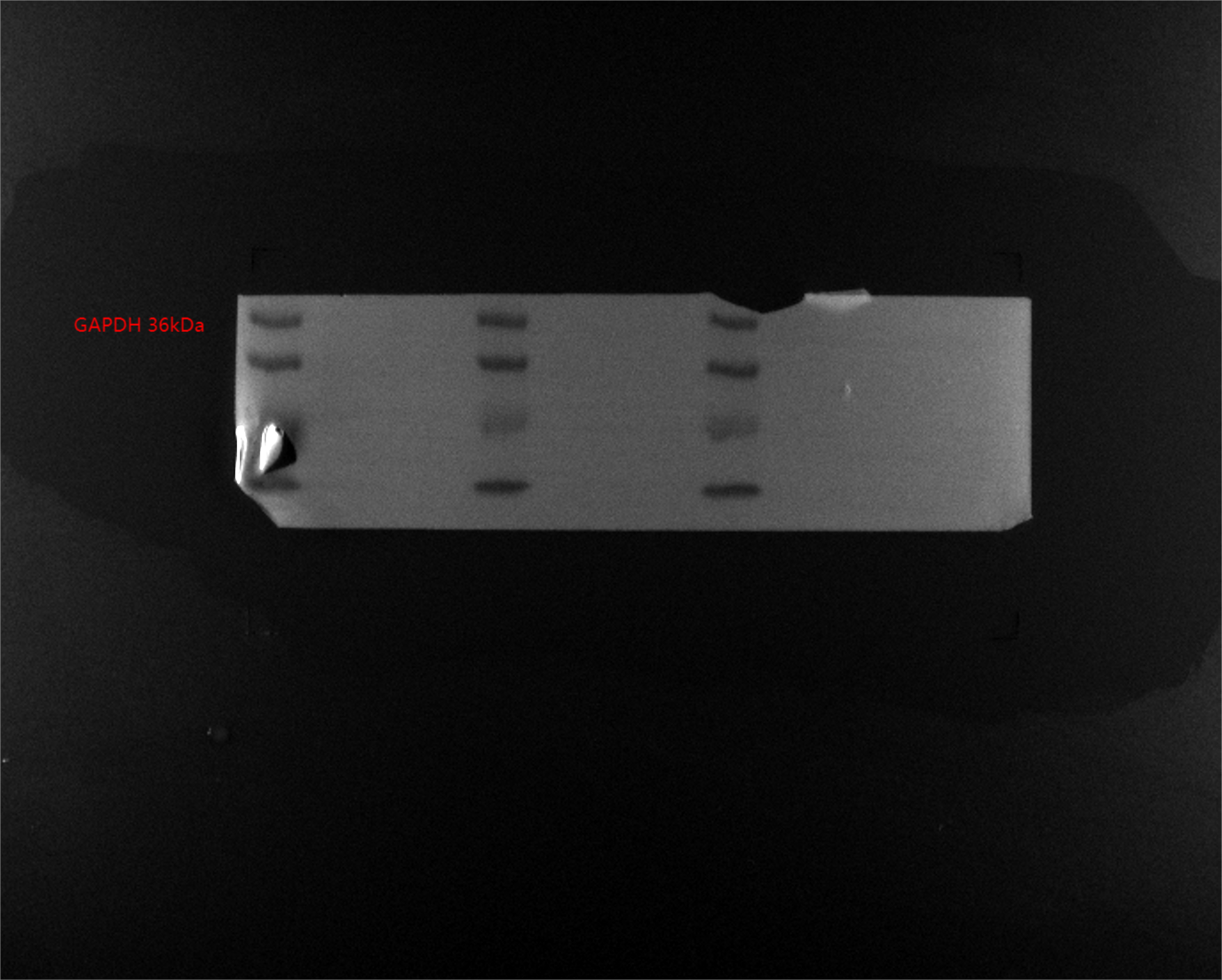

Supplement: Supplemental Information 1 [file peerj-11-15700-s001.zip › raw data 1-western blot/Original Image for Fig 3A/GAPDH-original drawing.png]

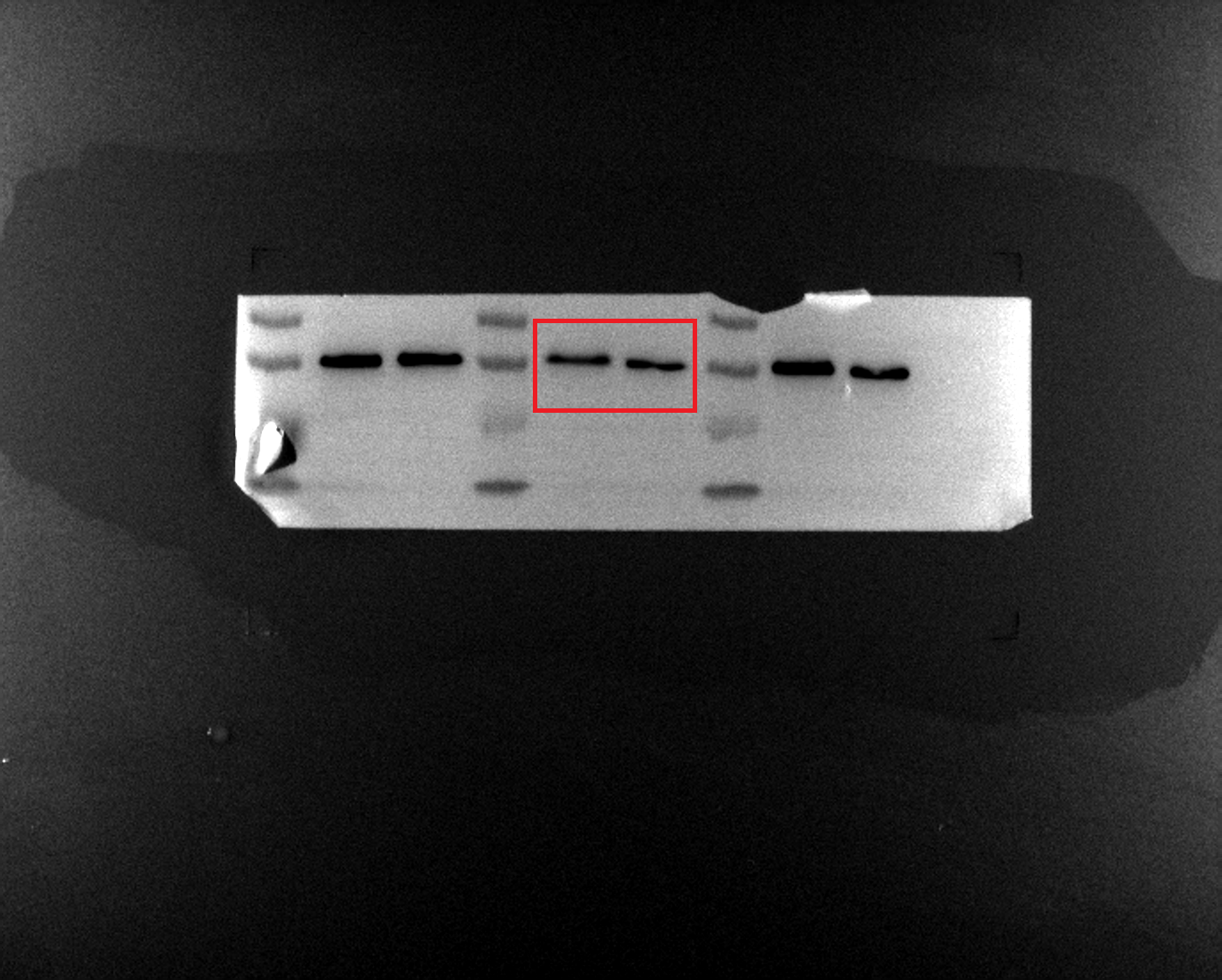

Supplement: Supplemental Information 1 [file peerj-11-15700-s001.zip › raw data 1-western blot/Original Image for Fig 3A/GAPDH.tif]

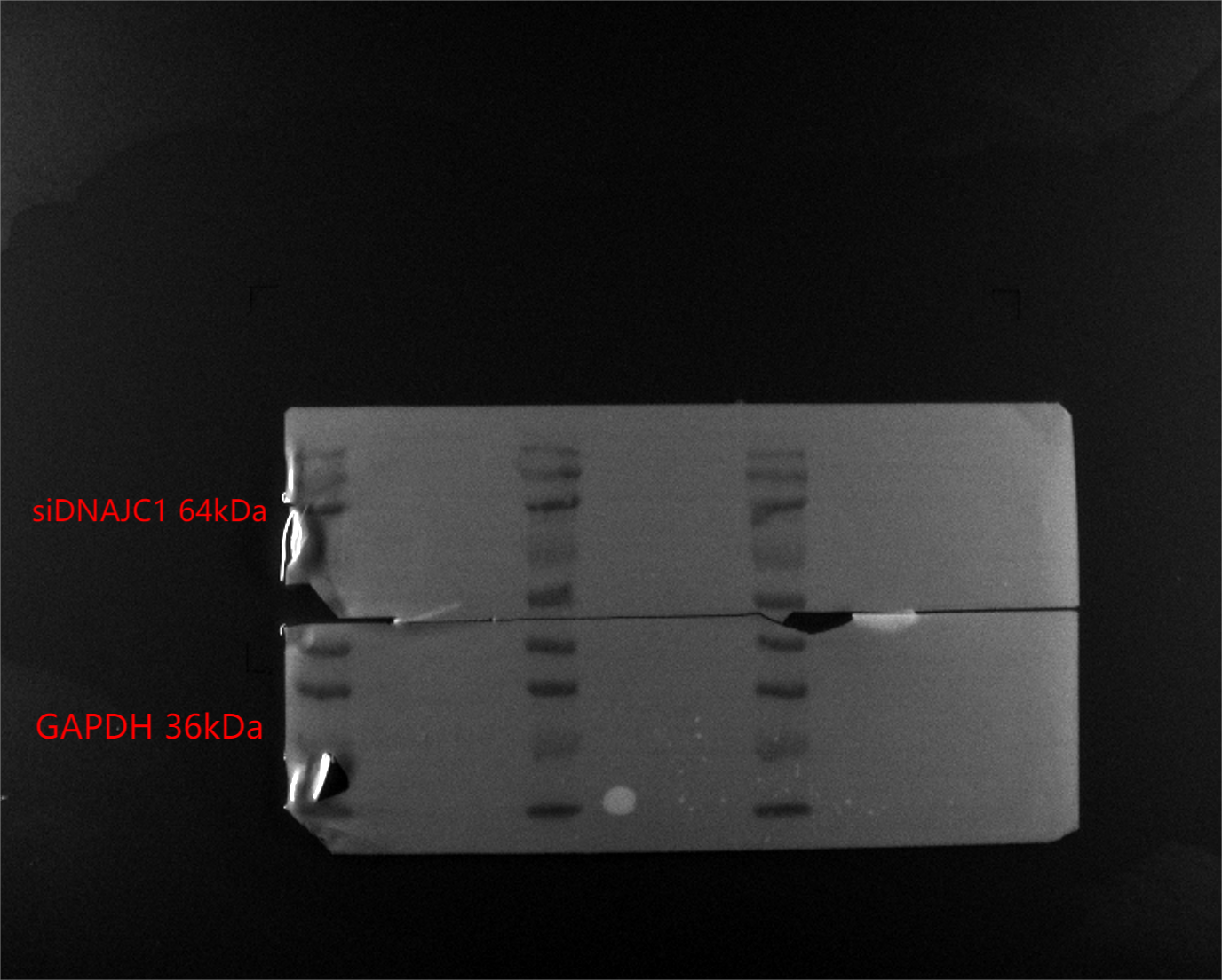

Supplement: Supplemental Information 1 [file peerj-11-15700-s001.zip › raw data 1-western blot/Original Image for Fig 3A/Intact membrane.png]

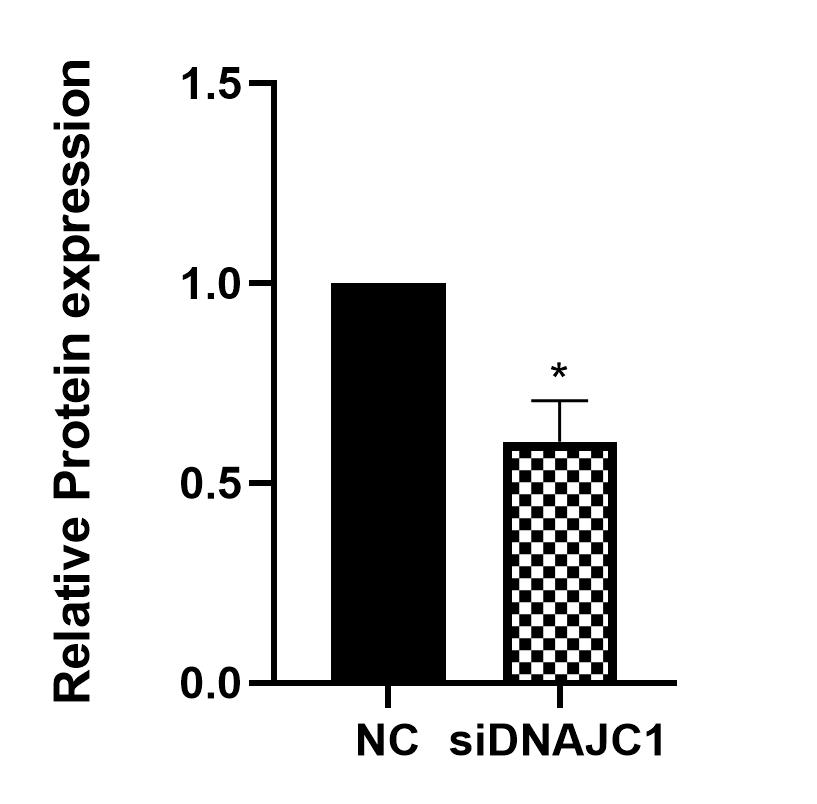

Supplement: Supplemental Information 1 [file peerj-11-15700-s001.zip › raw data 1-western blot/Original Image for Fig 3A/Relative Protein Expression.jpg]

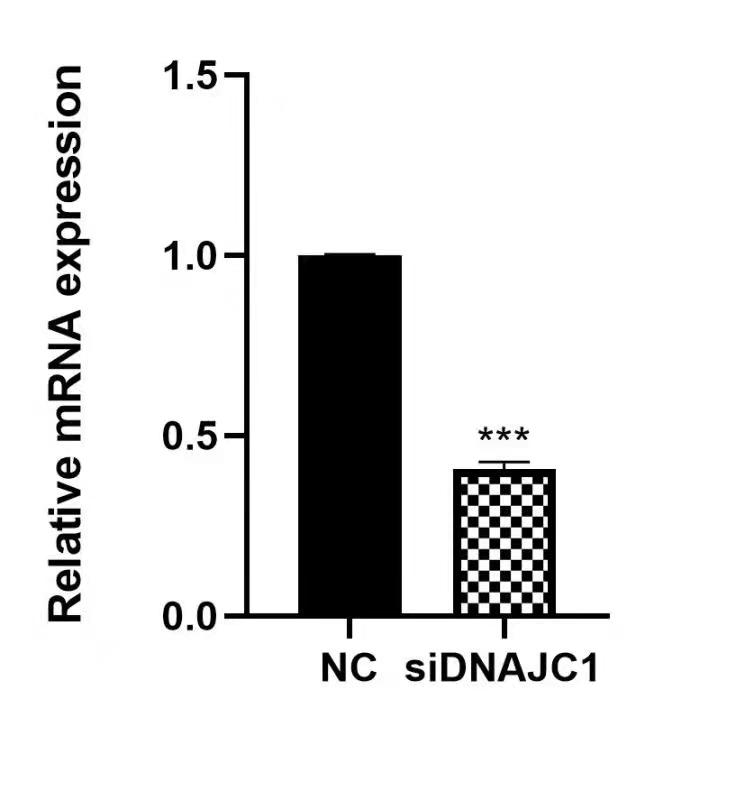

Supplement: Supplemental Information 1 [file peerj-11-15700-s001.zip › raw data 1-western blot/Original Image for Fig 3A/Relative mRNA Expression.jpg]

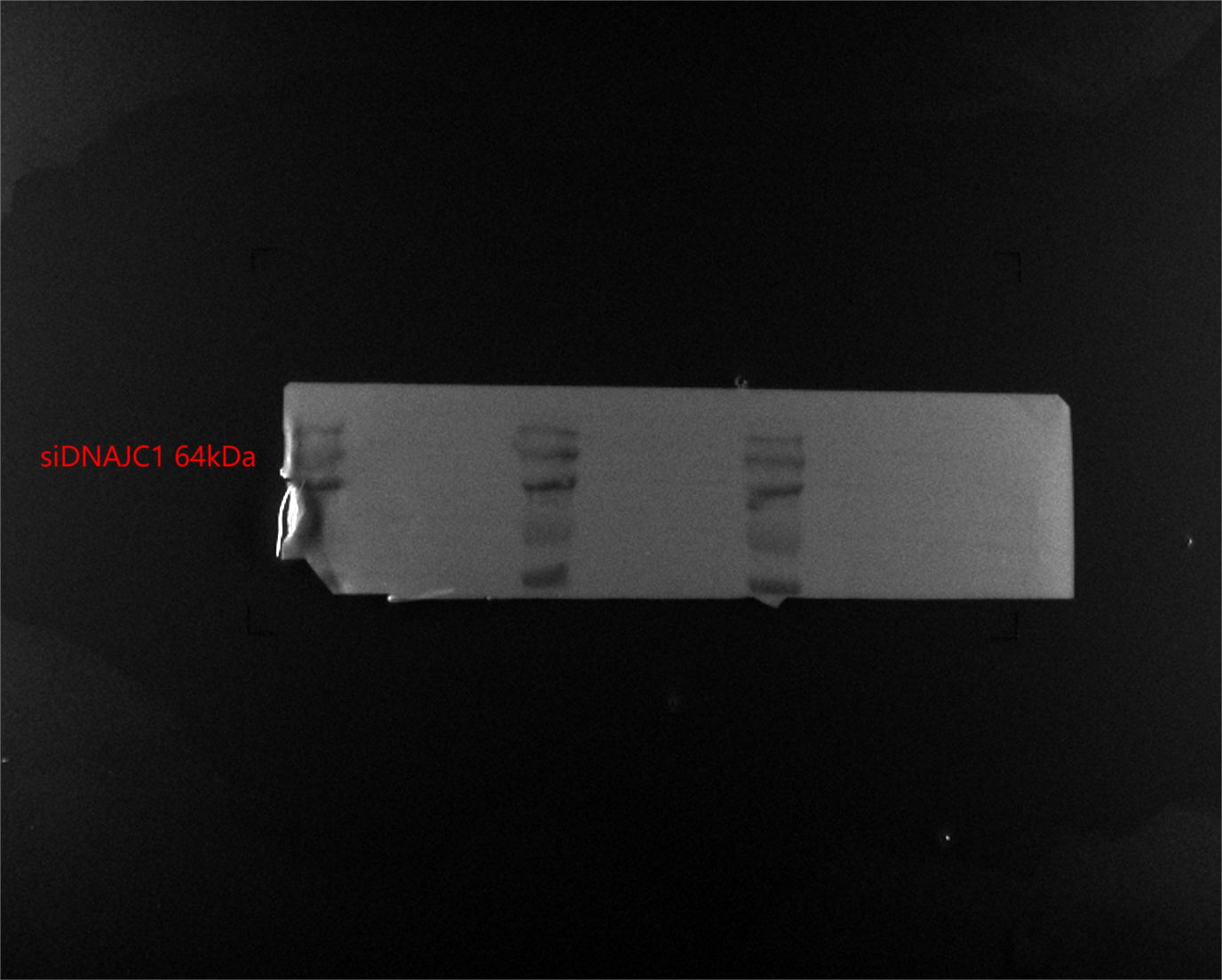

Supplement: Supplemental Information 1 [file peerj-11-15700-s001.zip › raw data 1-western blot/Original Image for Fig 3A/siDNAJC1-original drawing.png]

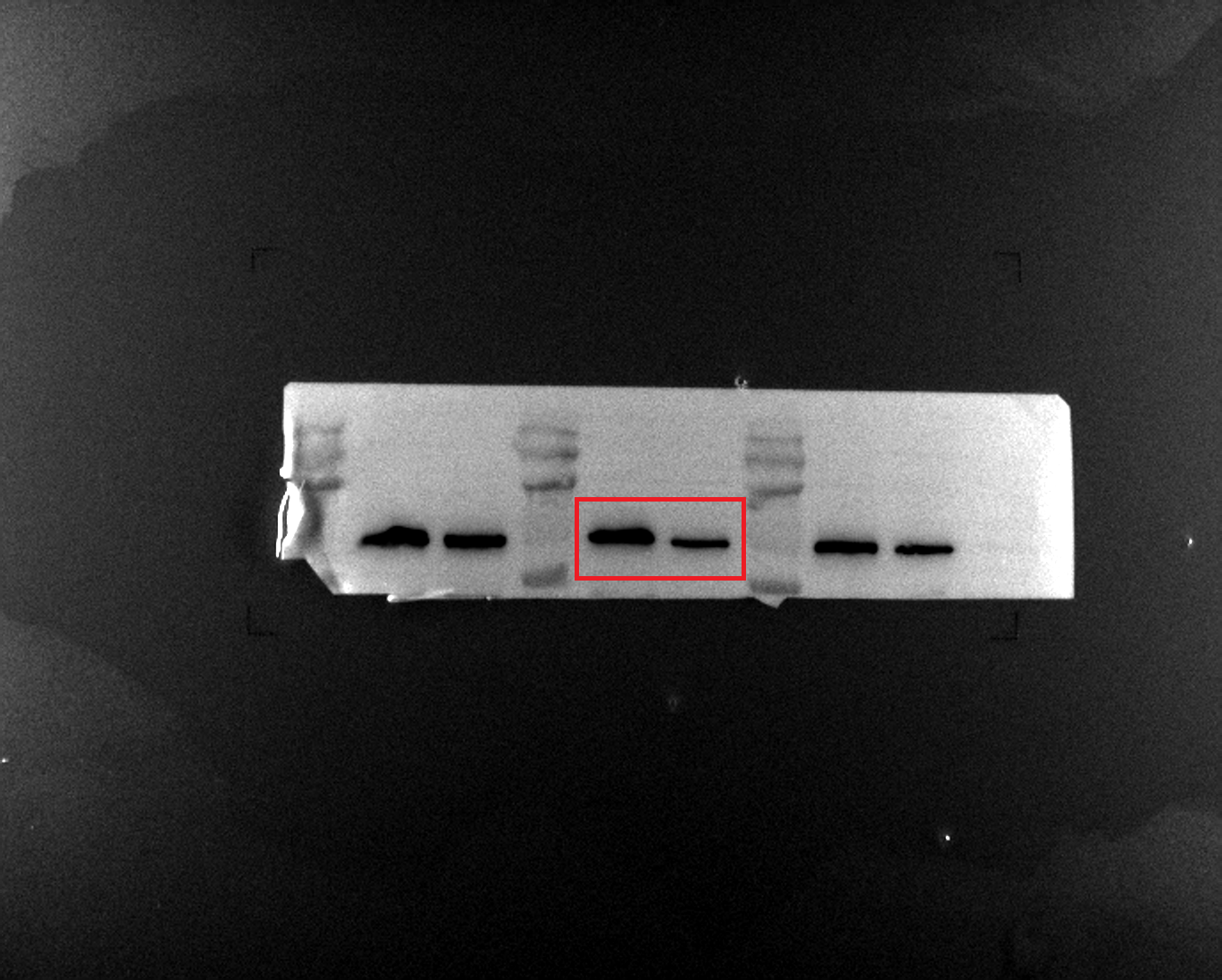

Supplement: Supplemental Information 1 [file peerj-11-15700-s001.zip › raw data 1-western blot/Original Image for Fig 3A/siDNAJC1.tif]

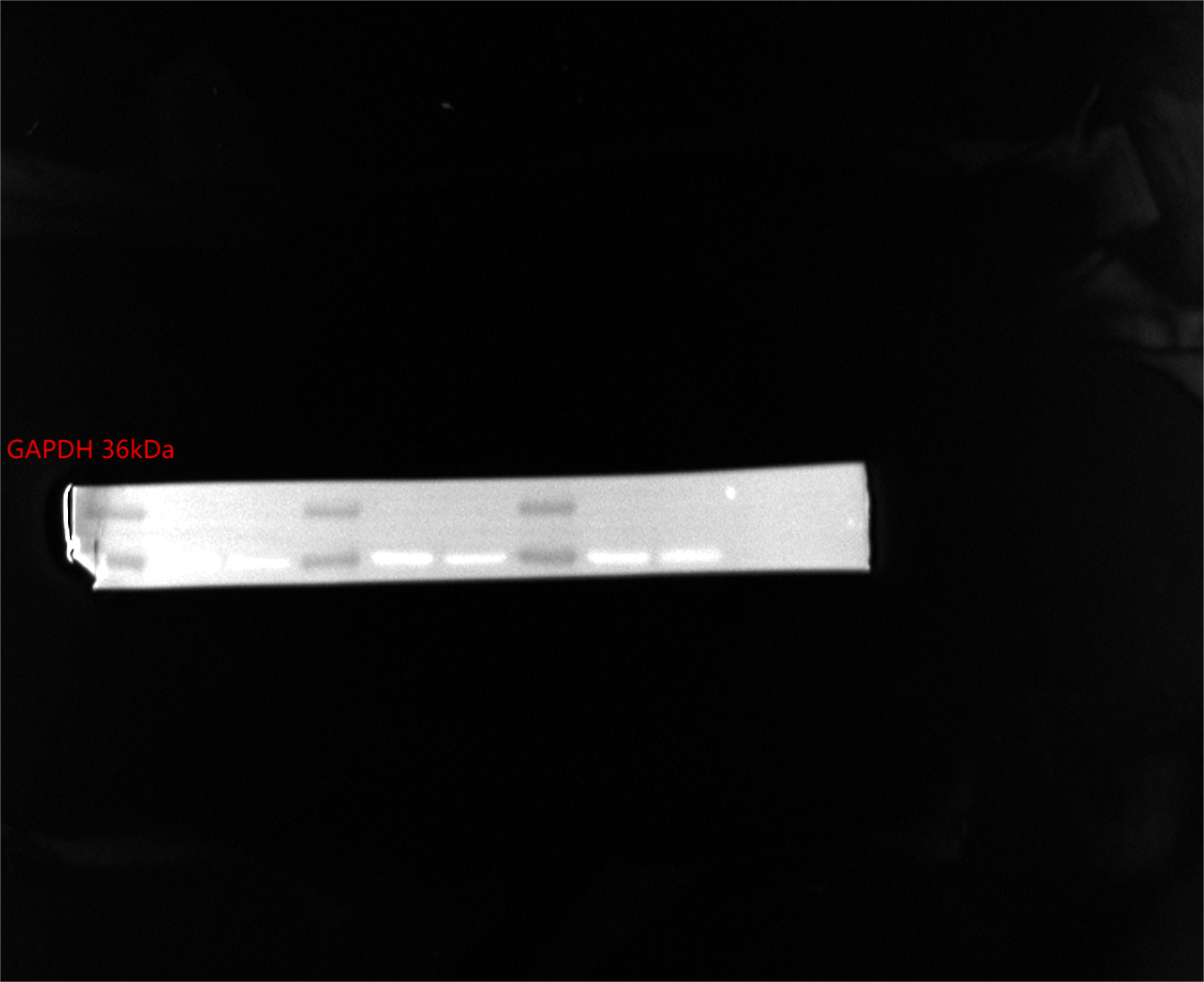

Supplement: Supplemental Information 1 [file peerj-11-15700-s001.zip › raw data 1-western blot/Original Image for Fig 3B/GAPDH-original drawing.png]

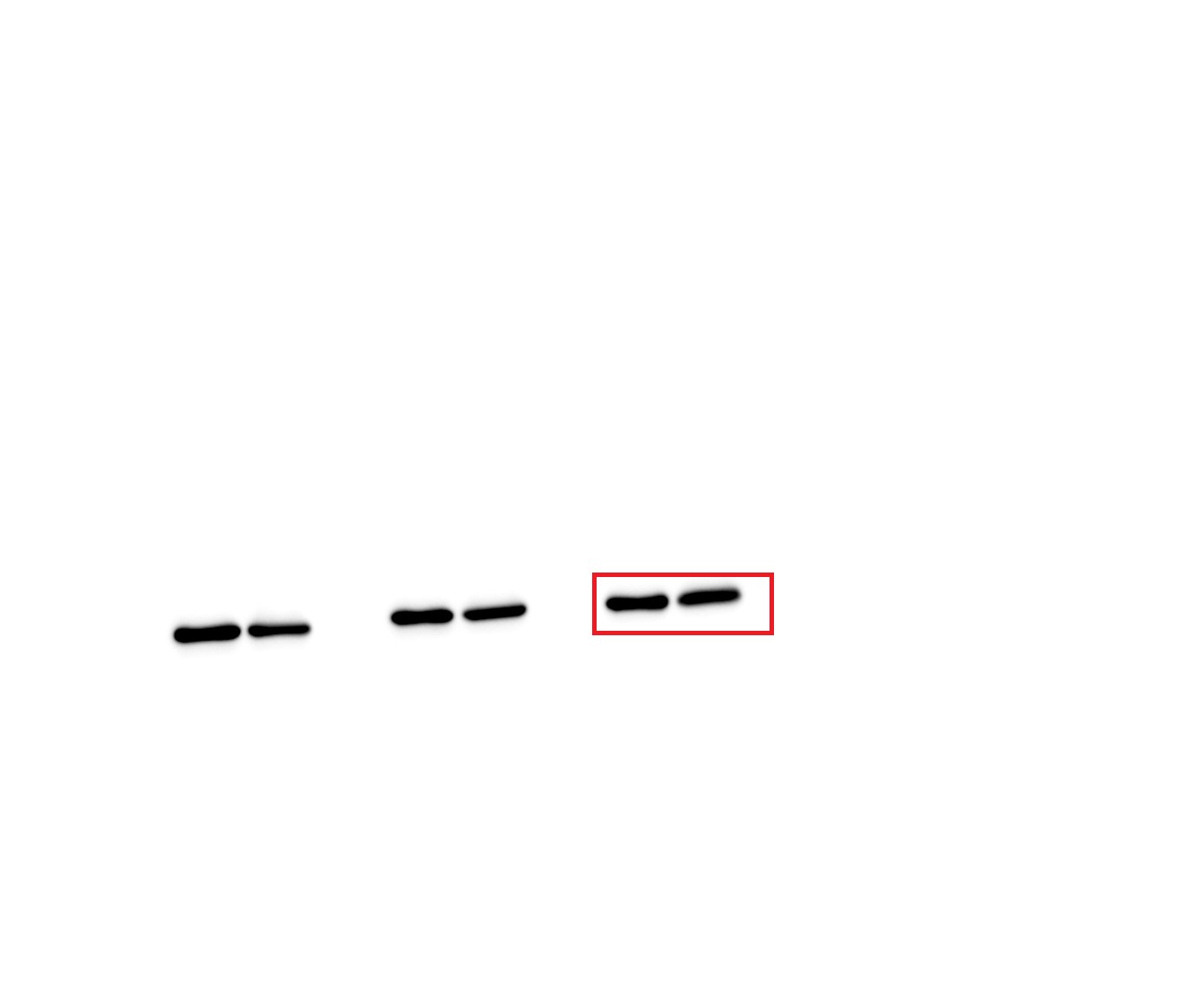

Supplement: Supplemental Information 1 [file peerj-11-15700-s001.zip › raw data 1-western blot/Original Image for Fig 3B/GAPDH.Tif]

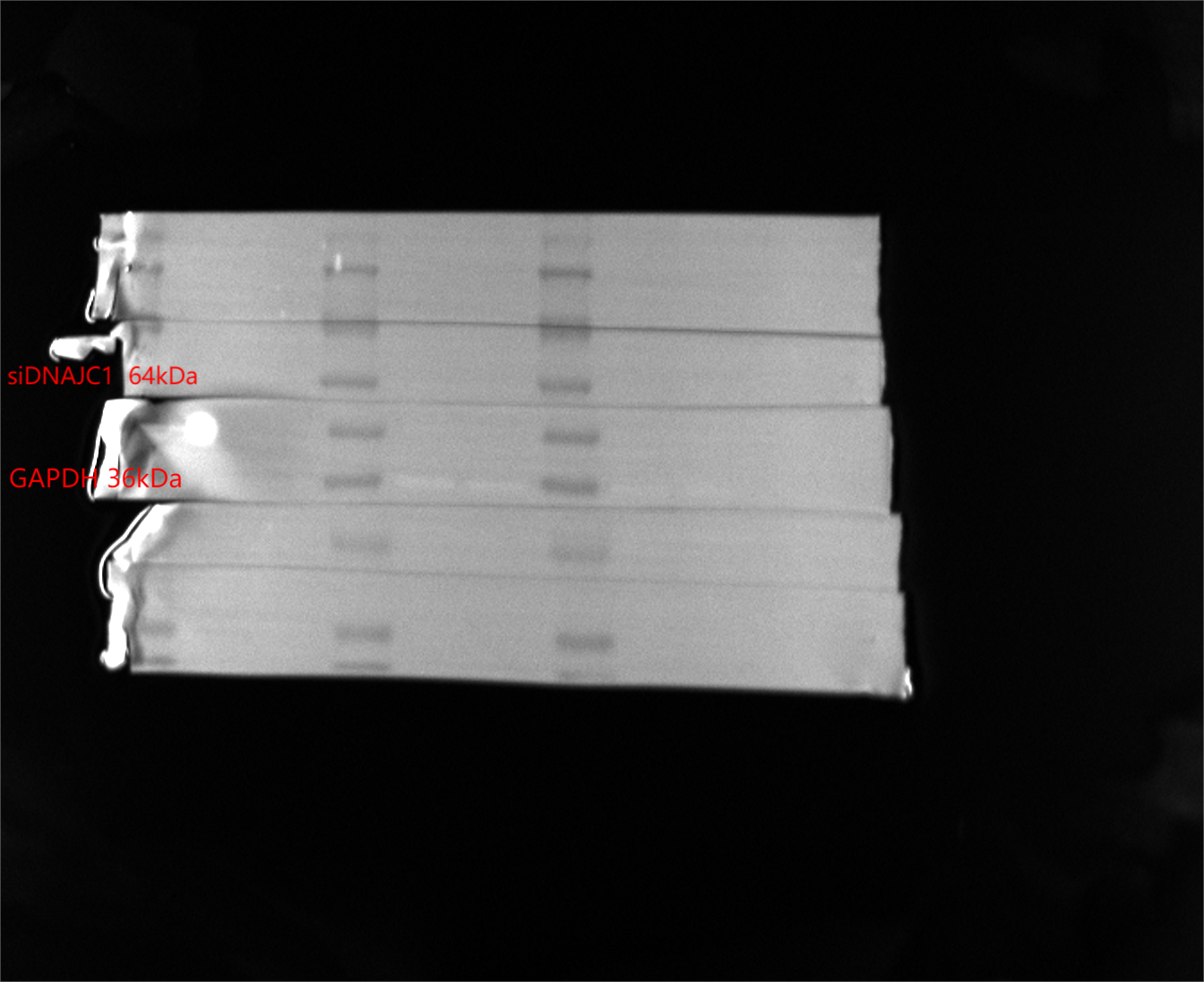

Supplement: Supplemental Information 1 [file peerj-11-15700-s001.zip › raw data 1-western blot/Original Image for Fig 3B/Intact membrane.png]

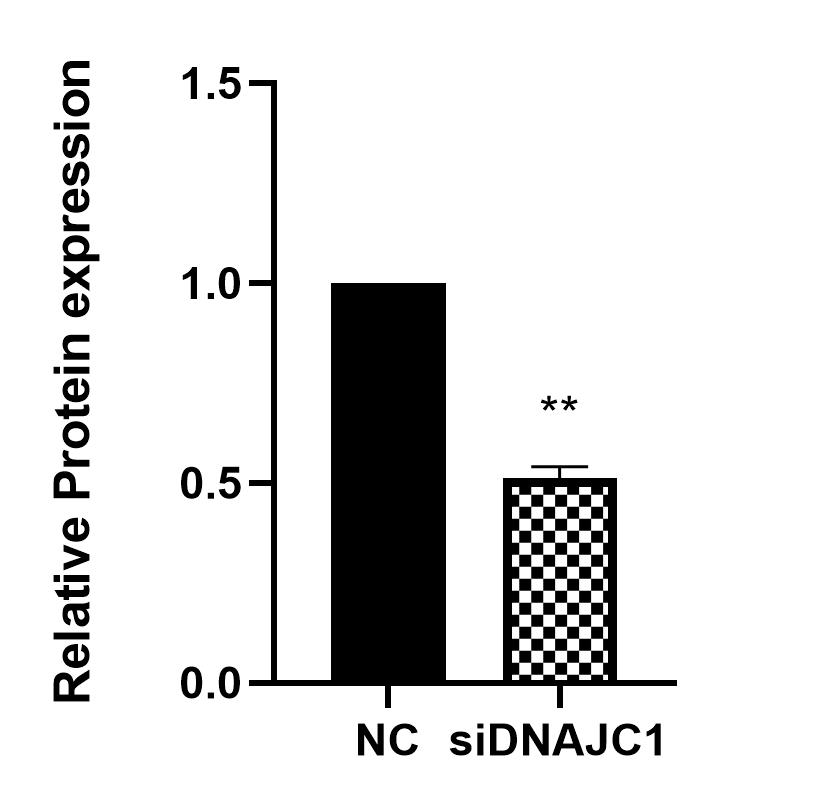

Supplement: Supplemental Information 1 [file peerj-11-15700-s001.zip › raw data 1-western blot/Original Image for Fig 3B/Relative Protein Expression.jpg]

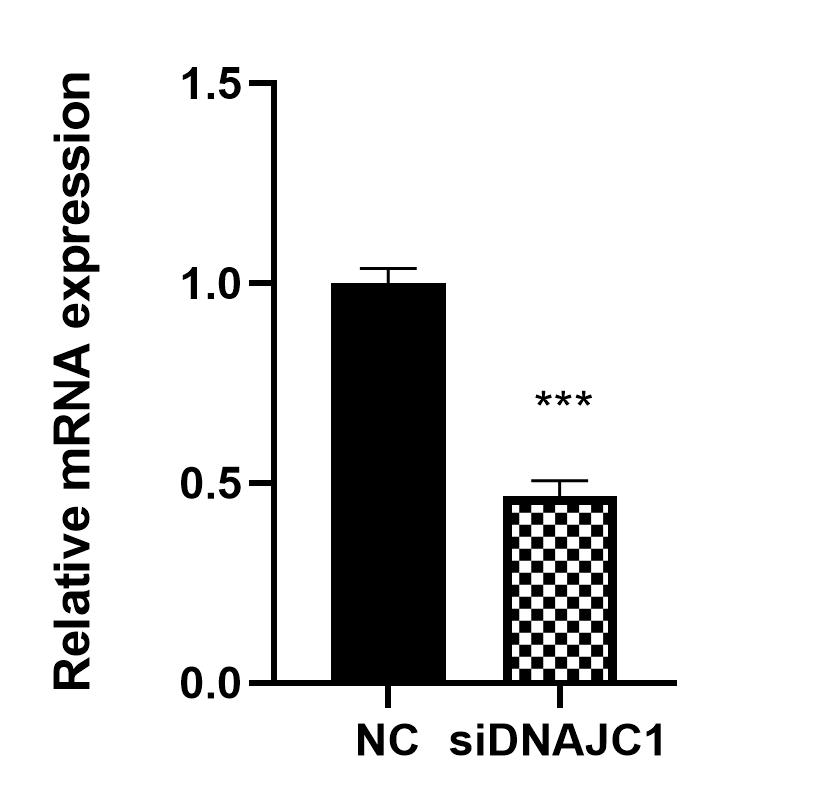

Supplement: Supplemental Information 1 [file peerj-11-15700-s001.zip › raw data 1-western blot/Original Image for Fig 3B/Relative mRNA Expression.jpg]

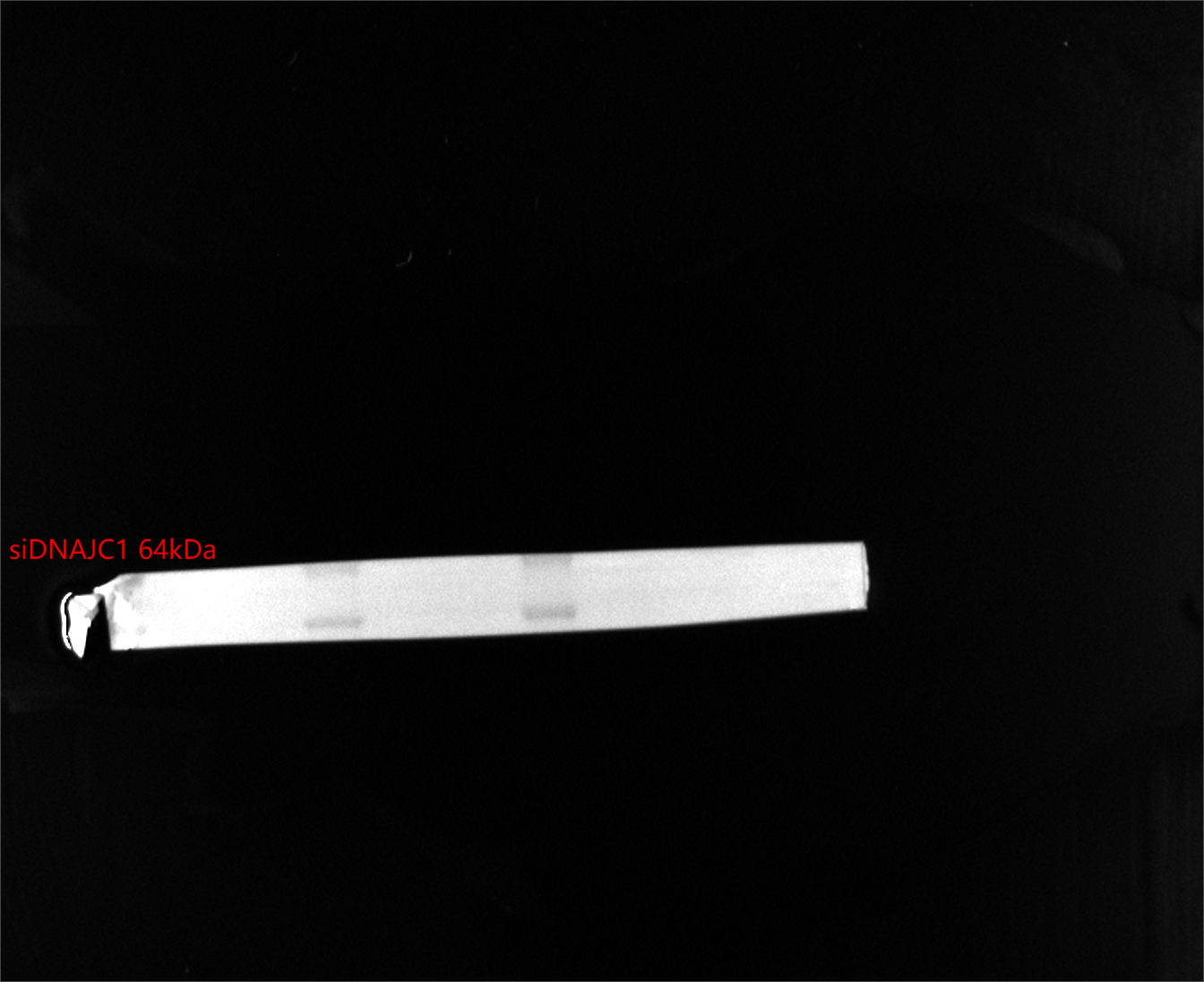

Supplement: Supplemental Information 1 [file peerj-11-15700-s001.zip › raw data 1-western blot/Original Image for Fig 3B/siDNAJC1-original drawing.png]

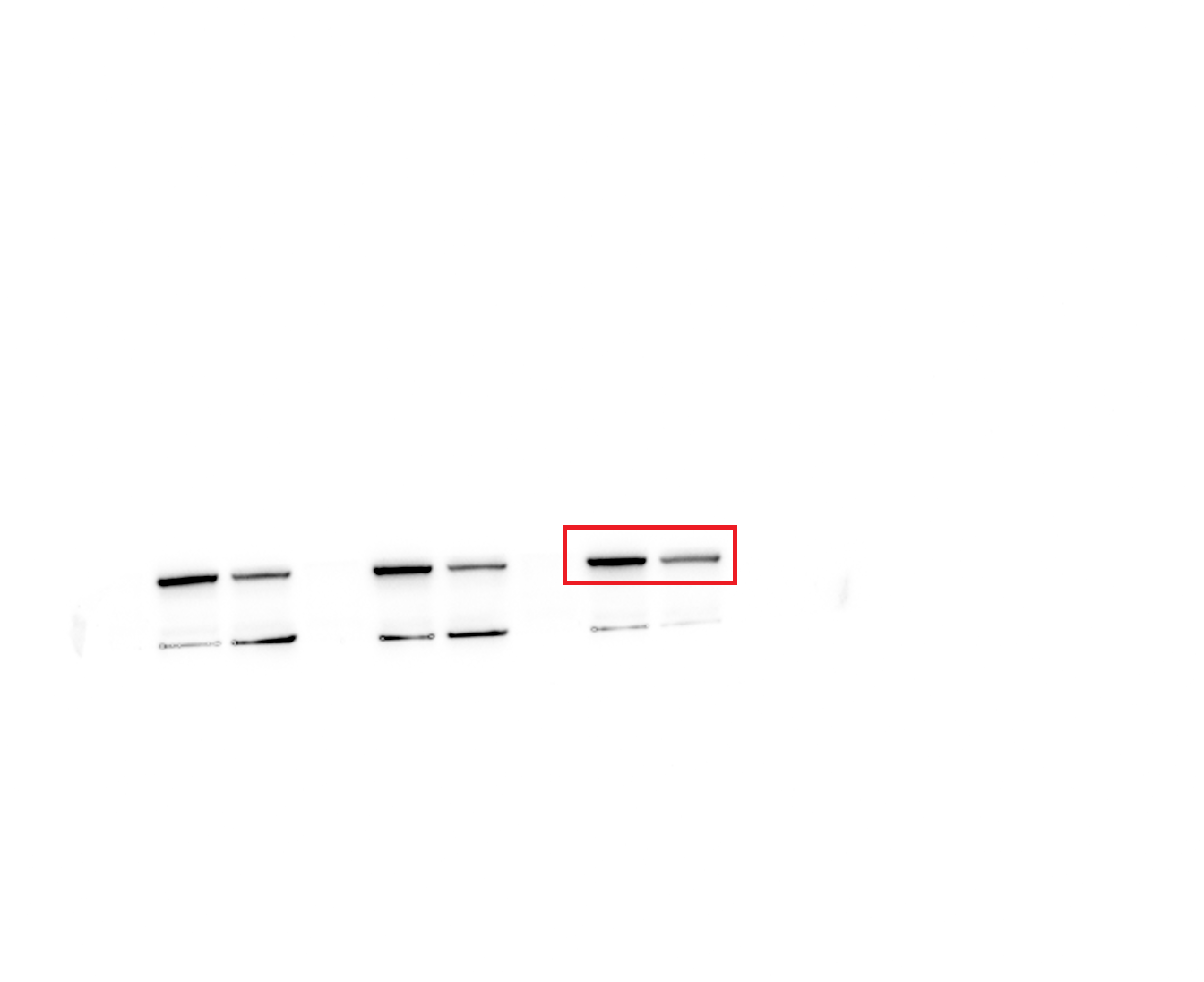

Supplement: Supplemental Information 1 [file peerj-11-15700-s001.zip › raw data 1-western blot/Original Image for Fig 3B/siDNAJC1.Tif]

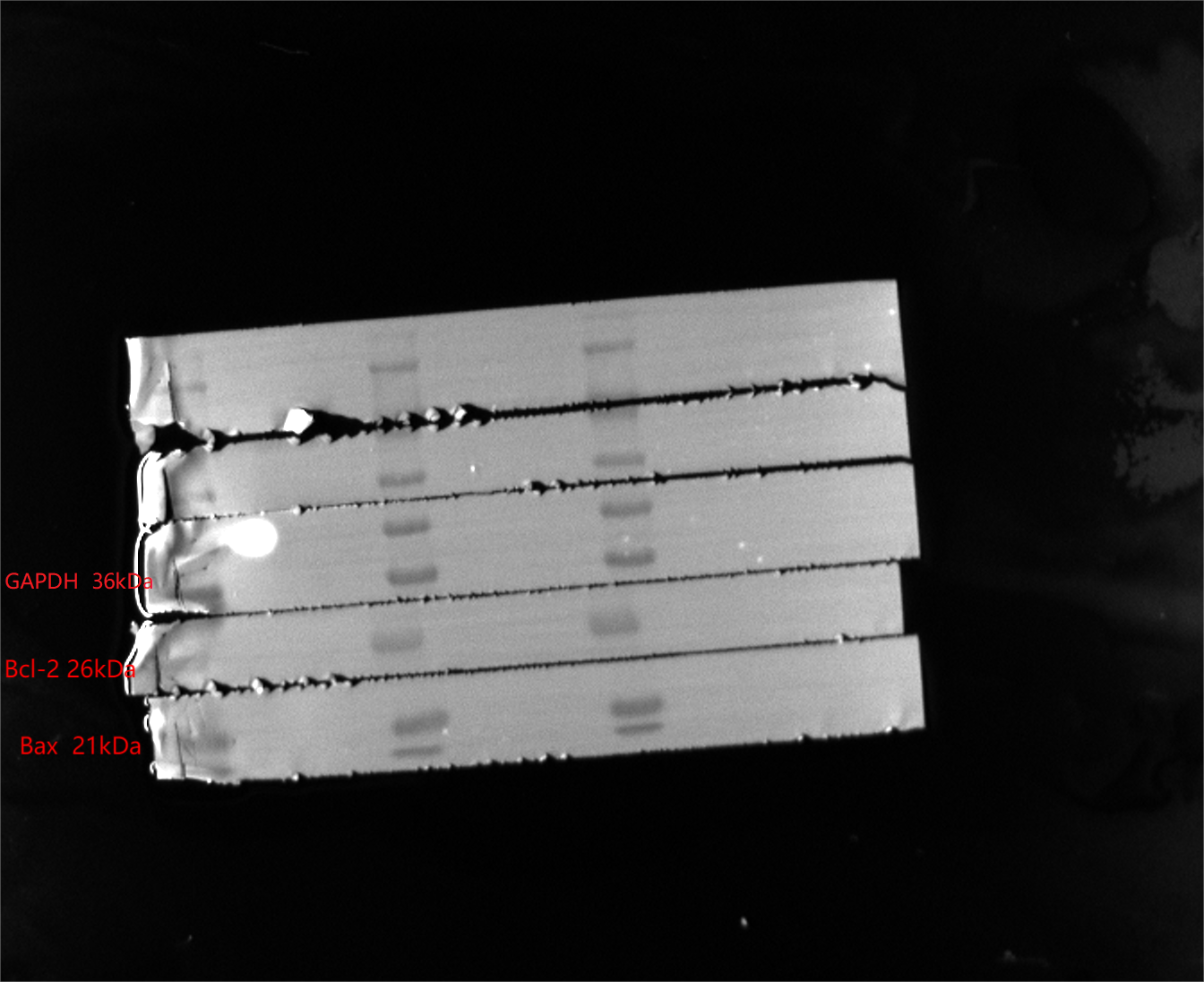

Supplement: Supplemental Information 1 [file peerj-11-15700-s001.zip › raw data 1-western blot/Original Image for Fig 6B/Bax ,Bcl-2 and GAPDH intact membrane .png]

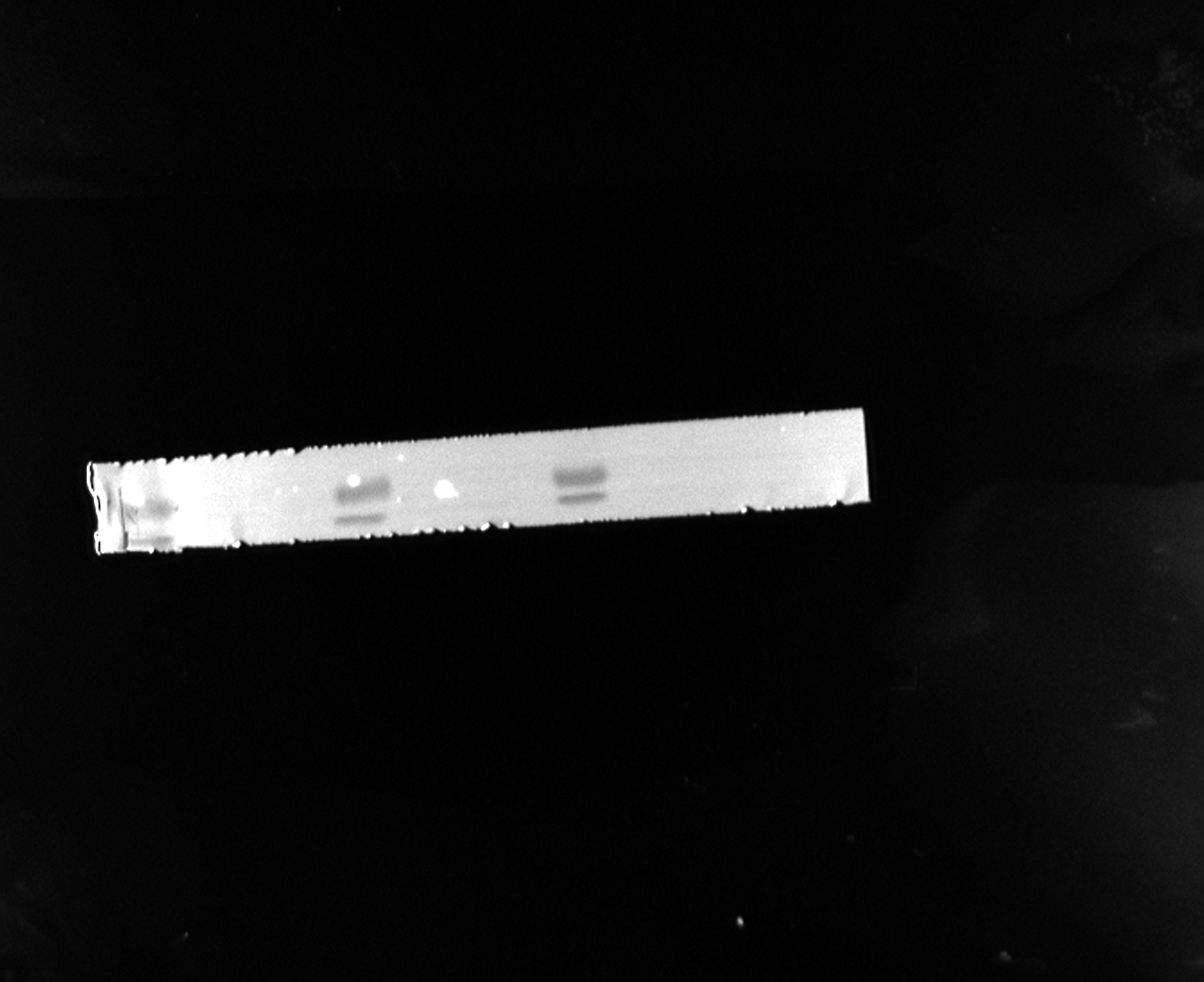

Supplement: Supplemental Information 1 [file peerj-11-15700-s001.zip › raw data 1-western blot/Original Image for Fig 6B/Bax-original drawing.Tif]

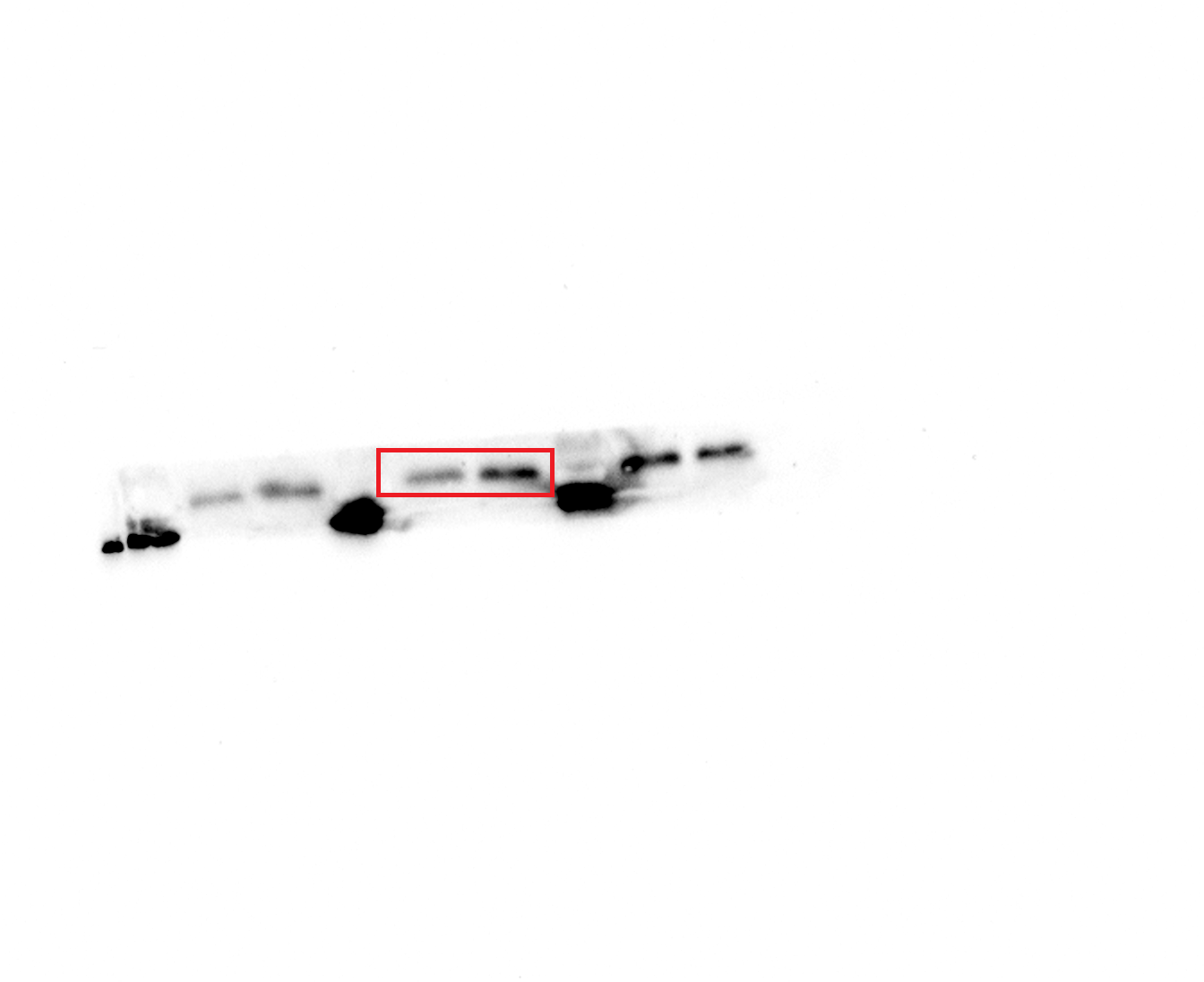

Supplement: Supplemental Information 1 [file peerj-11-15700-s001.zip › raw data 1-western blot/Original Image for Fig 6B/Bax.Tif]

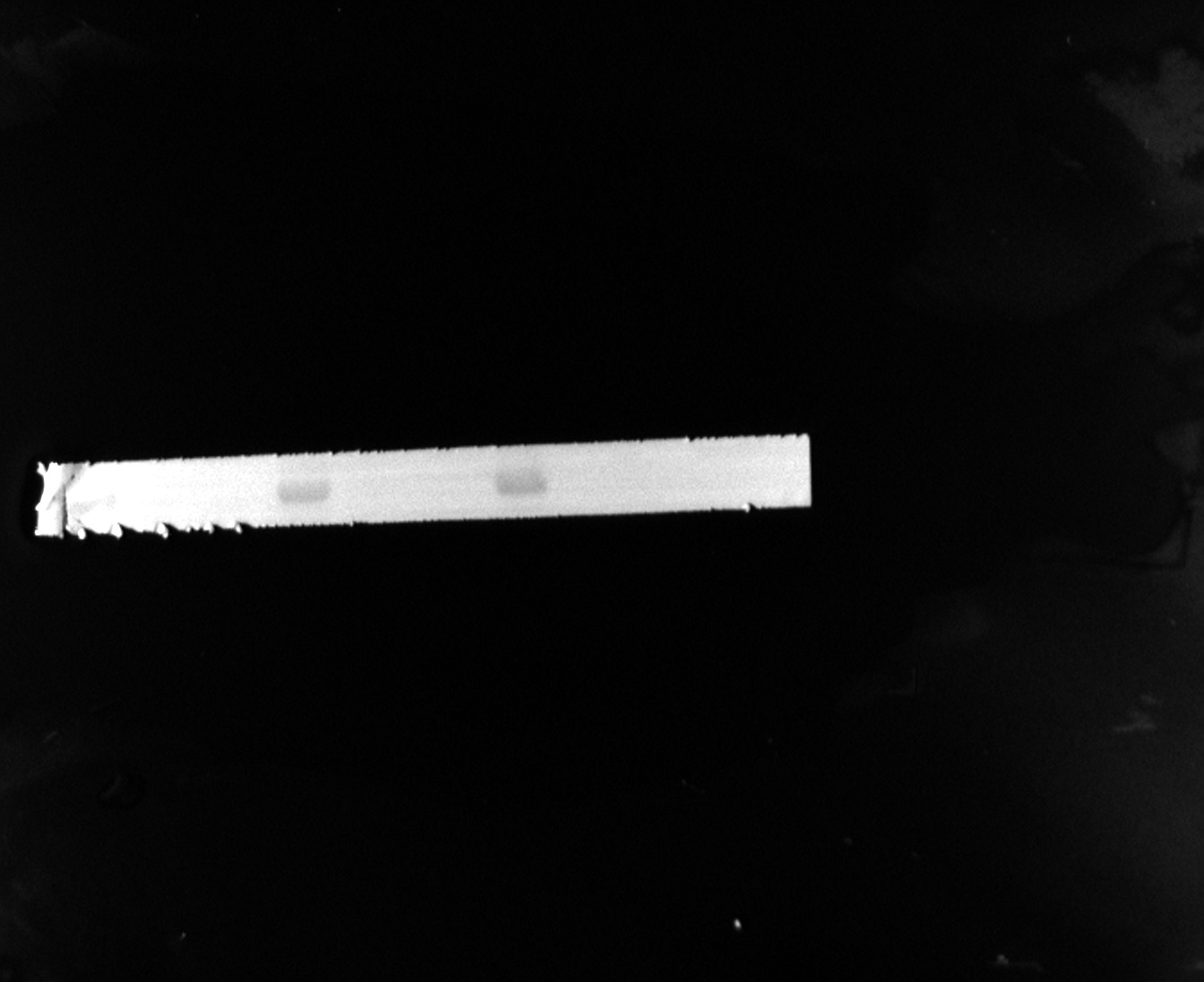

Supplement: Supplemental Information 1 [file peerj-11-15700-s001.zip › raw data 1-western blot/Original Image for Fig 6B/Bcl-2-original drawing.Tif]

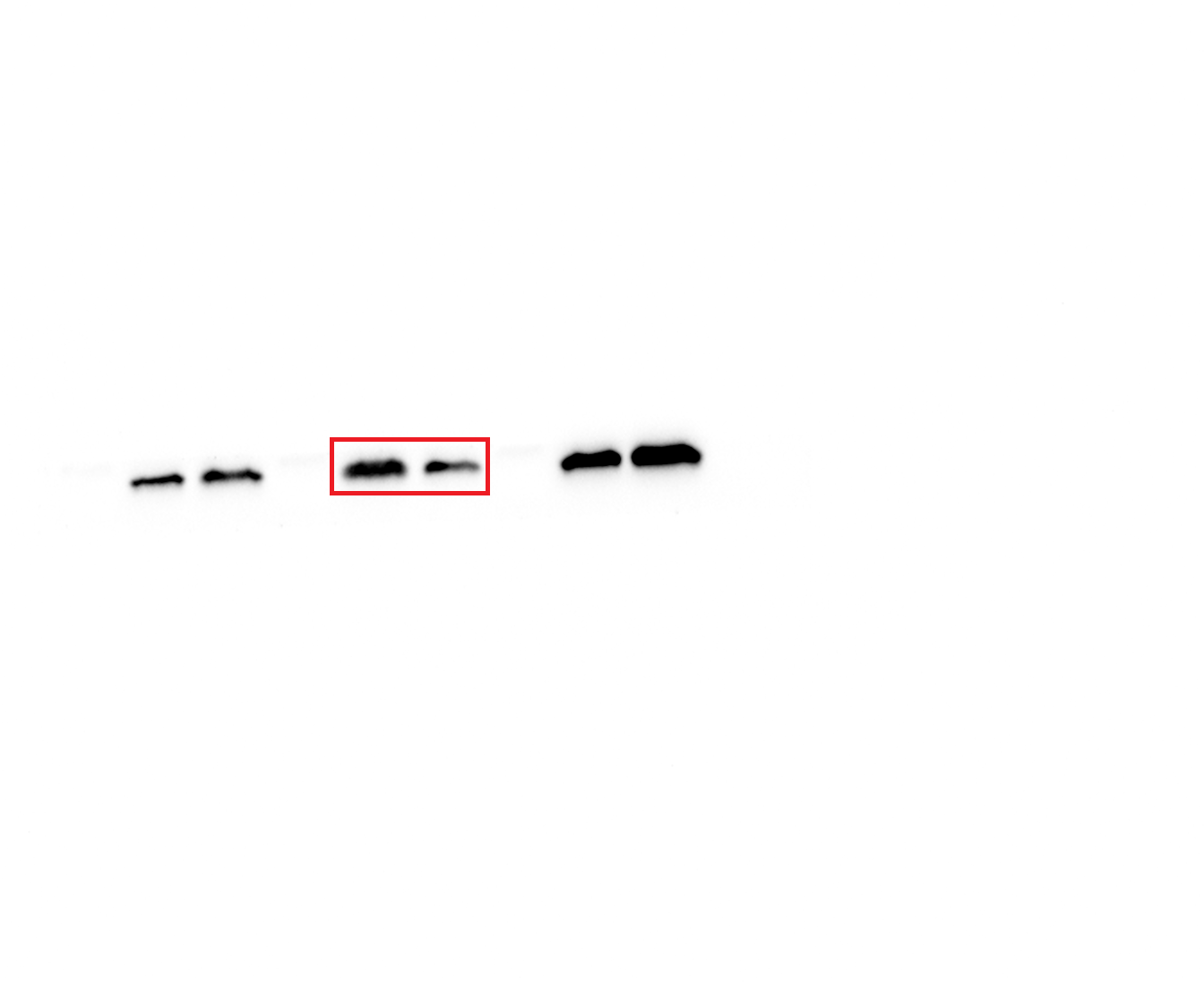

Supplement: Supplemental Information 1 [file peerj-11-15700-s001.zip › raw data 1-western blot/Original Image for Fig 6B/Bcl-2.Tif]

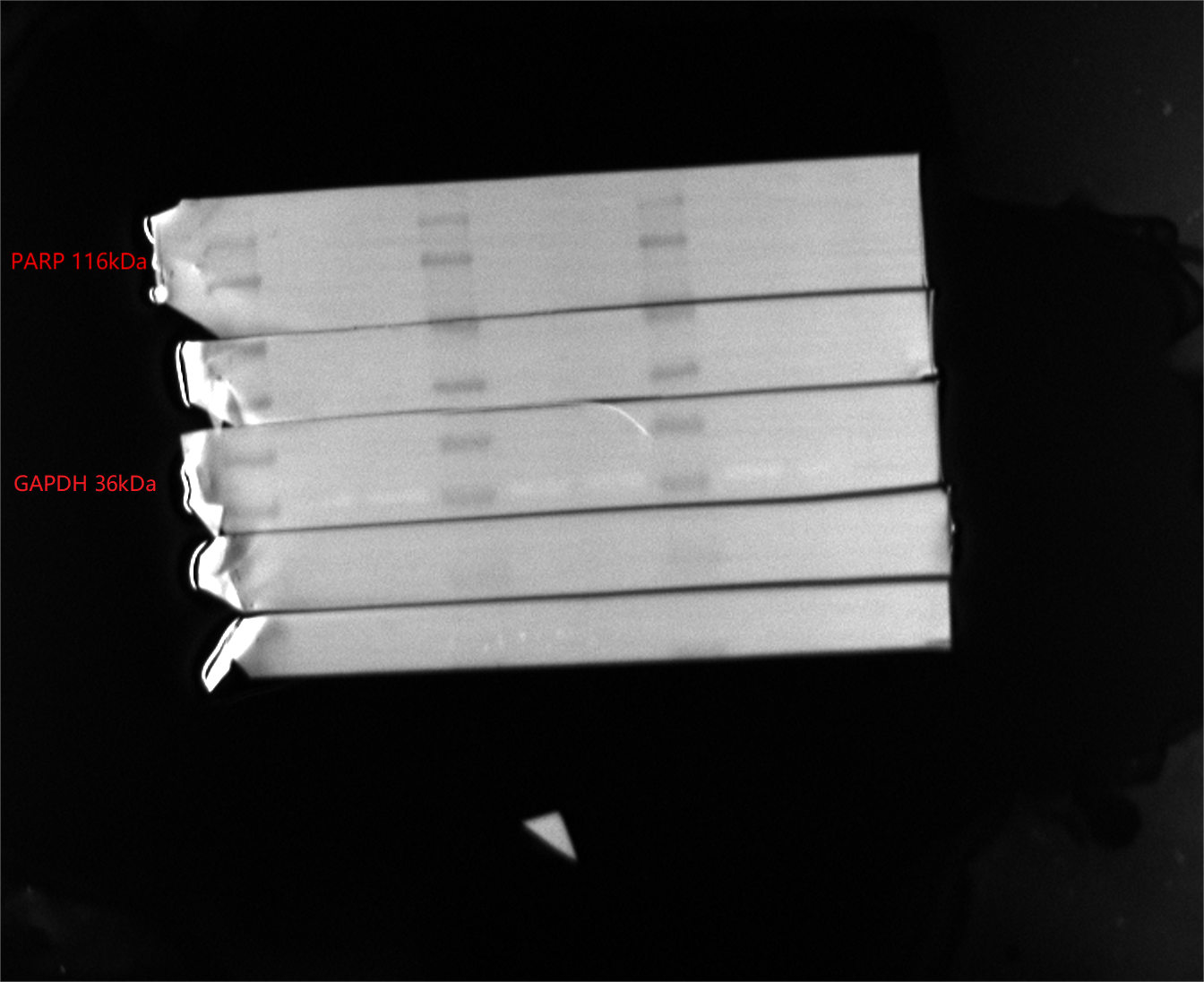

Supplement: Supplemental Information 1 [file peerj-11-15700-s001.zip › raw data 1-western blot/Original Image for Fig 6B/PARP and GAPDH intact membrane.png]

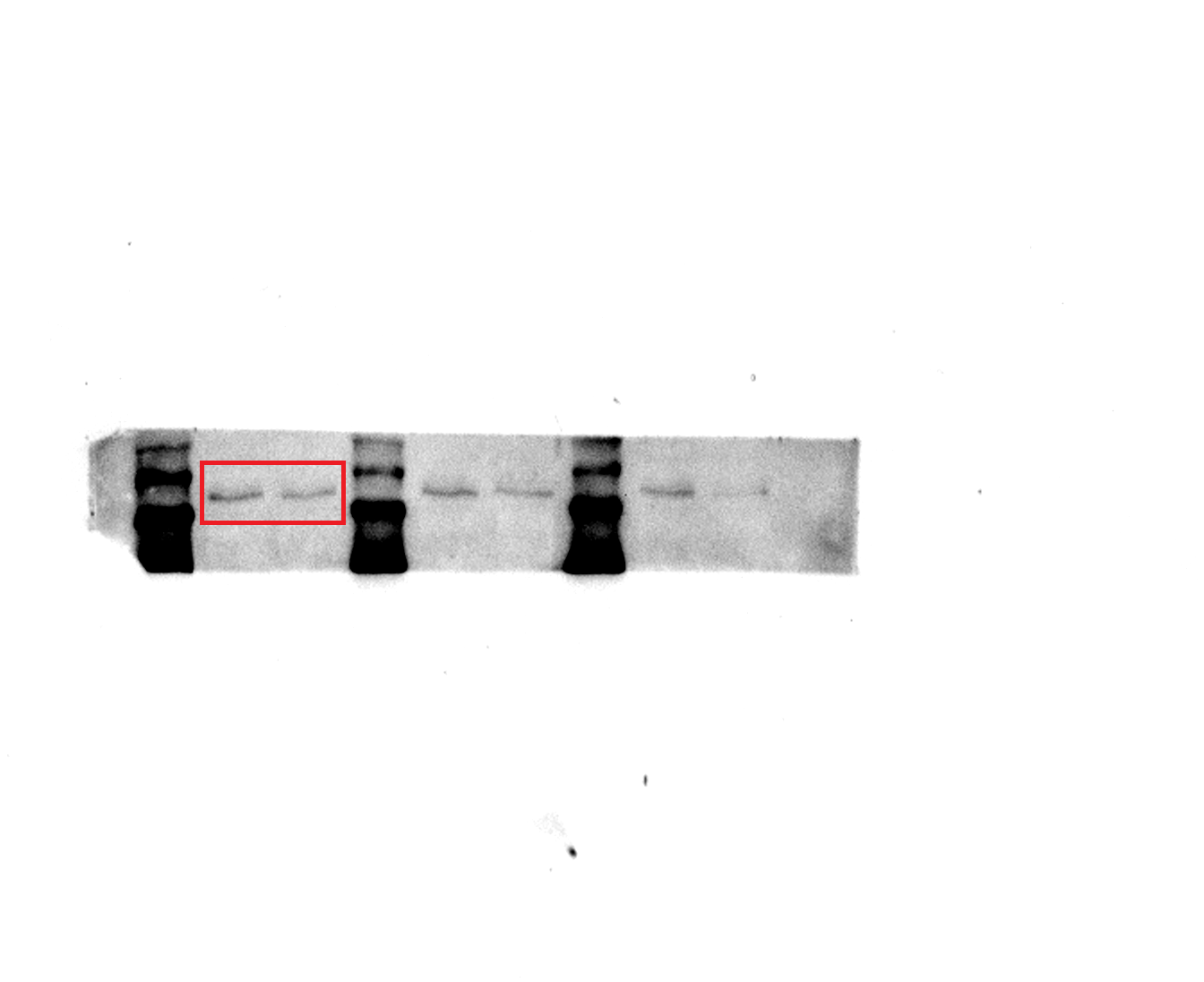

Supplement: Supplemental Information 1 [file peerj-11-15700-s001.zip › raw data 1-western blot/Original Image for Fig 6B/PARP.Tif]

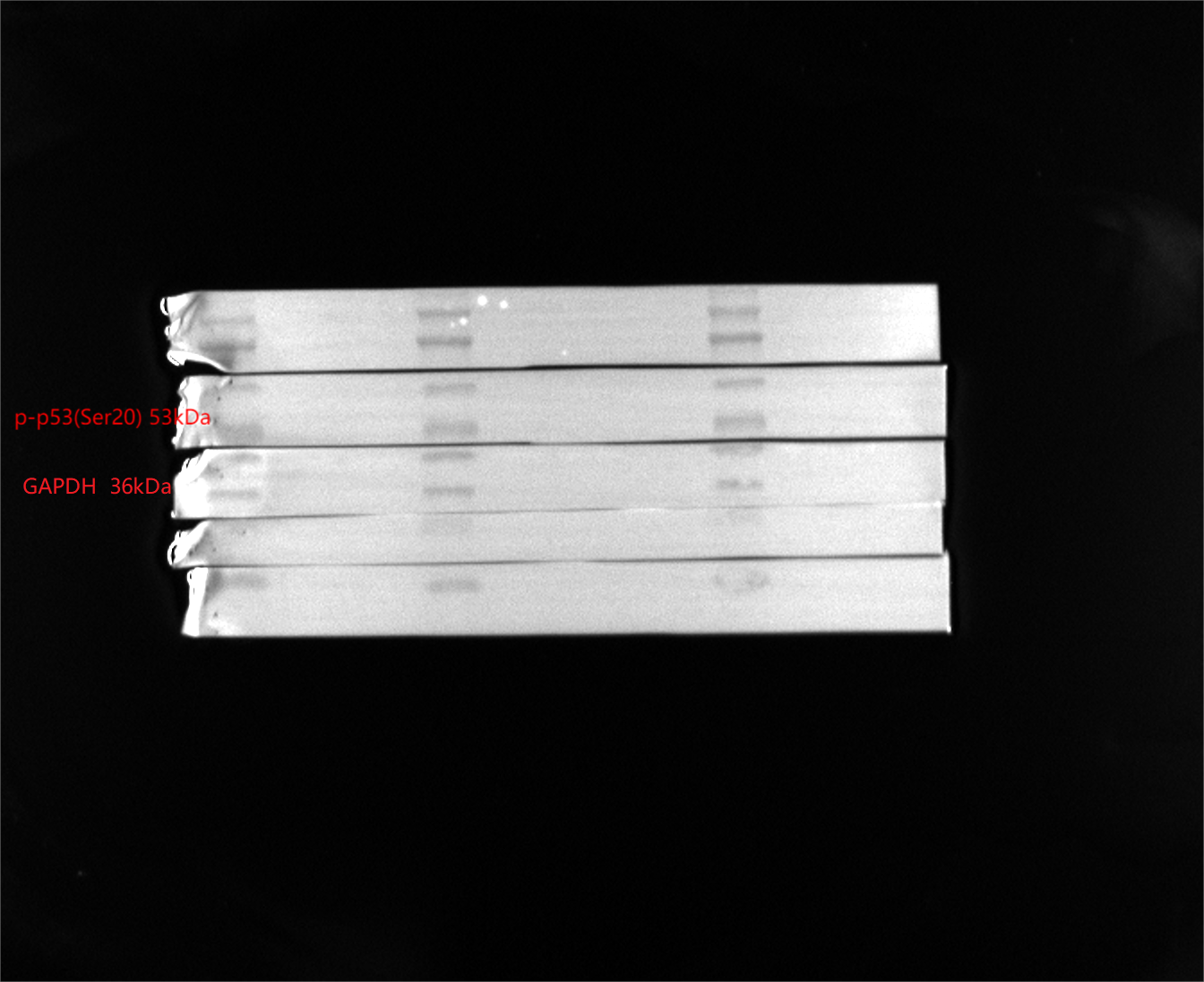

Supplement: Supplemental Information 1 [file peerj-11-15700-s001.zip › raw data 1-western blot/Original Image for Fig 6B/p-p53(Ser20) and GAPDH intact membrane .png]

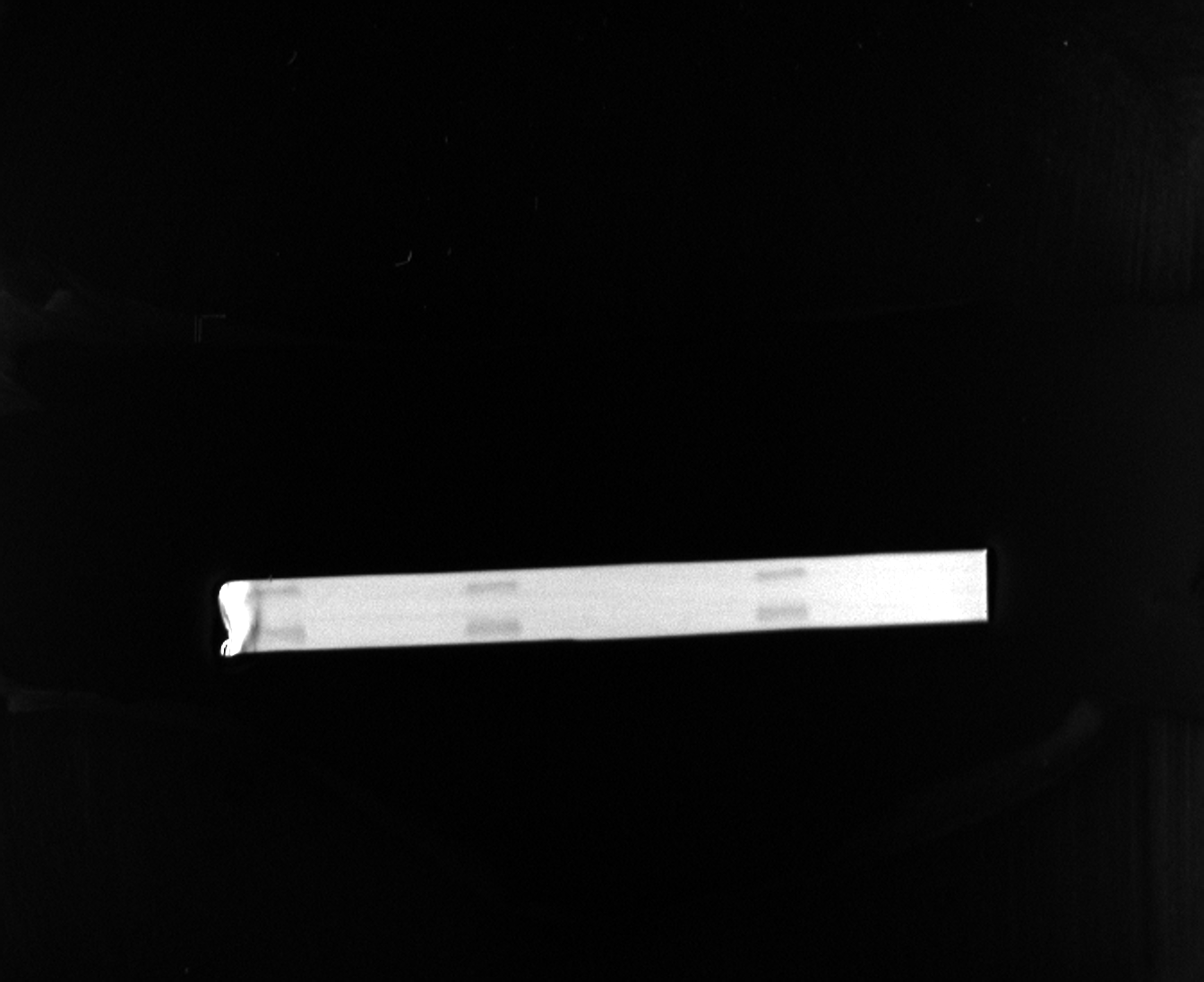

Supplement: Supplemental Information 1 [file peerj-11-15700-s001.zip › raw data 1-western blot/Original Image for Fig 6B/p-p53-original drawing.Tif]

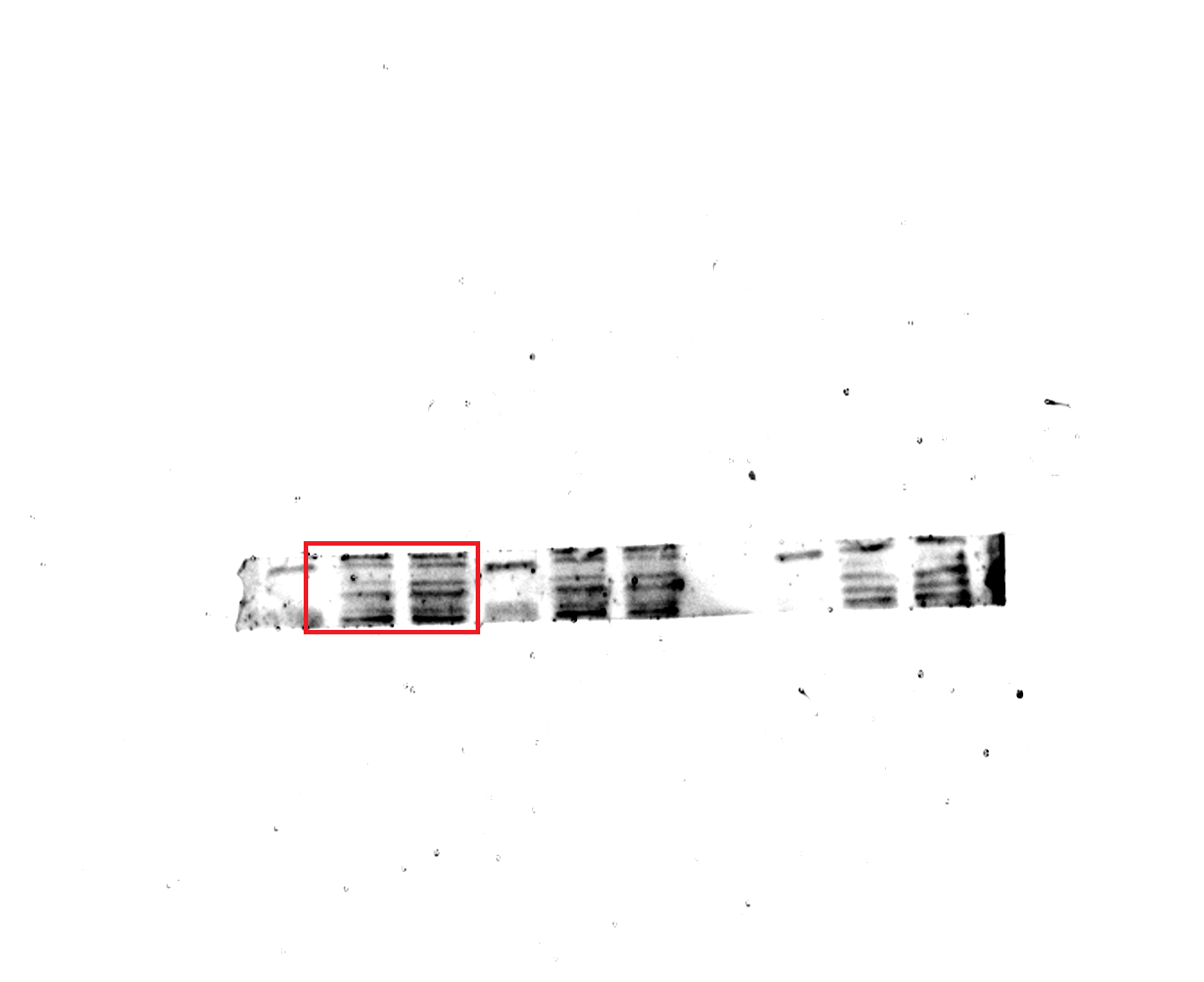

Supplement: Supplemental Information 1 [file peerj-11-15700-s001.zip › raw data 1-western blot/Original Image for Fig 6B/p-p53.Tif]

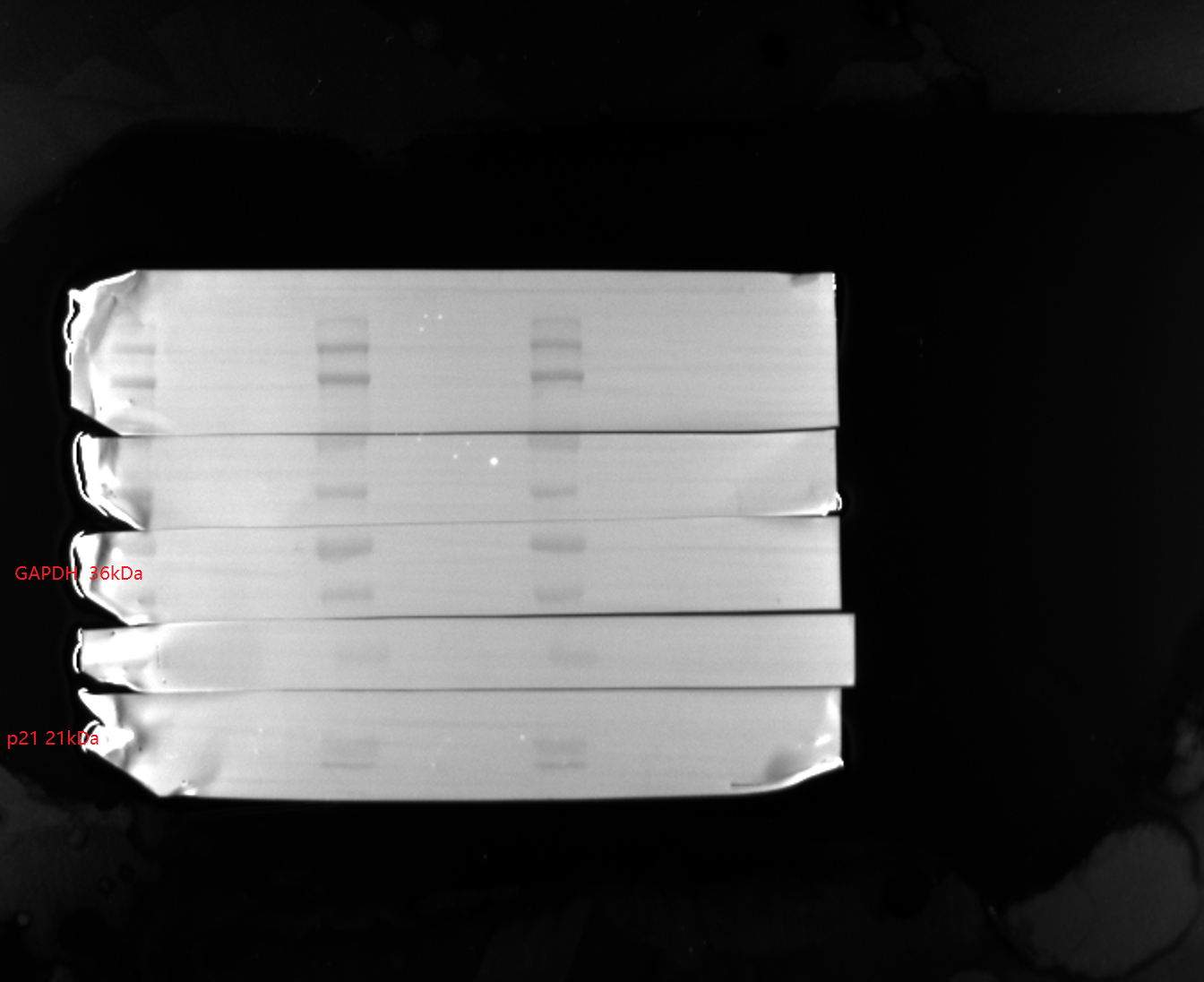

Supplement: Supplemental Information 1 [file peerj-11-15700-s001.zip › raw data 1-western blot/Original Image for Fig 6B/p21 and GAPDH intact membrane .Tif]

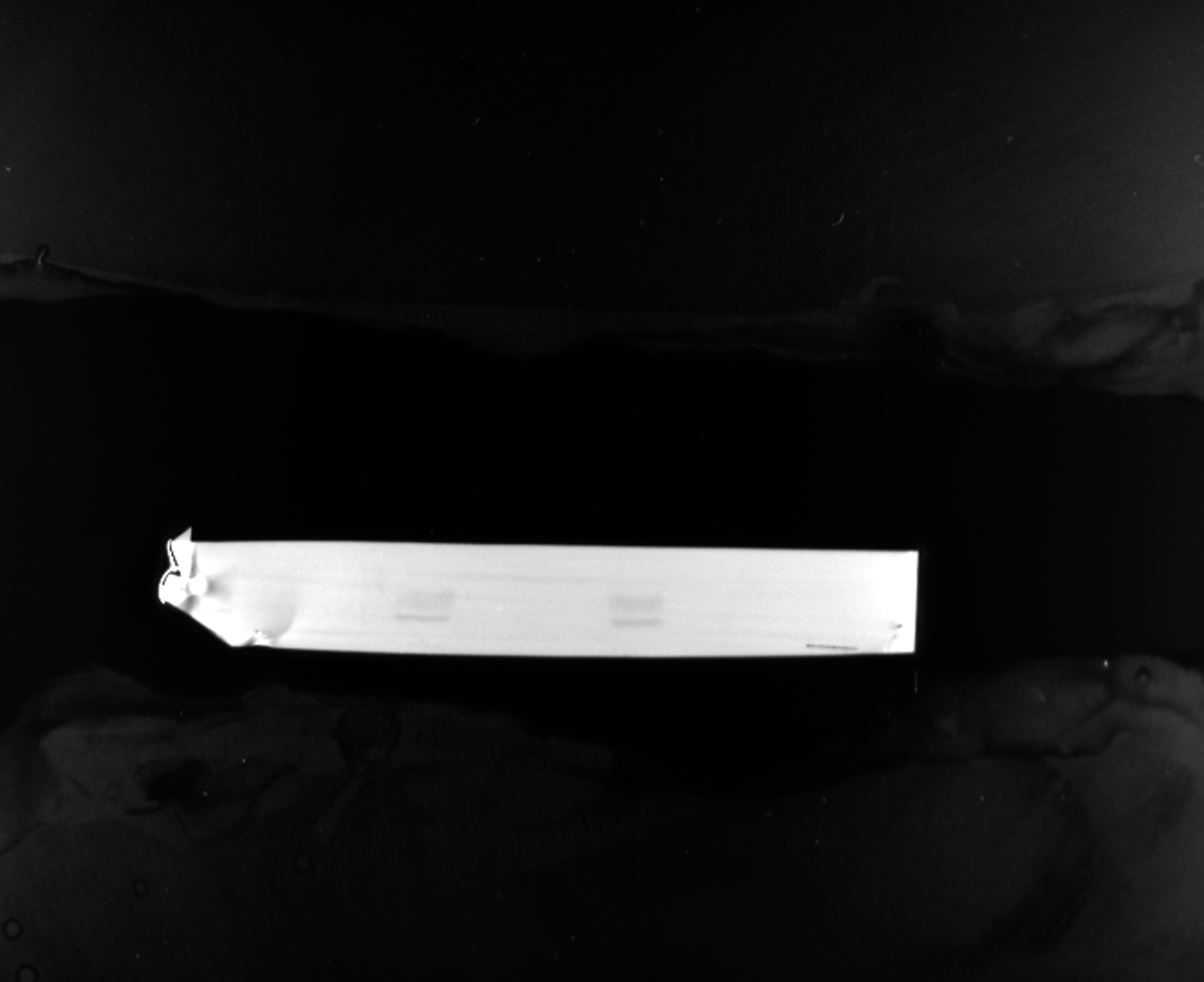

Supplement: Supplemental Information 1 [file peerj-11-15700-s001.zip › raw data 1-western blot/Original Image for Fig 6B/p21-original drawing.Tif]

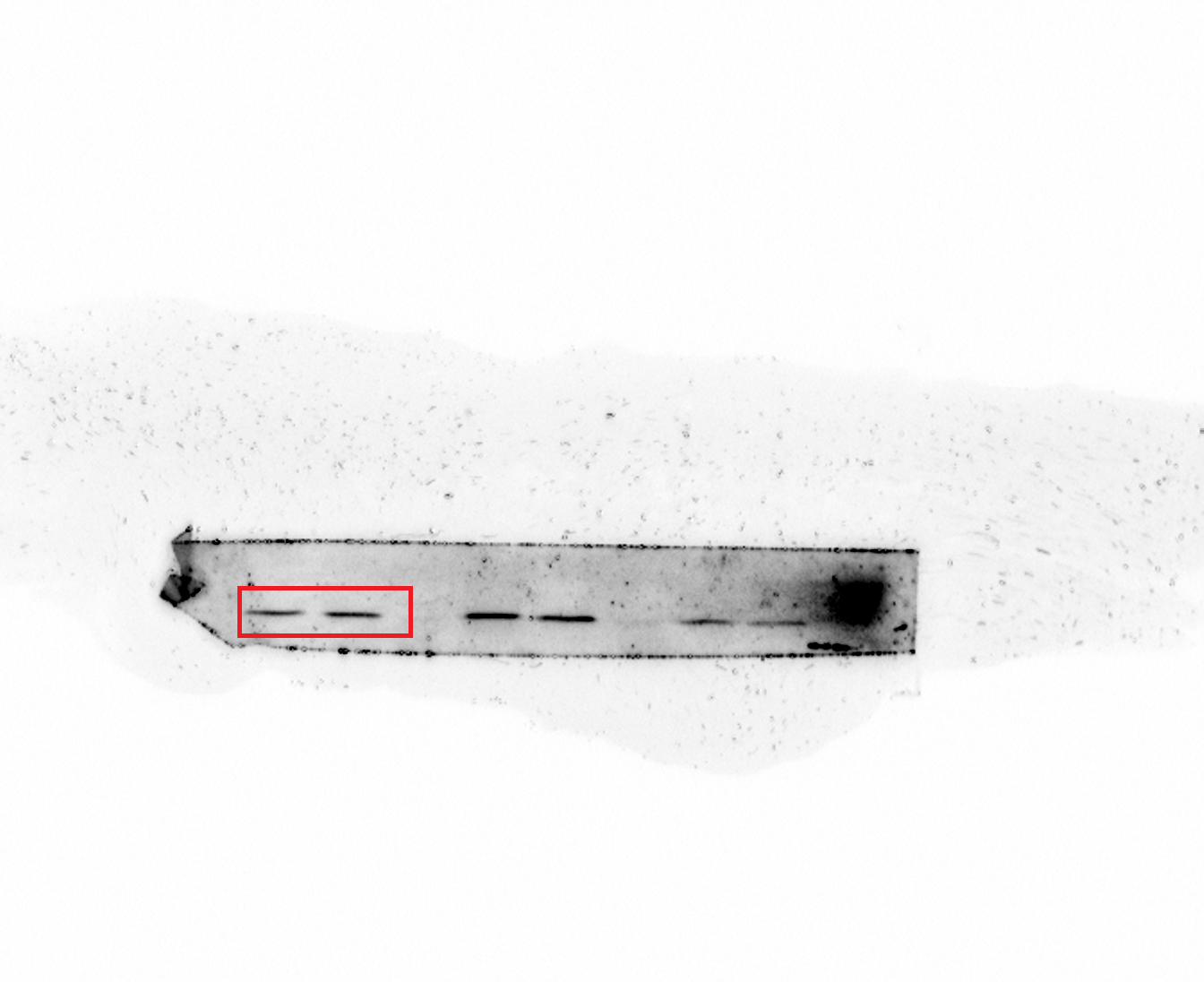

Supplement: Supplemental Information 1 [file peerj-11-15700-s001.zip › raw data 1-western blot/Original Image for Fig 6B/p21.Tif]

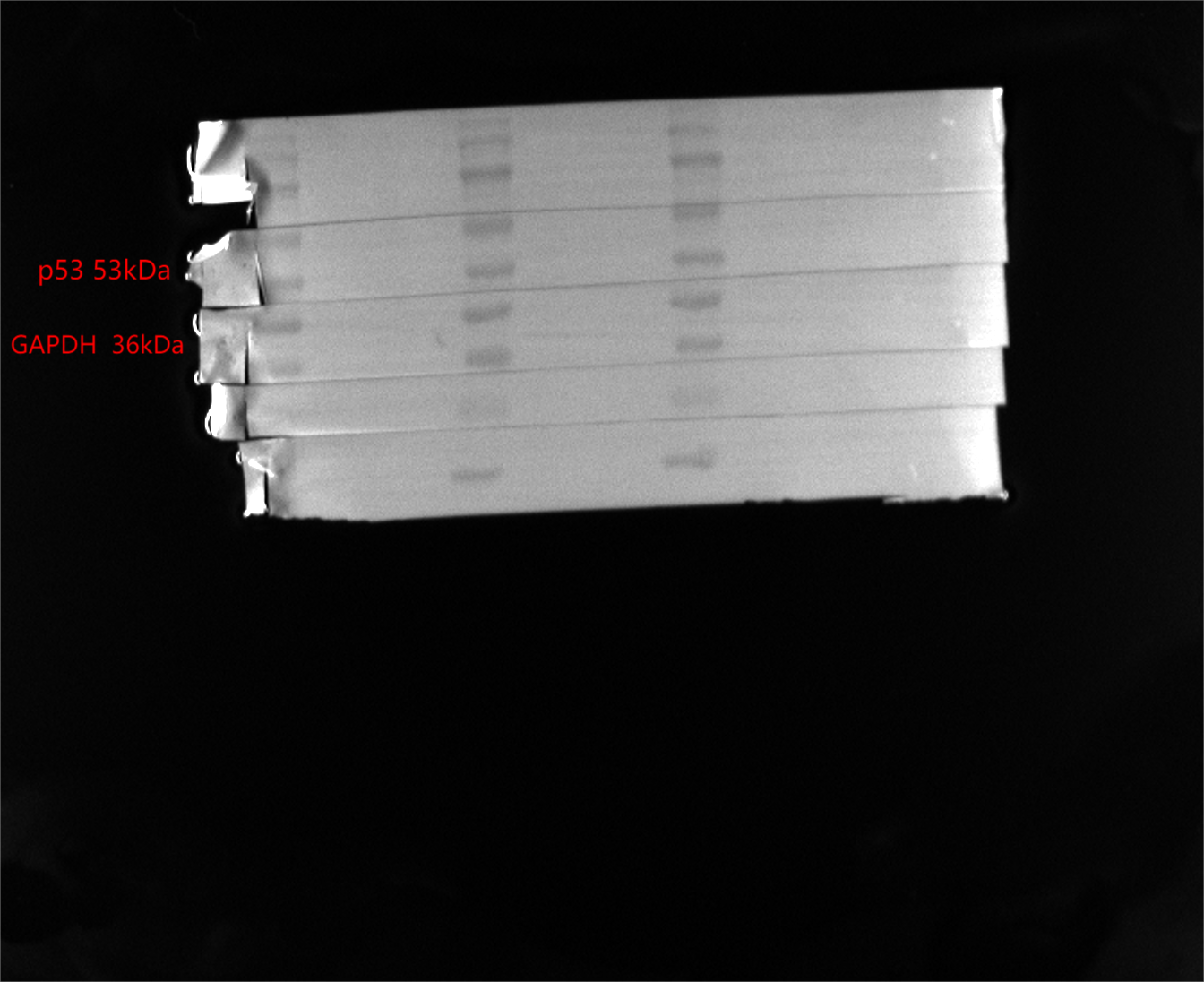

Supplement: Supplemental Information 1 [file peerj-11-15700-s001.zip › raw data 1-western blot/Original Image for Fig 6B/p53 and GAPDH intact membrane .png]

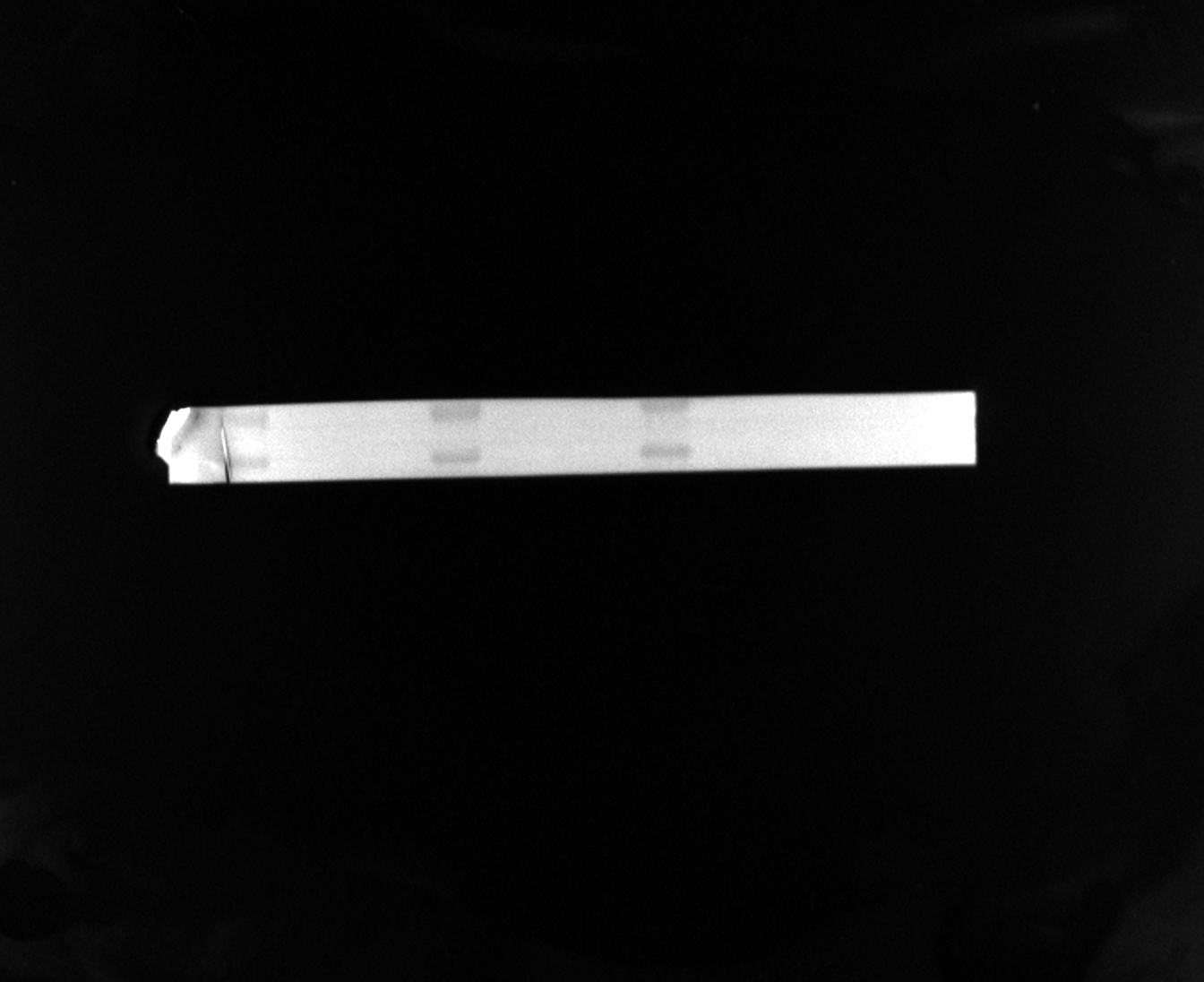

Supplement: Supplemental Information 1 [file peerj-11-15700-s001.zip › raw data 1-western blot/Original Image for Fig 6B/p53-original drawing.Tif]

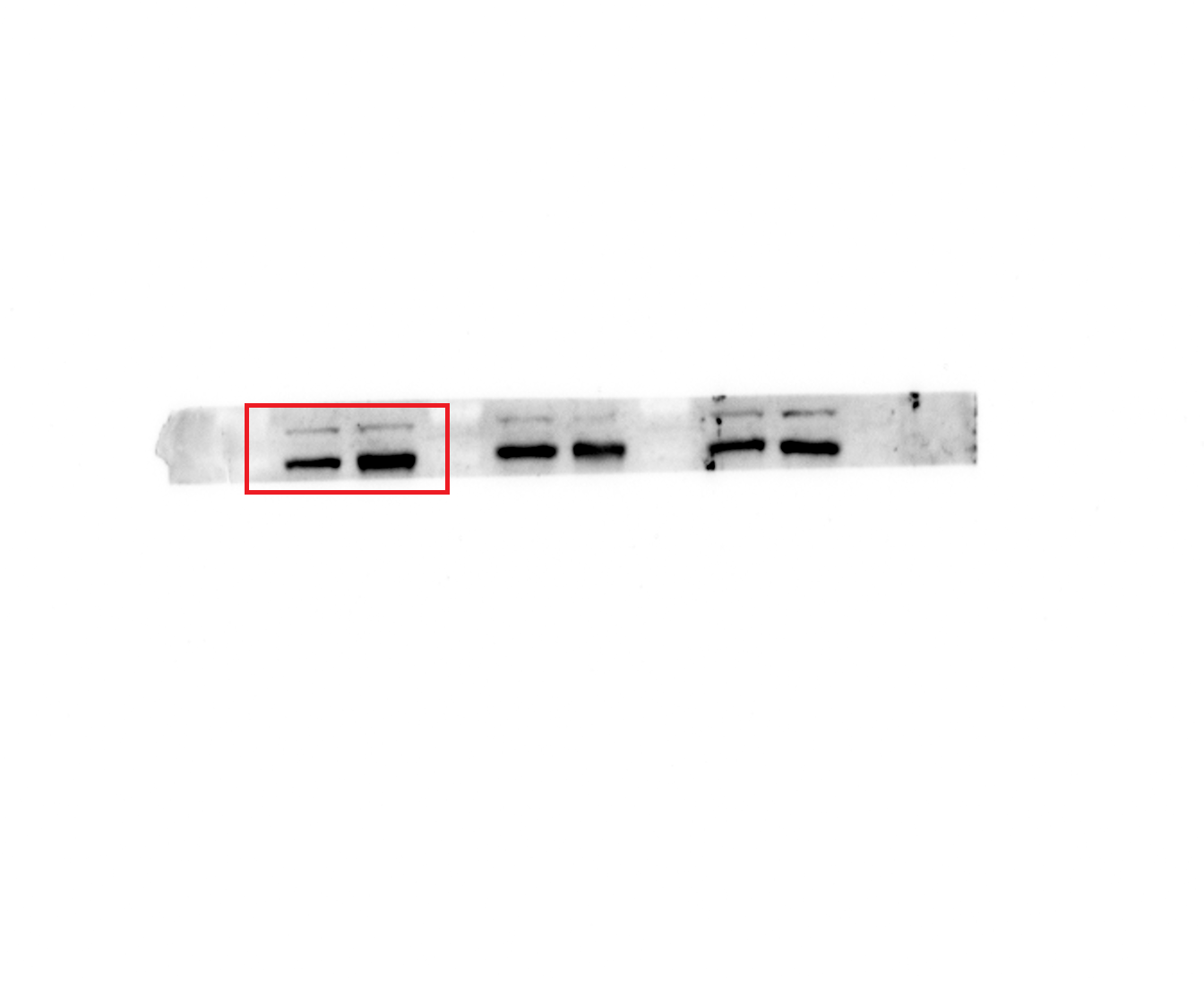

Supplement: Supplemental Information 1 [file peerj-11-15700-s001.zip › raw data 1-western blot/Original Image for Fig 6B/p53.Tif]

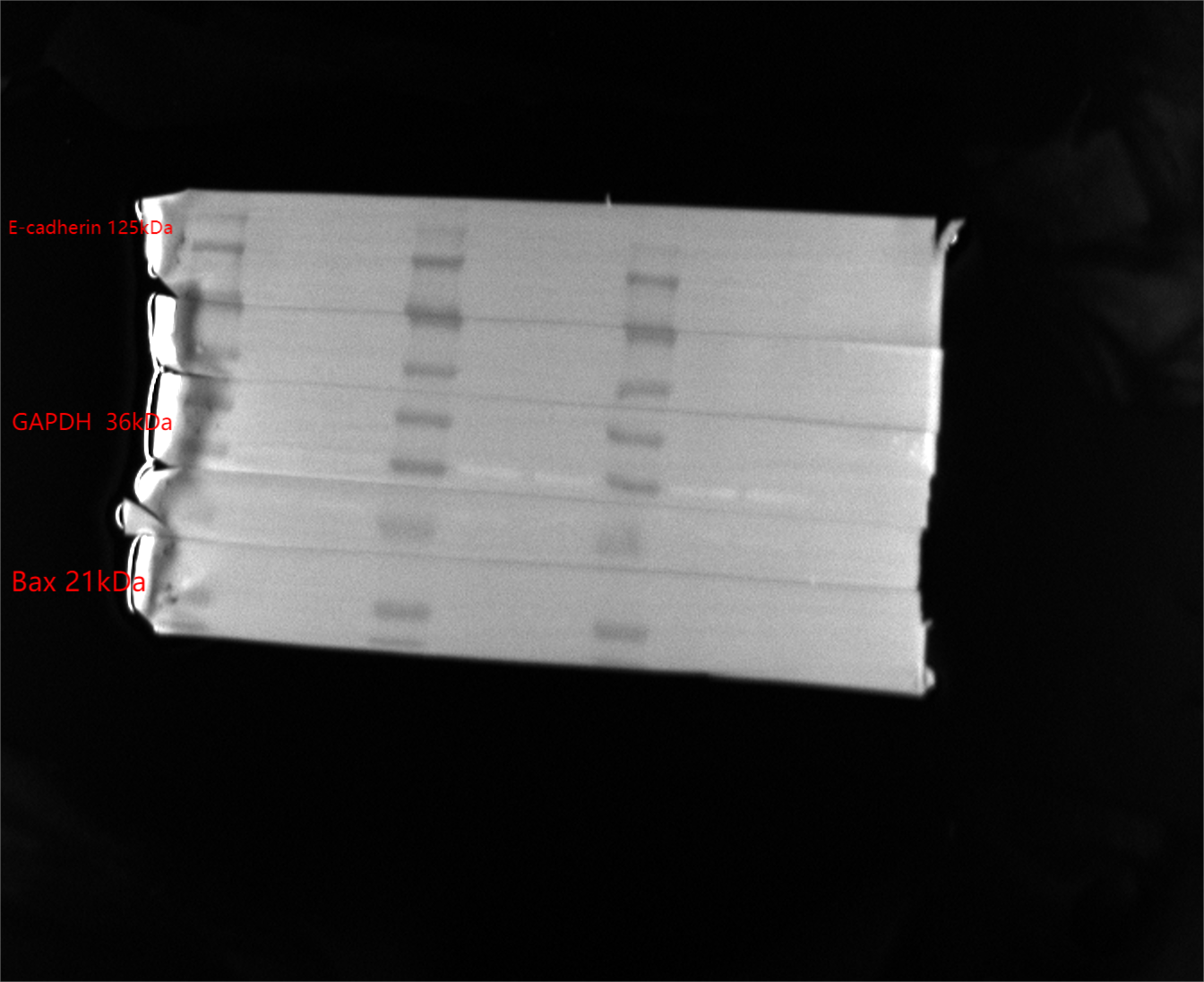

Supplement: Supplemental Information 2 [file peerj-11-15700-s002.zip › raw data 2-western blot/Original Image for Fig 6C/Bax and GAPDH intact membrane .png]

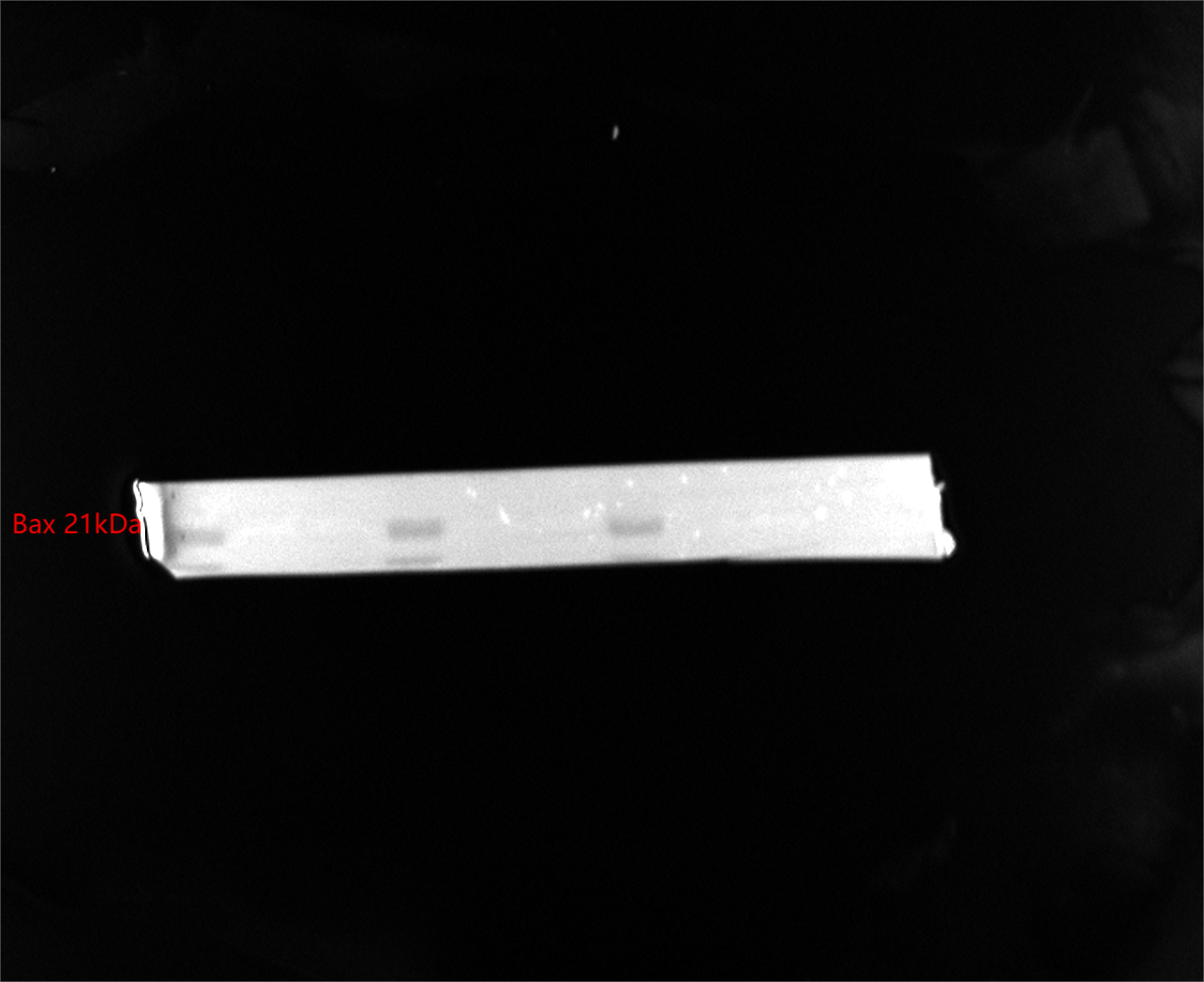

Supplement: Supplemental Information 2 [file peerj-11-15700-s002.zip › raw data 2-western blot/Original Image for Fig 6C/Bax-original drawing.png]

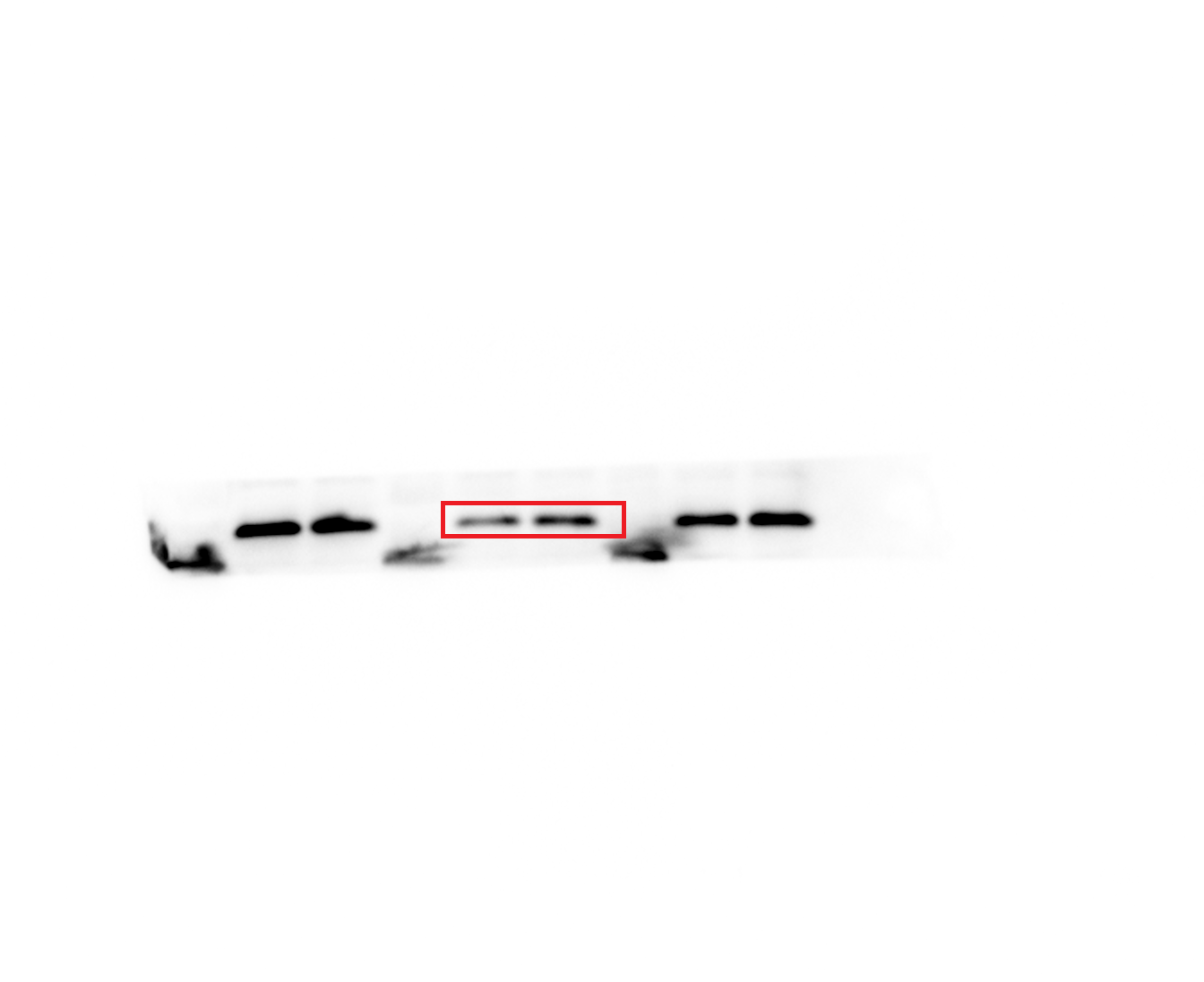

Supplement: Supplemental Information 2 [file peerj-11-15700-s002.zip › raw data 2-western blot/Original Image for Fig 6C/Bax.Tif]

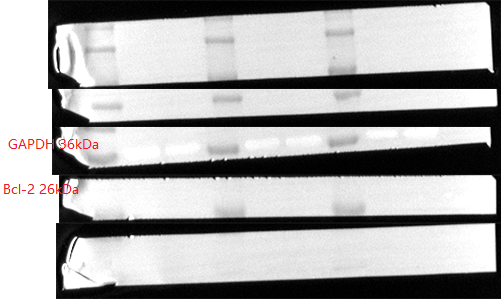

Supplement: Supplemental Information 2 [file peerj-11-15700-s002.zip › raw data 2-western blot/Original Image for Fig 6C/Bcl-2 and GAPDH intact membrane .tif]

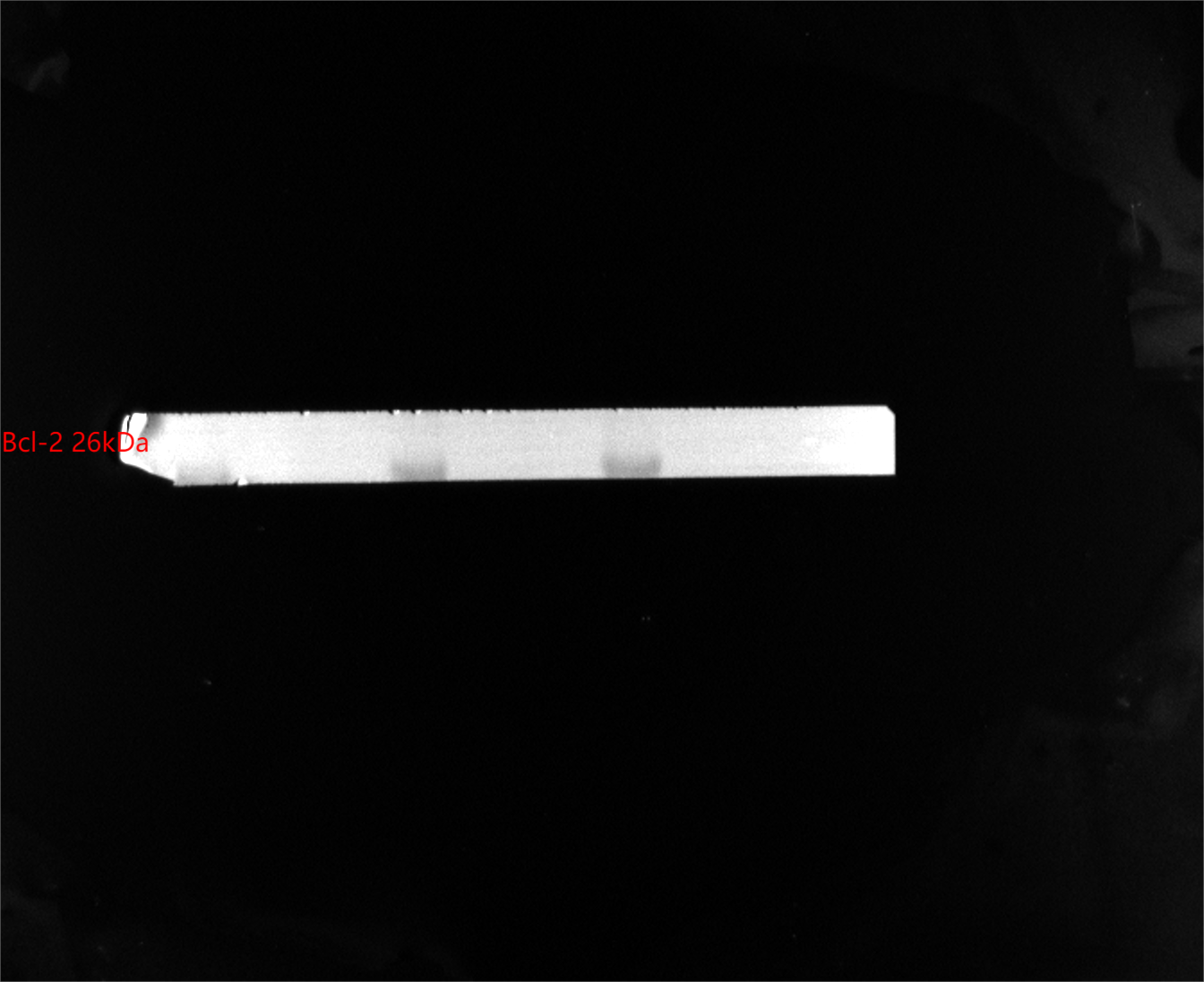

Supplement: Supplemental Information 2 [file peerj-11-15700-s002.zip › raw data 2-western blot/Original Image for Fig 6C/Bcl-2-original drawing.png]

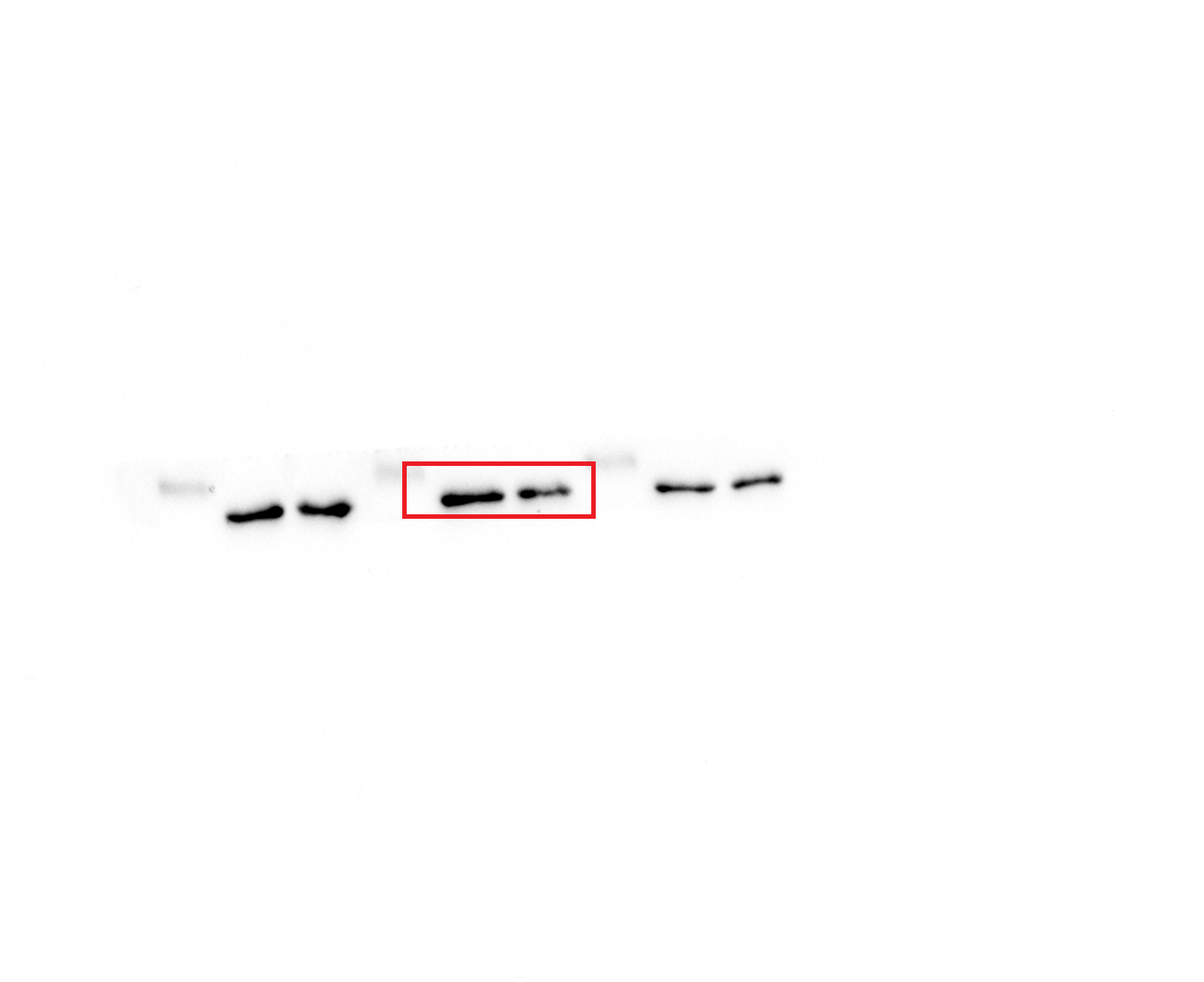

Supplement: Supplemental Information 2 [file peerj-11-15700-s002.zip › raw data 2-western blot/Original Image for Fig 6C/Bcl-2.Tif]

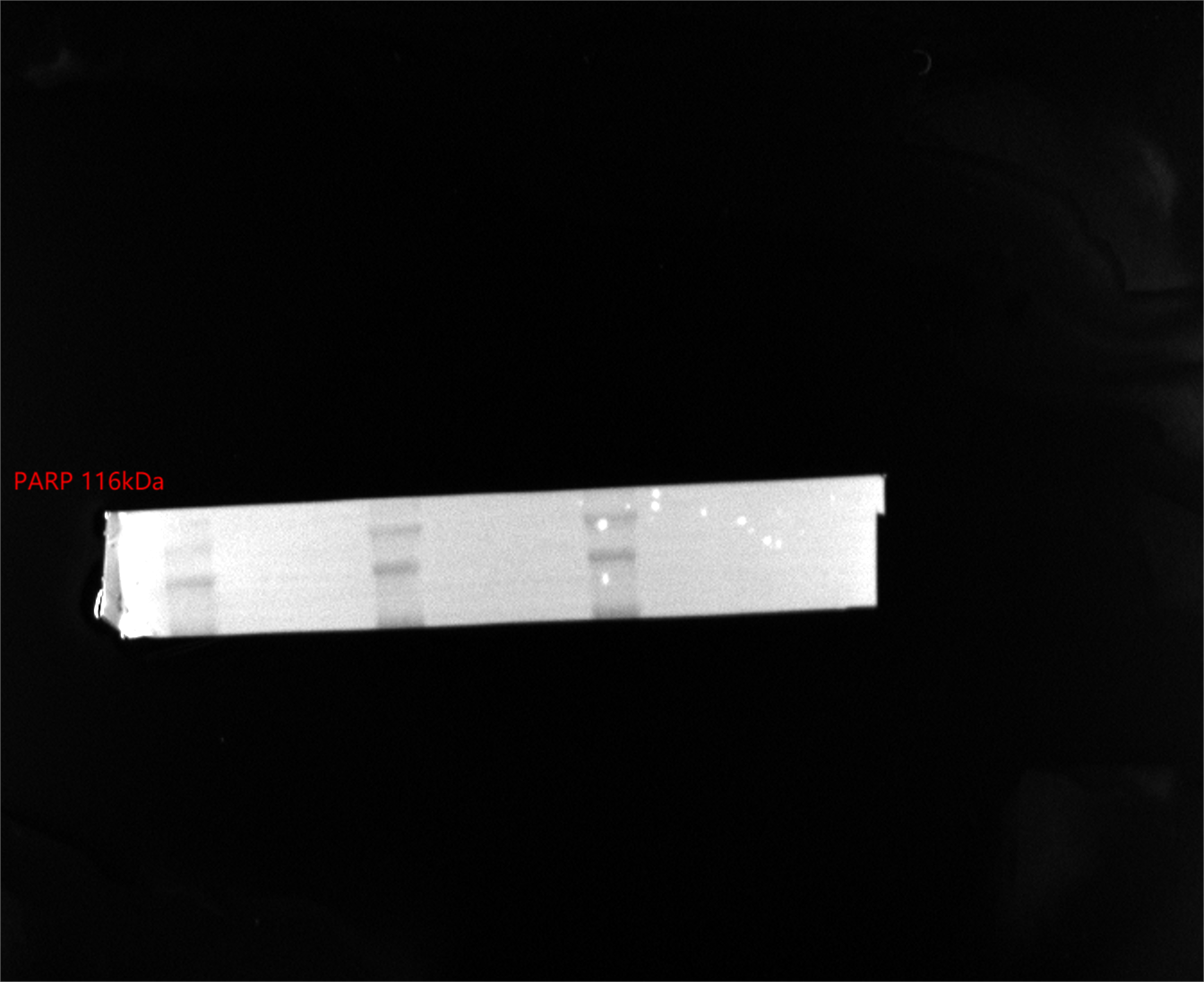

Supplement: Supplemental Information 2 [file peerj-11-15700-s002.zip › raw data 2-western blot/Original Image for Fig 6C/PARP-original drawing.png]

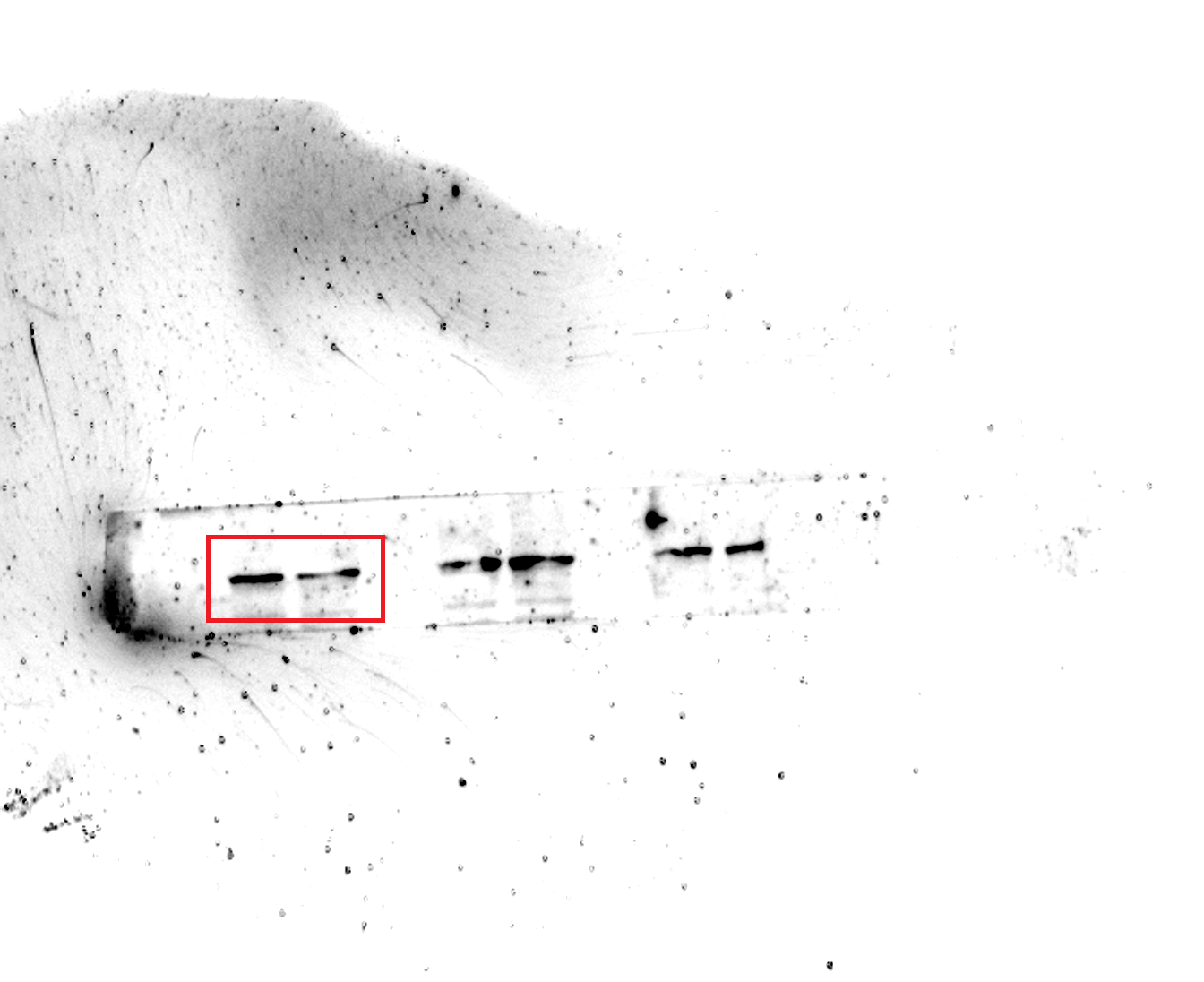

Supplement: Supplemental Information 2 [file peerj-11-15700-s002.zip › raw data 2-western blot/Original Image for Fig 6C/PARP.Tif]

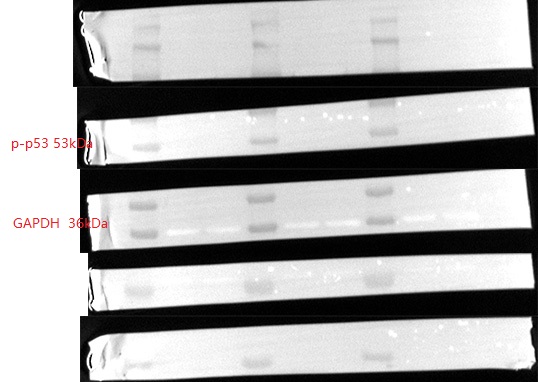

Supplement: Supplemental Information 2 [file peerj-11-15700-s002.zip › raw data 2-western blot/Original Image for Fig 6C/p-p53 and GAPDH intact membrane .jpg]

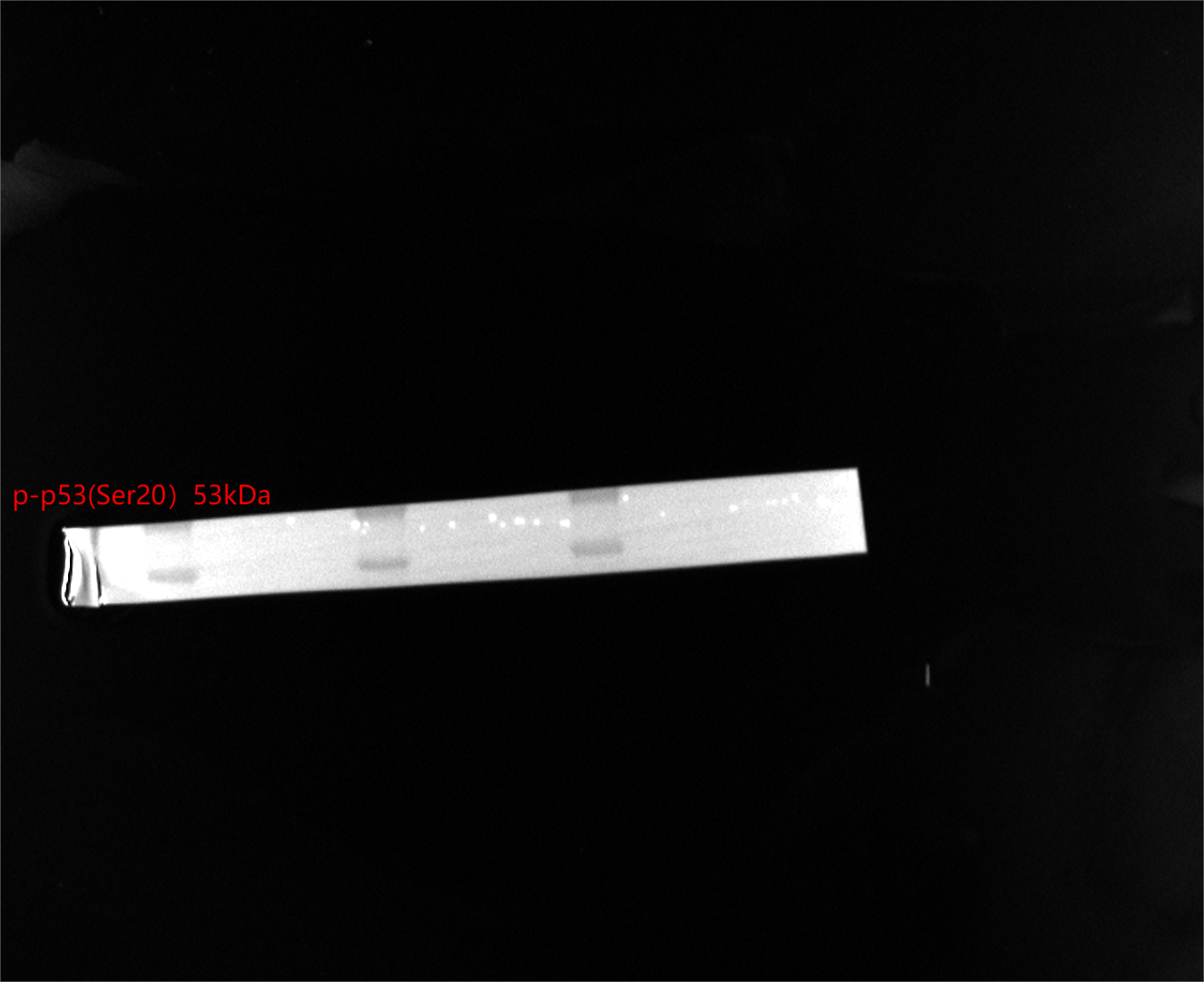

Supplement: Supplemental Information 2 [file peerj-11-15700-s002.zip › raw data 2-western blot/Original Image for Fig 6C/p-p53(Ser20)-original drawing.png]

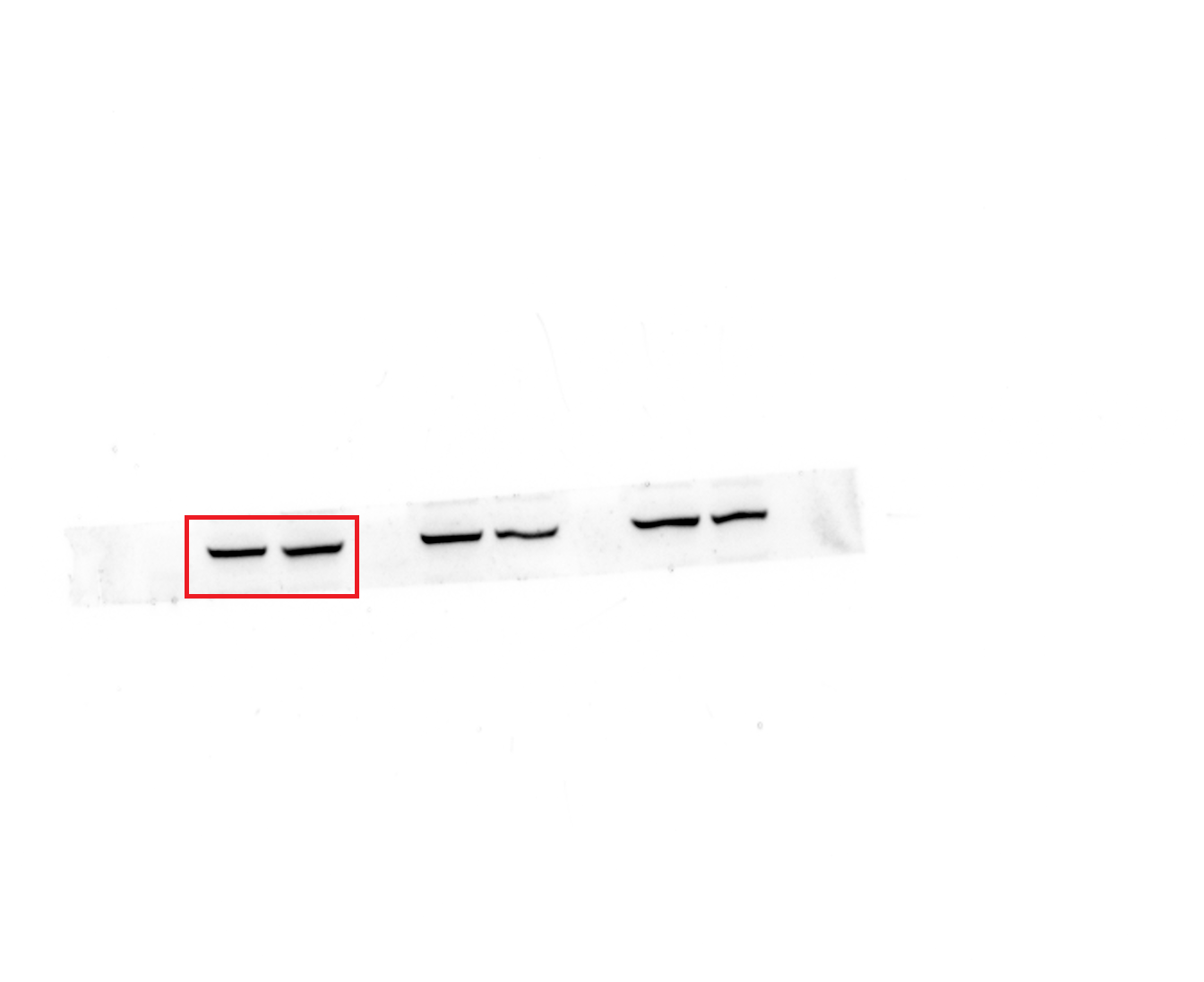

Supplement: Supplemental Information 2 [file peerj-11-15700-s002.zip › raw data 2-western blot/Original Image for Fig 6C/p-p53(Ser20).Tif]

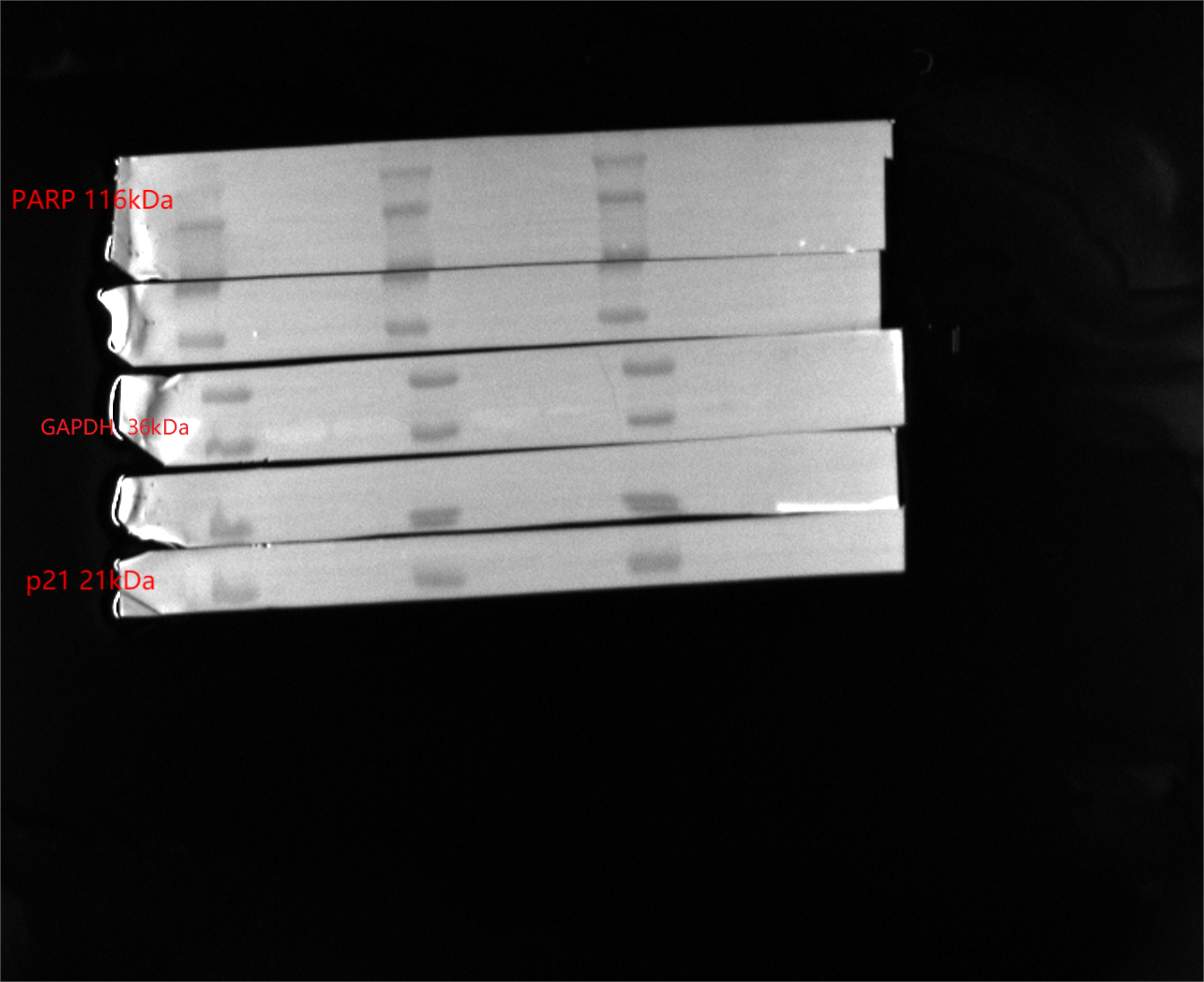

Supplement: Supplemental Information 2 [file peerj-11-15700-s002.zip › raw data 2-western blot/Original Image for Fig 6C/p21, PARP and GAPDH intact membrane.png]

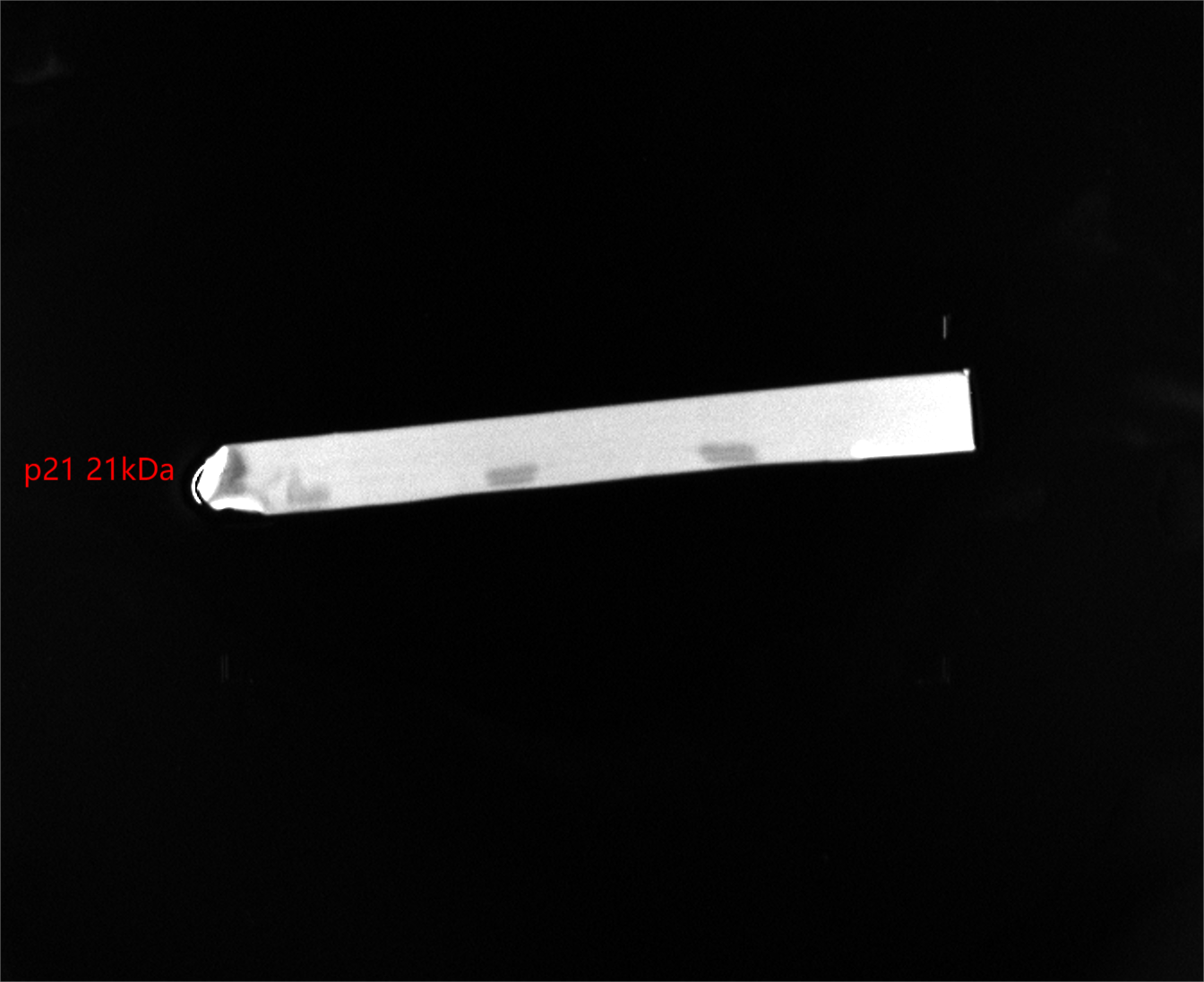

Supplement: Supplemental Information 2 [file peerj-11-15700-s002.zip › raw data 2-western blot/Original Image for Fig 6C/p21-original drawing.png]

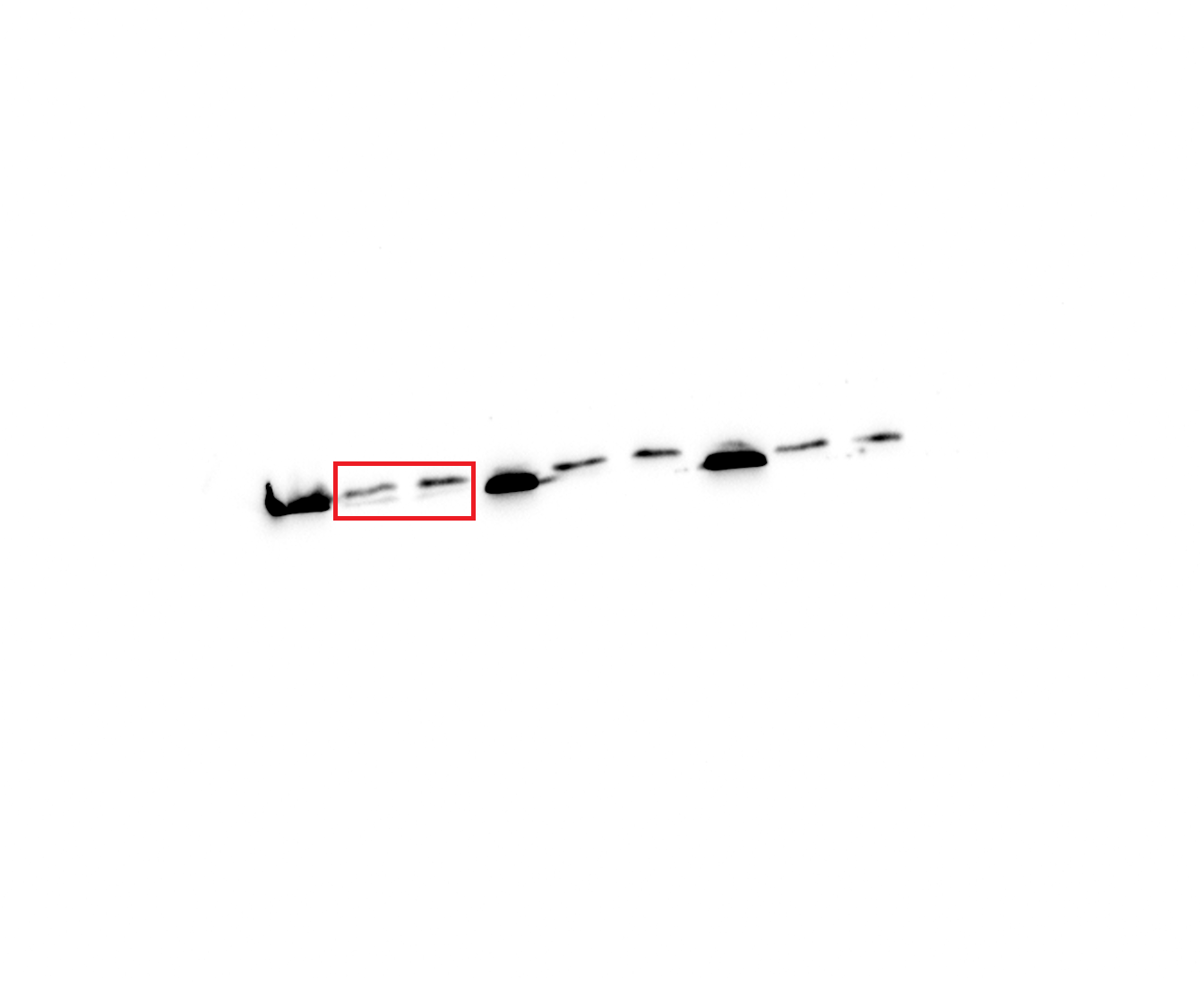

Supplement: Supplemental Information 2 [file peerj-11-15700-s002.zip › raw data 2-western blot/Original Image for Fig 6C/p21.Tif]

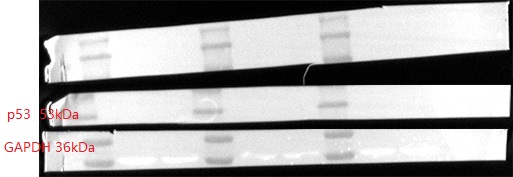

Supplement: Supplemental Information 2 [file peerj-11-15700-s002.zip › raw data 2-western blot/Original Image for Fig 6C/p53 and GAPDH intact membrane .jpg]

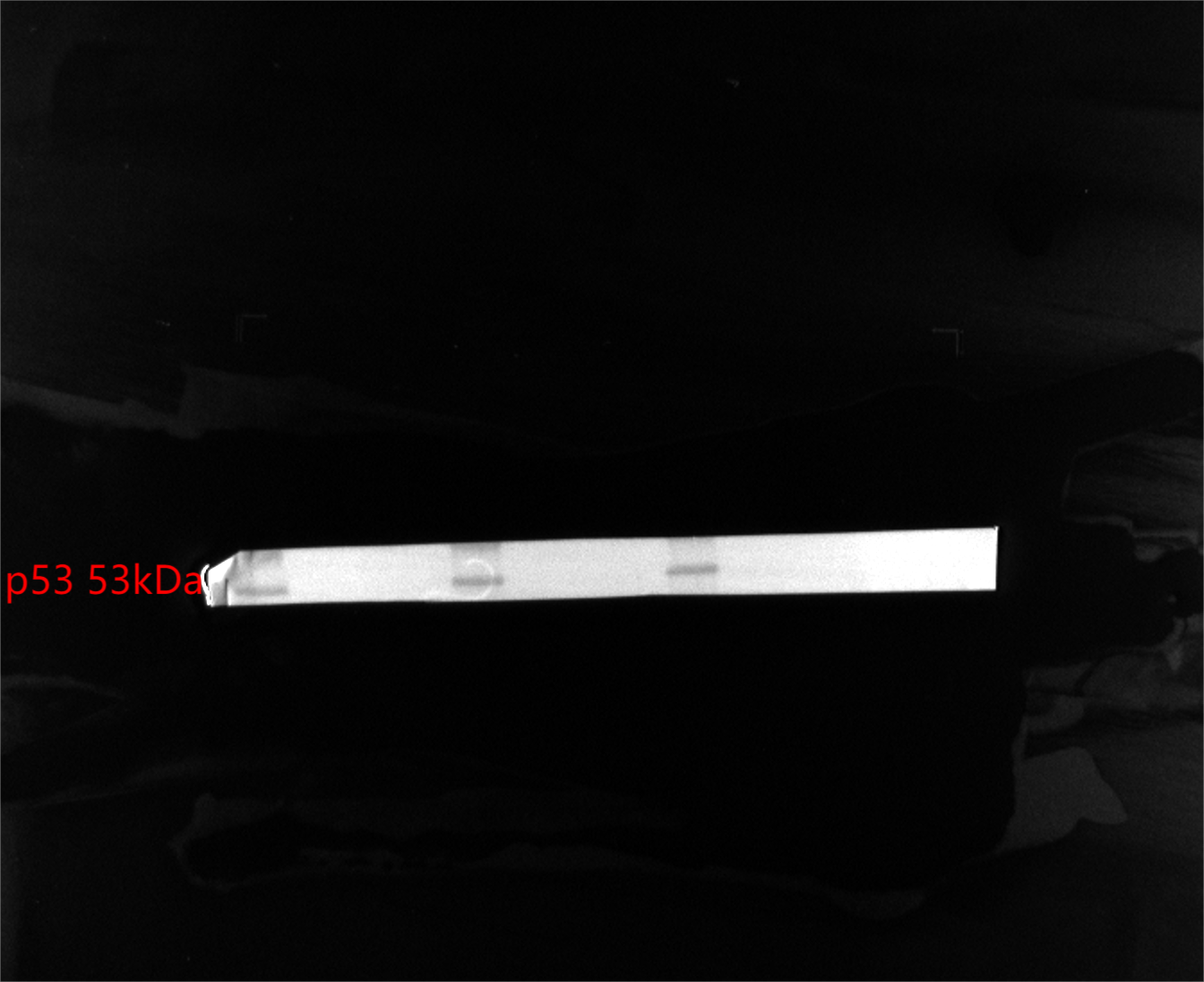

Supplement: Supplemental Information 2 [file peerj-11-15700-s002.zip › raw data 2-western blot/Original Image for Fig 6C/p53-original drawing.png]

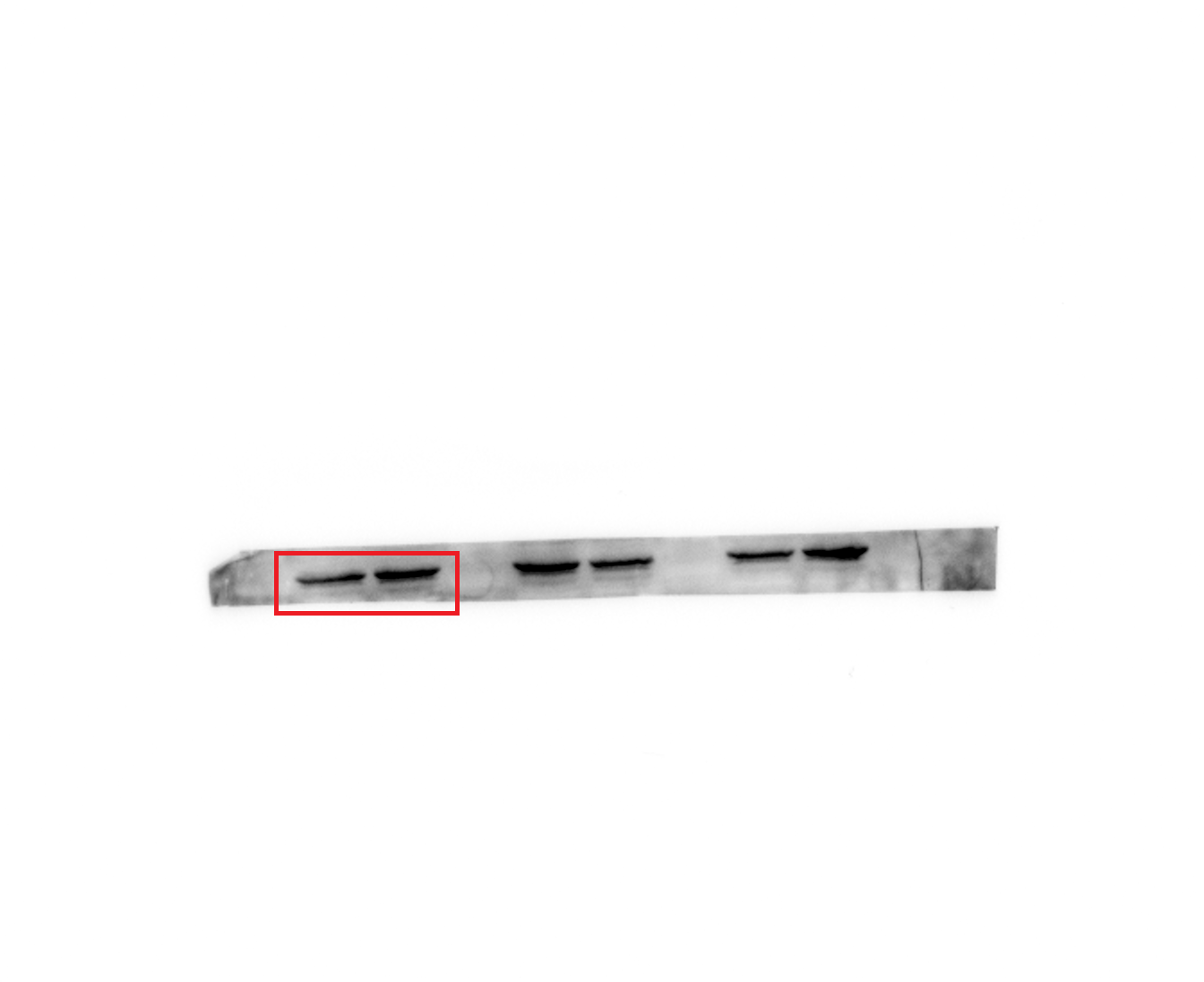

Supplement: Supplemental Information 2 [file peerj-11-15700-s002.zip › raw data 2-western blot/Original Image for Fig 6C/p53.Tif]

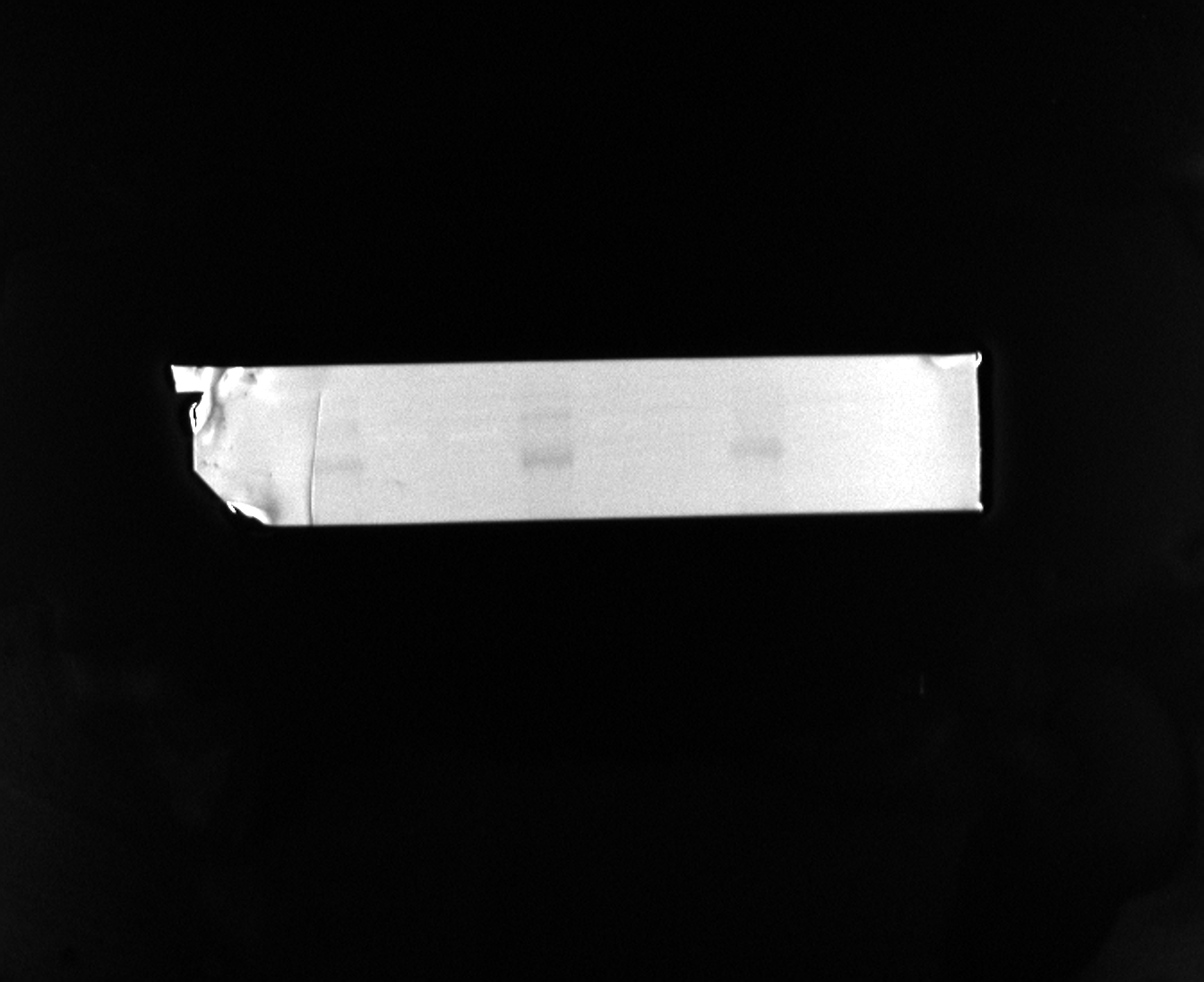

Supplement: Supplemental Information 2 [file peerj-11-15700-s002.zip › raw data 2-western blot/Original Image for Fig 9A/E-cadherin original drawing.Tif]

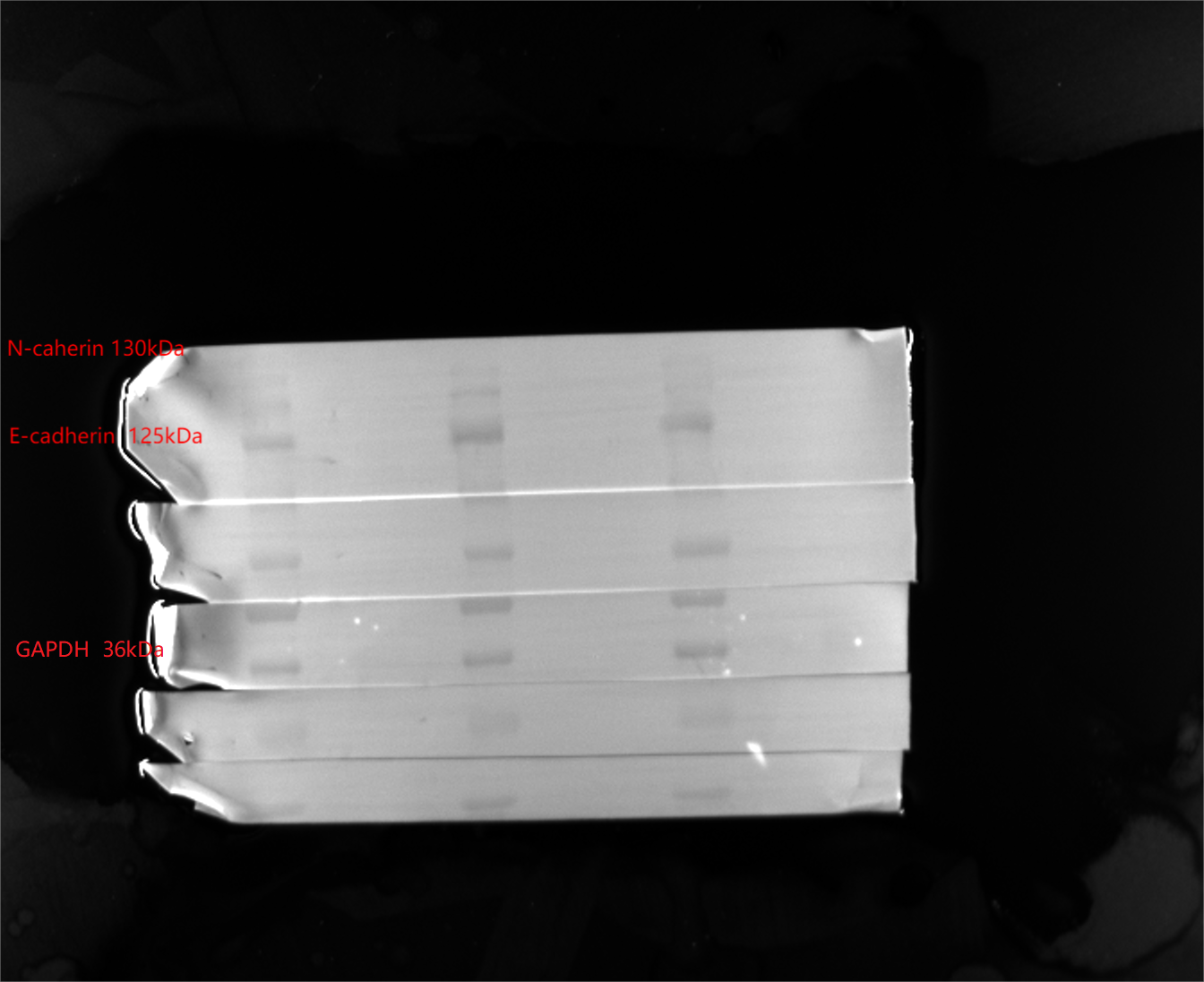

Supplement: Supplemental Information 2 [file peerj-11-15700-s002.zip › raw data 2-western blot/Original Image for Fig 9A/E-cadherin, N-cadherin and GAPDH intact membrane .png]

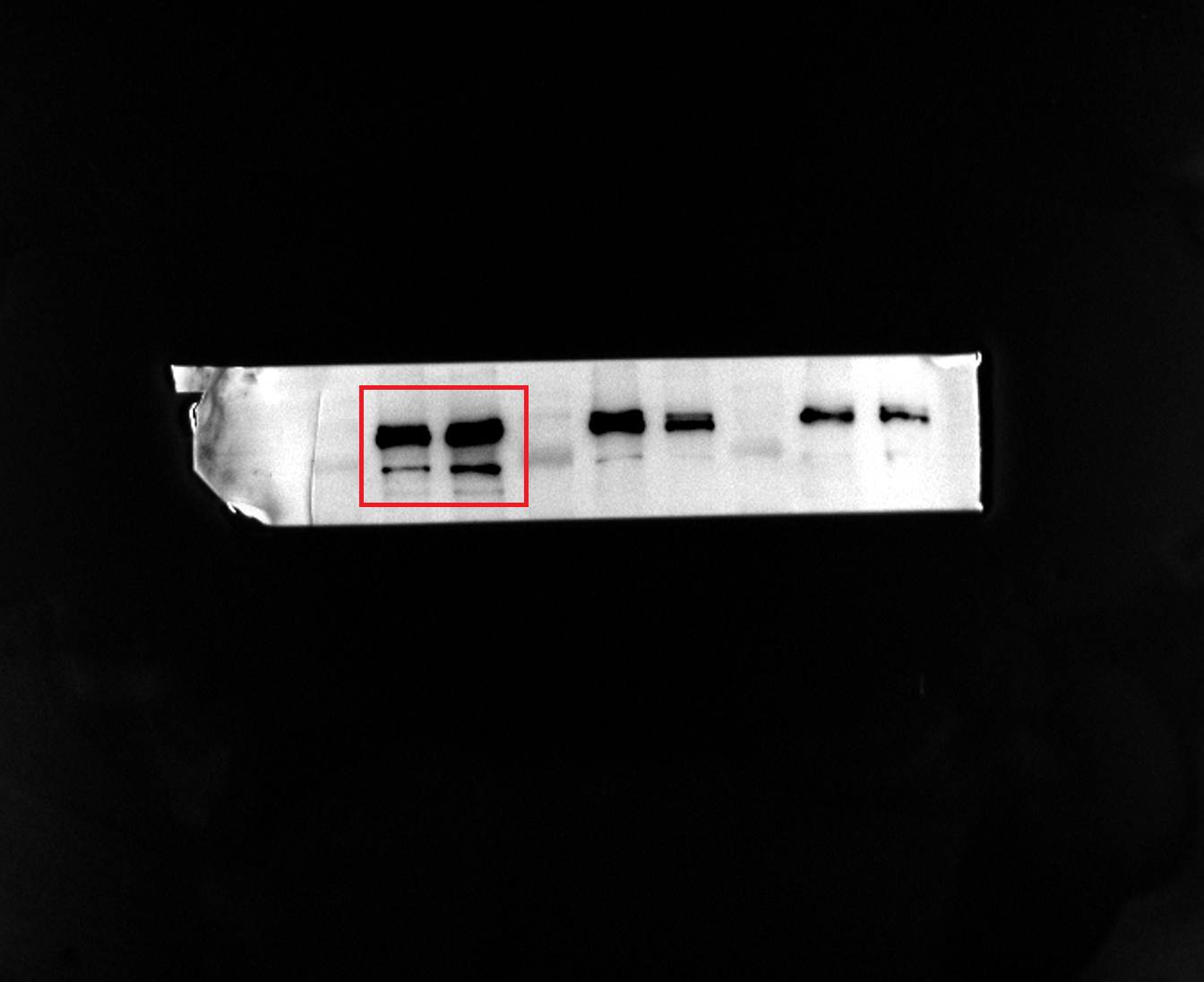

Supplement: Supplemental Information 2 [file peerj-11-15700-s002.zip › raw data 2-western blot/Original Image for Fig 9A/E-cadherin.Tif]

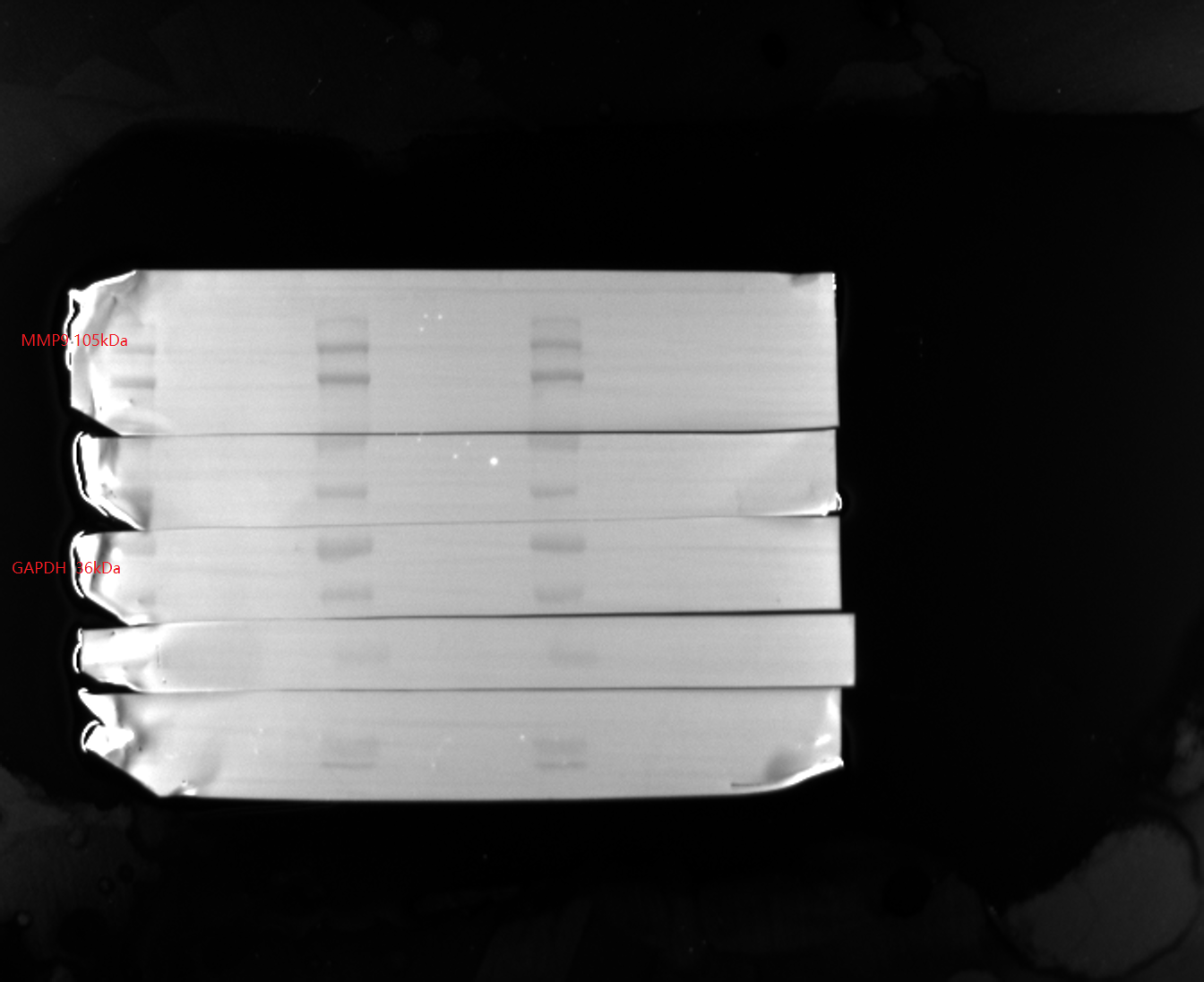

Supplement: Supplemental Information 2 [file peerj-11-15700-s002.zip › raw data 2-western blot/Original Image for Fig 9A/MMP9 and GAPDH intact membrane .Tif]

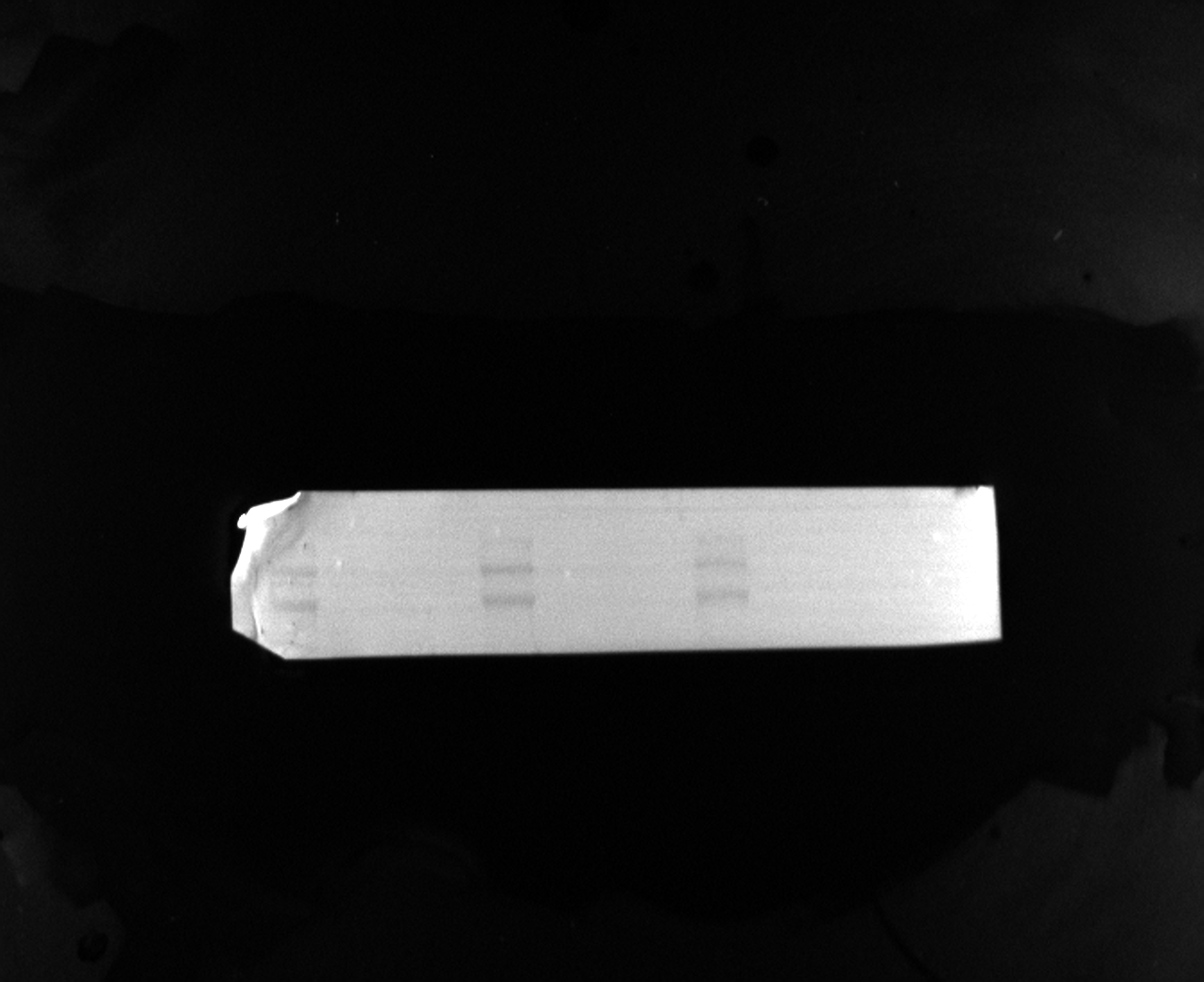

Supplement: Supplemental Information 2 [file peerj-11-15700-s002.zip › raw data 2-western blot/Original Image for Fig 9A/MMP9 original drawing.Tif]

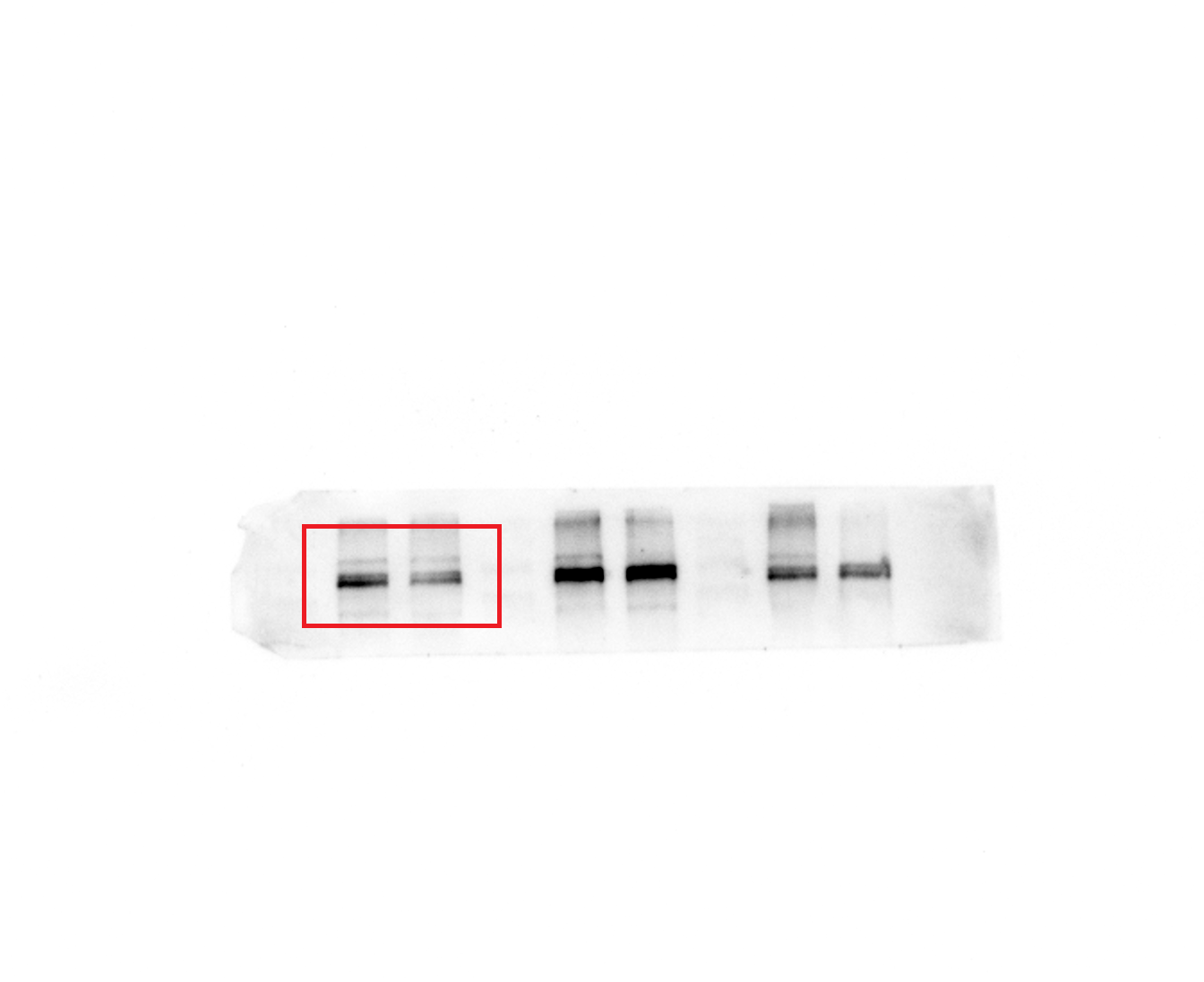

Supplement: Supplemental Information 2 [file peerj-11-15700-s002.zip › raw data 2-western blot/Original Image for Fig 9A/MMP9.Tif]

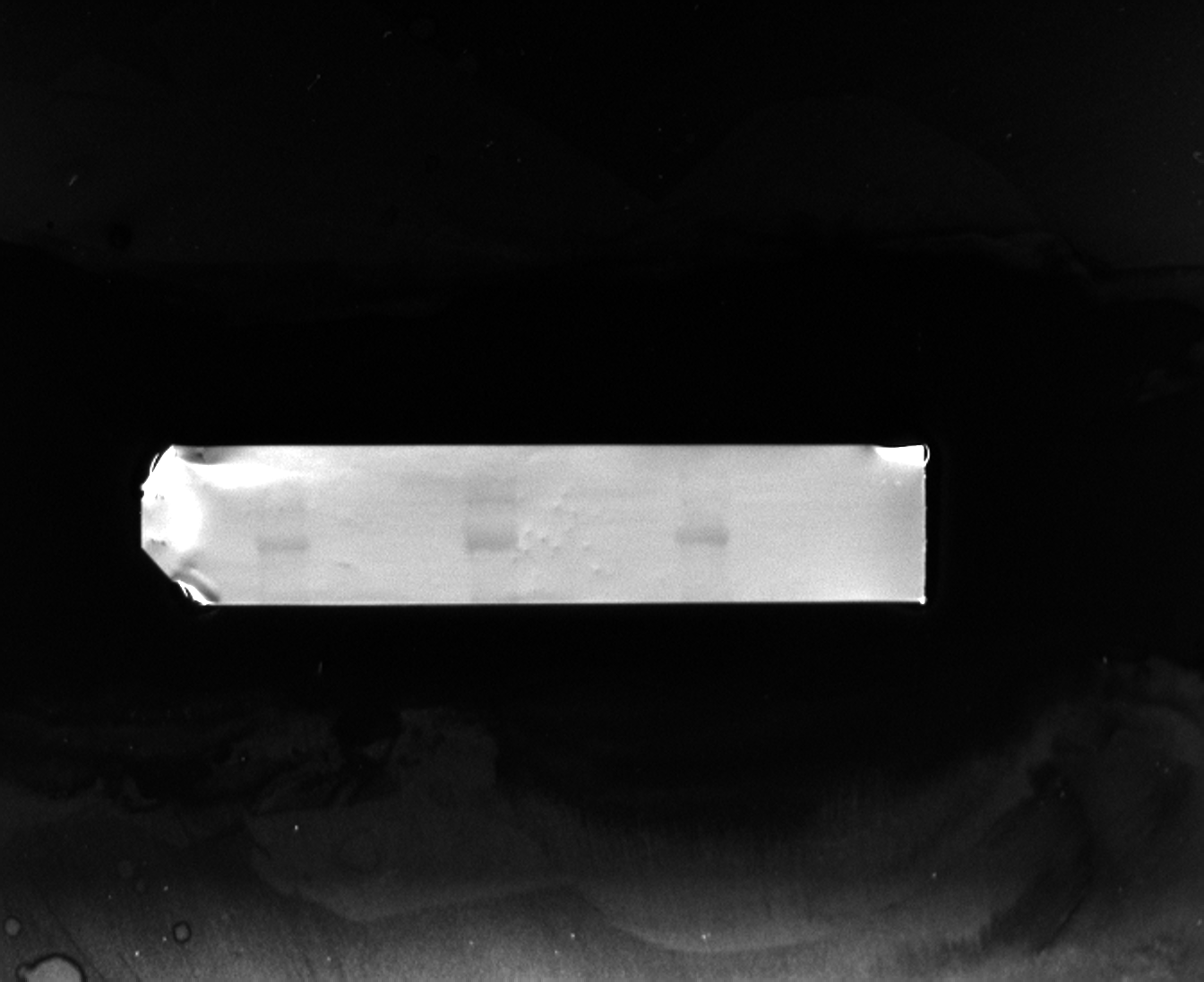

Supplement: Supplemental Information 2 [file peerj-11-15700-s002.zip › raw data 2-western blot/Original Image for Fig 9A/N-cadherin original drawing.Tif]

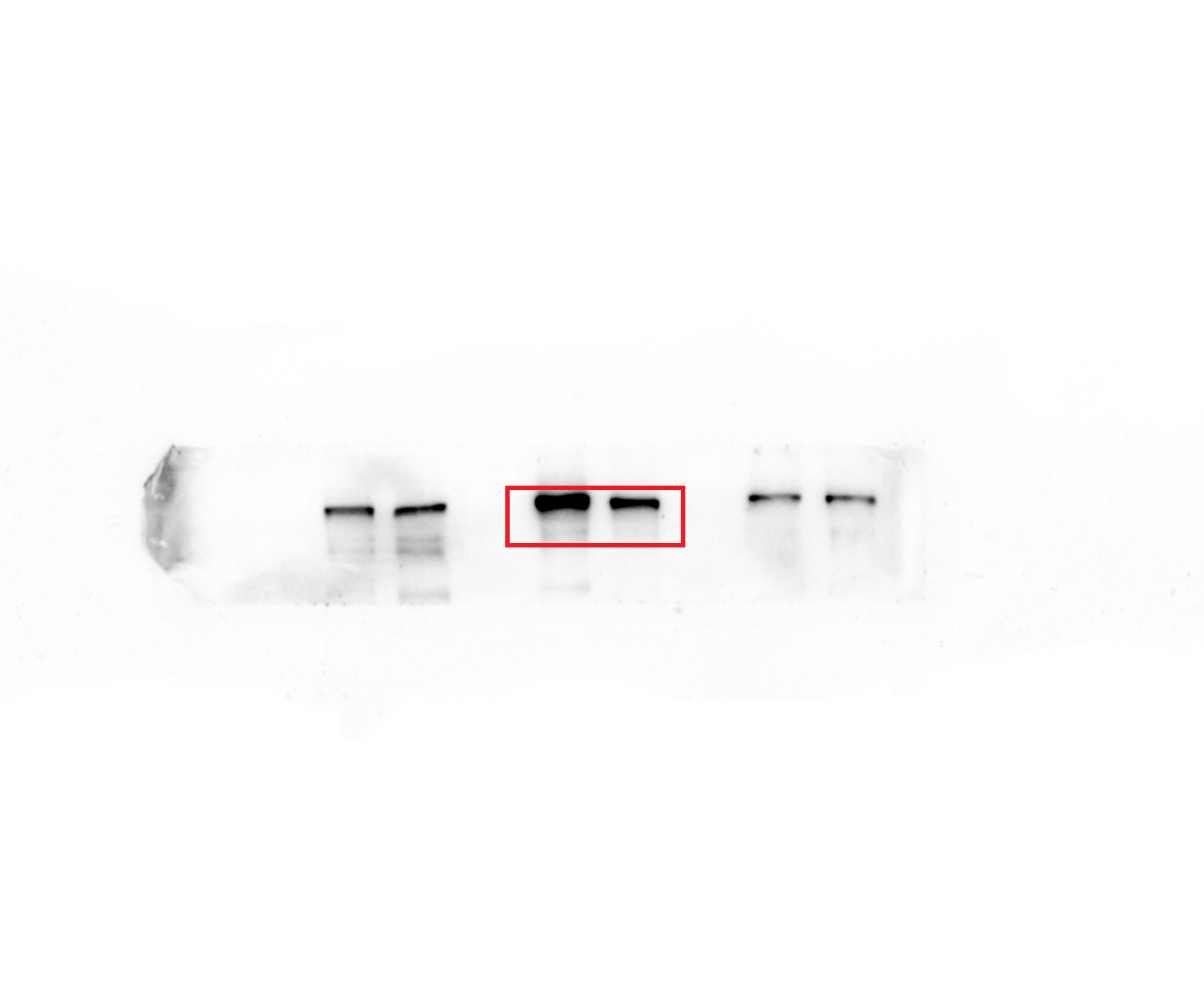

Supplement: Supplemental Information 2 [file peerj-11-15700-s002.zip › raw data 2-western blot/Original Image for Fig 9A/N-cadherin.Tif]

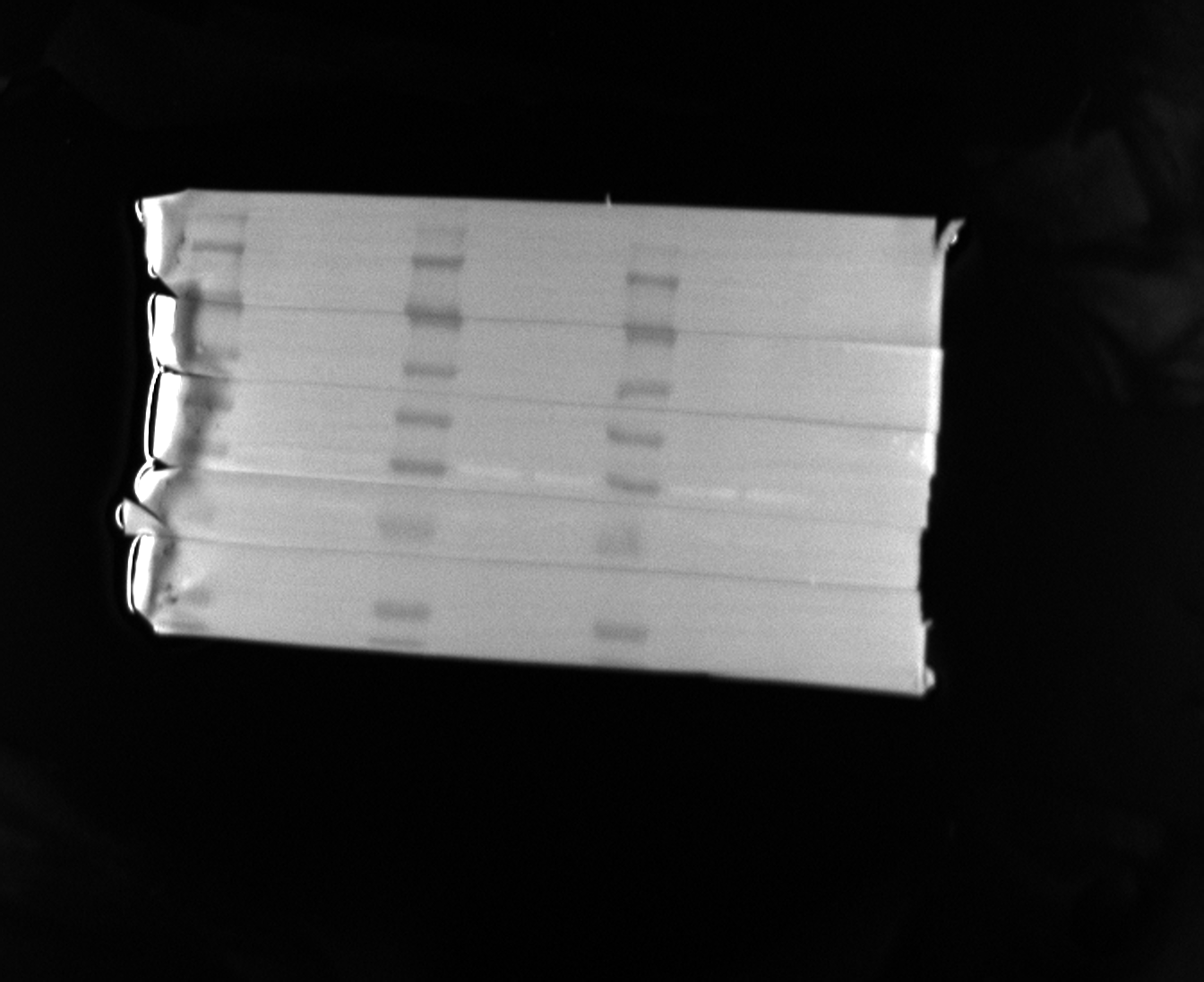

Supplement: Supplemental Information 2 [file peerj-11-15700-s002.zip › raw data 2-western blot/Original Image for Fig 9A/Snai1 and GAPDH intact membrane .Tif]

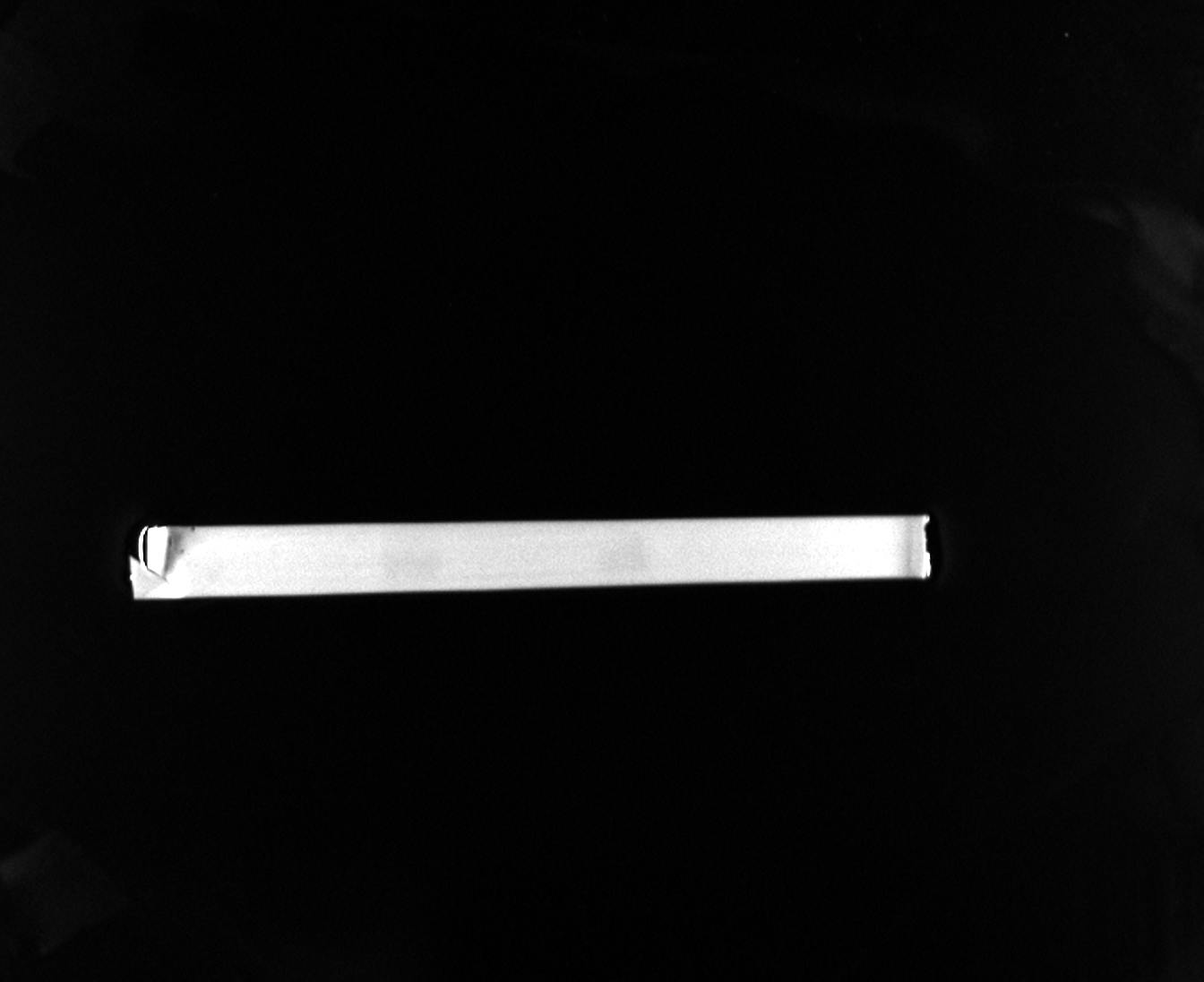

Supplement: Supplemental Information 2 [file peerj-11-15700-s002.zip › raw data 2-western blot/Original Image for Fig 9A/Snai1 original drawing.Tif]

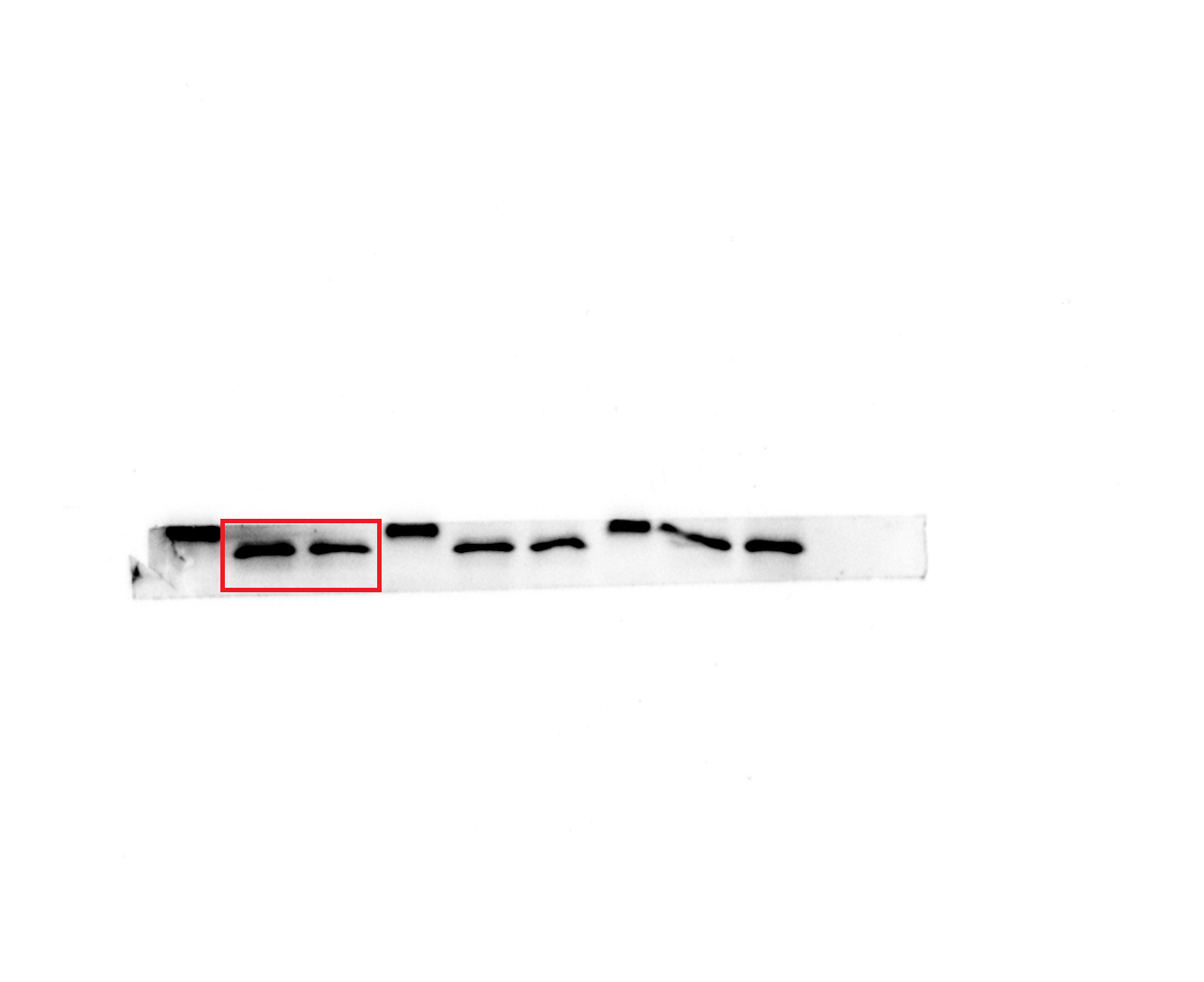

Supplement: Supplemental Information 2 [file peerj-11-15700-s002.zip › raw data 2-western blot/Original Image for Fig 9A/Snai1.Tif]

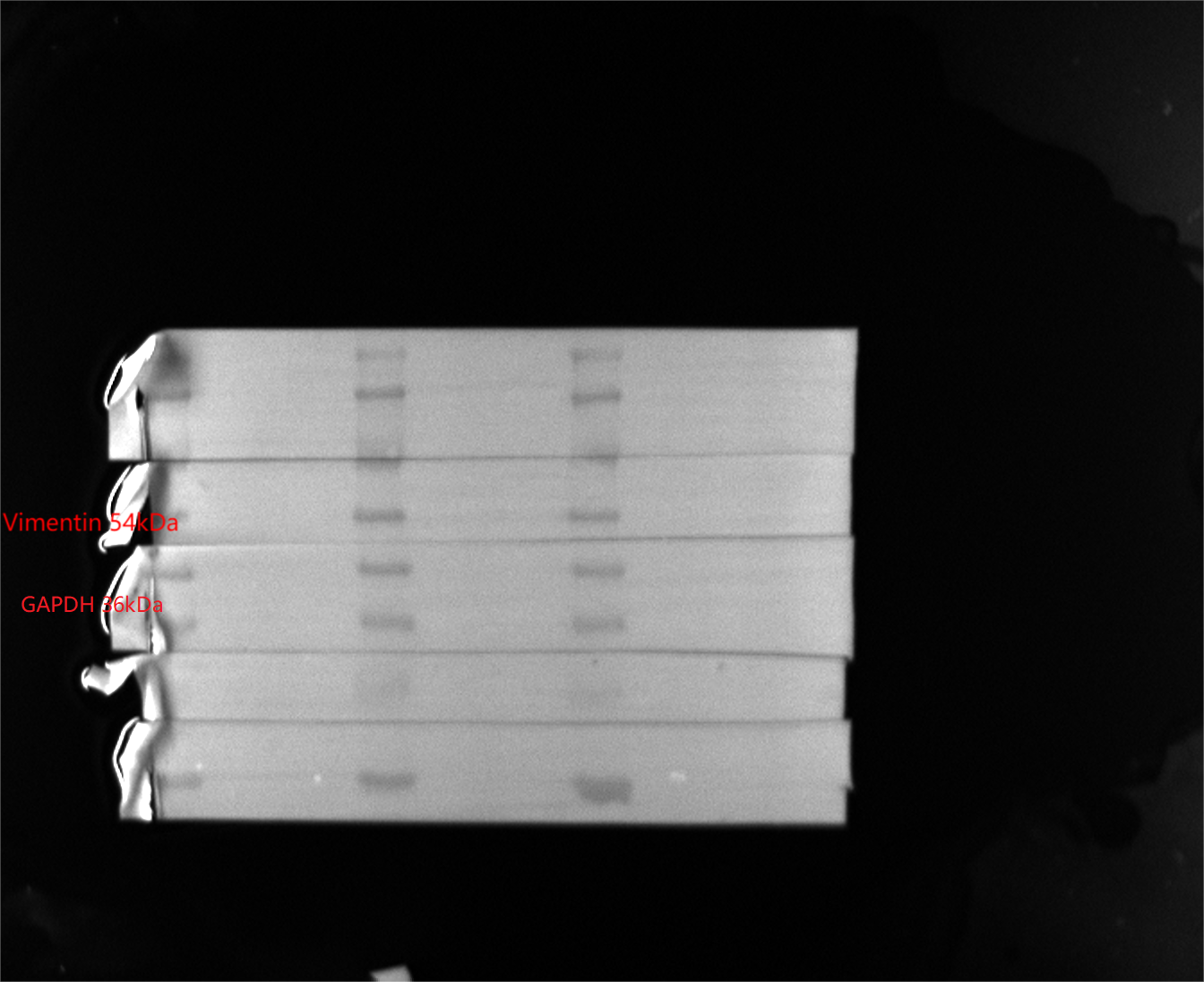

Supplement: Supplemental Information 2 [file peerj-11-15700-s002.zip › raw data 2-western blot/Original Image for Fig 9A/Vimentin and GAPDH intact membrane .png]

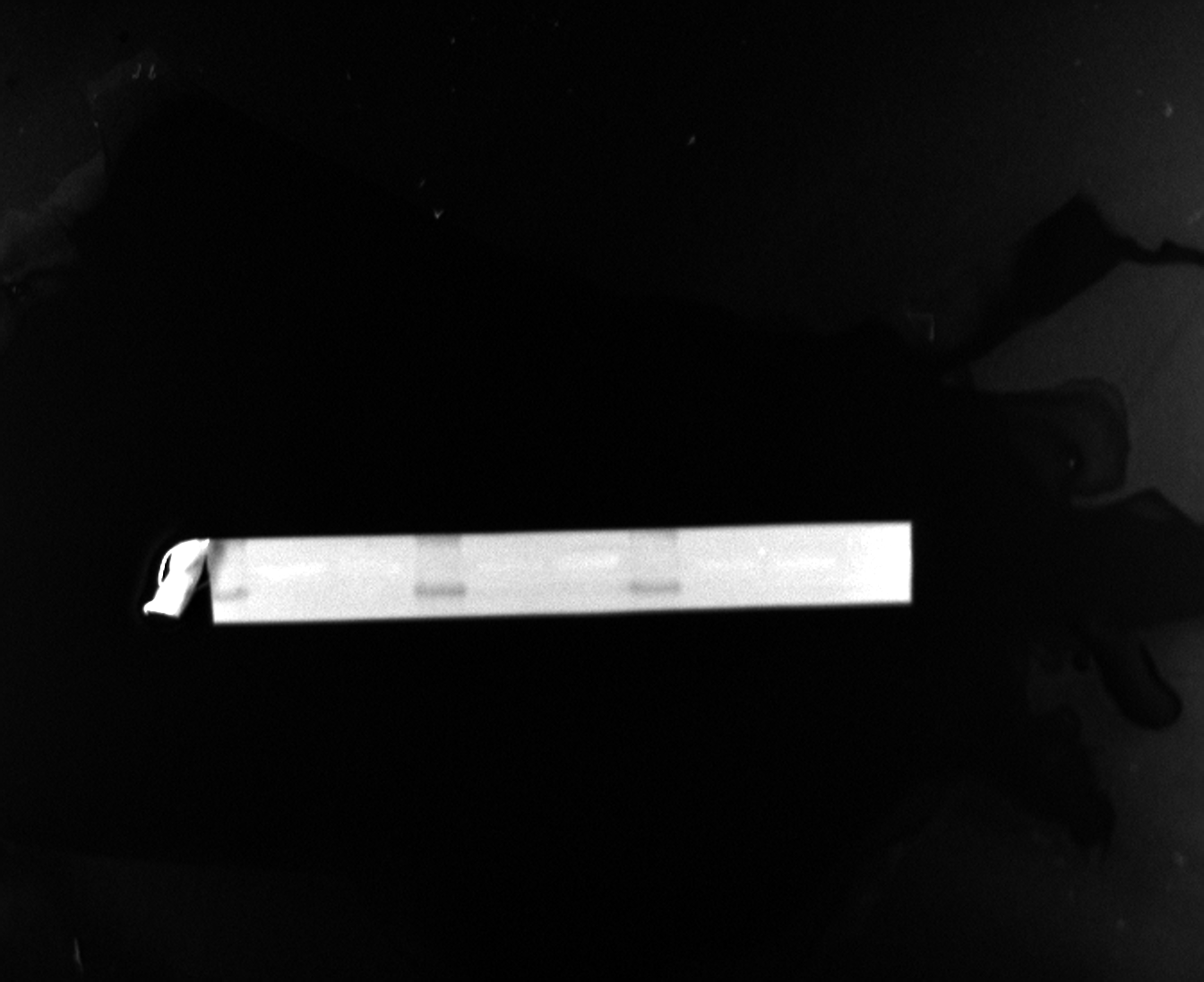

Supplement: Supplemental Information 2 [file peerj-11-15700-s002.zip › raw data 2-western blot/Original Image for Fig 9A/Vimentin original drawing.Tif]

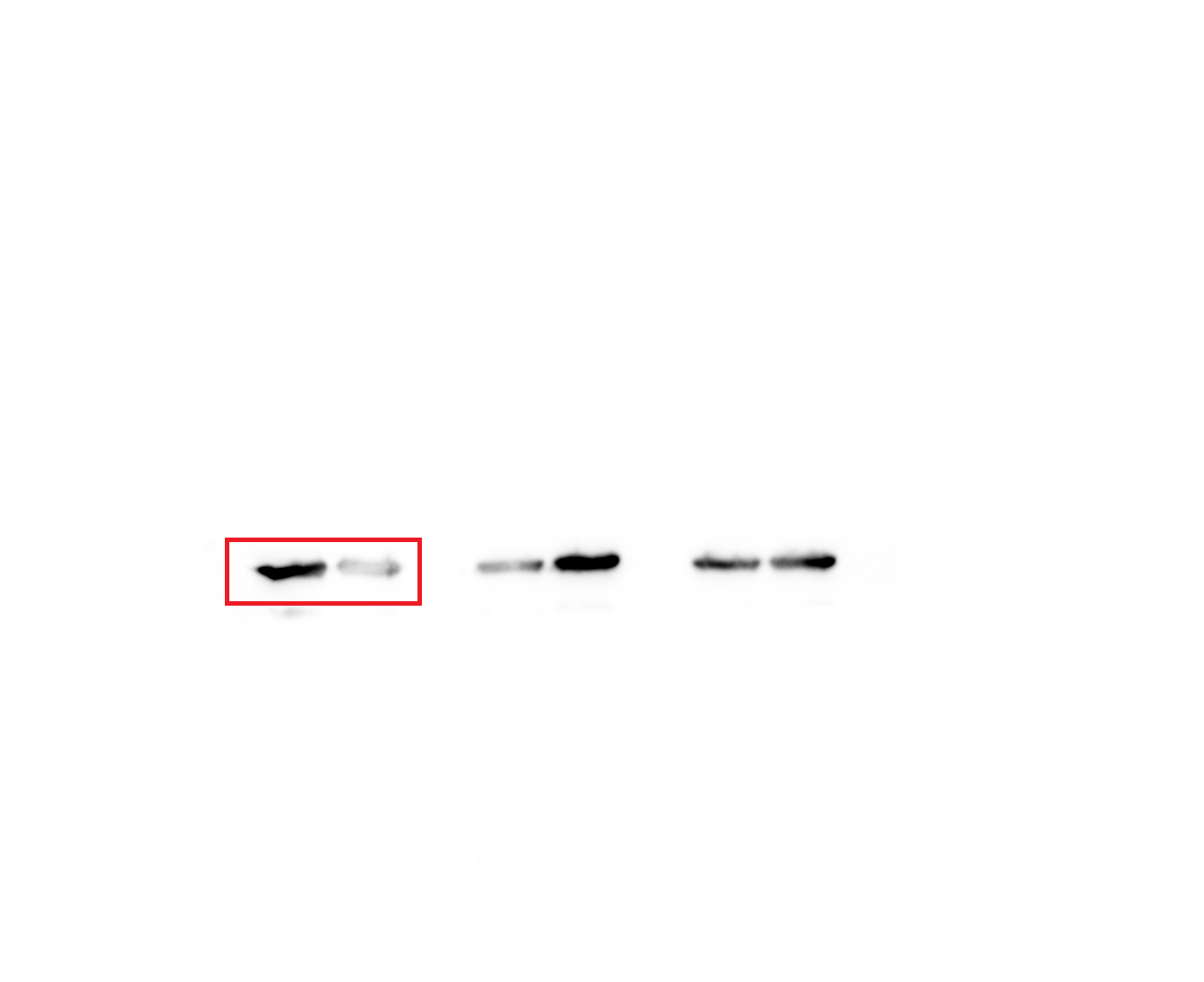

Supplement: Supplemental Information 2 [file peerj-11-15700-s002.zip › raw data 2-western blot/Original Image for Fig 9A/Vimentin.Tif]

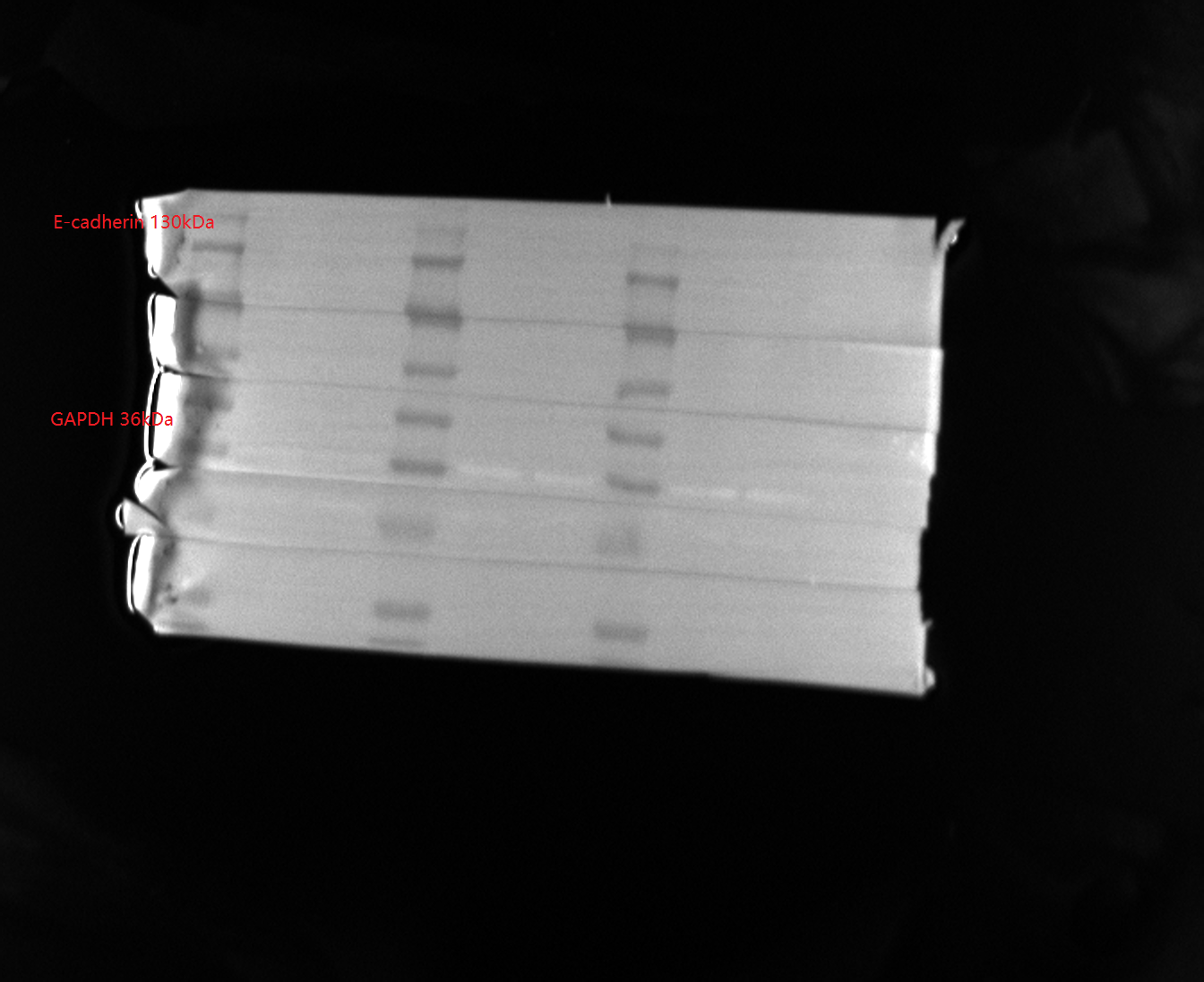

Supplement: Supplemental Information 2 [file peerj-11-15700-s002.zip › raw data 2-western blot/Original Image for Fig 9B/E-cadherin and GAPDH intact membrane.Tif]

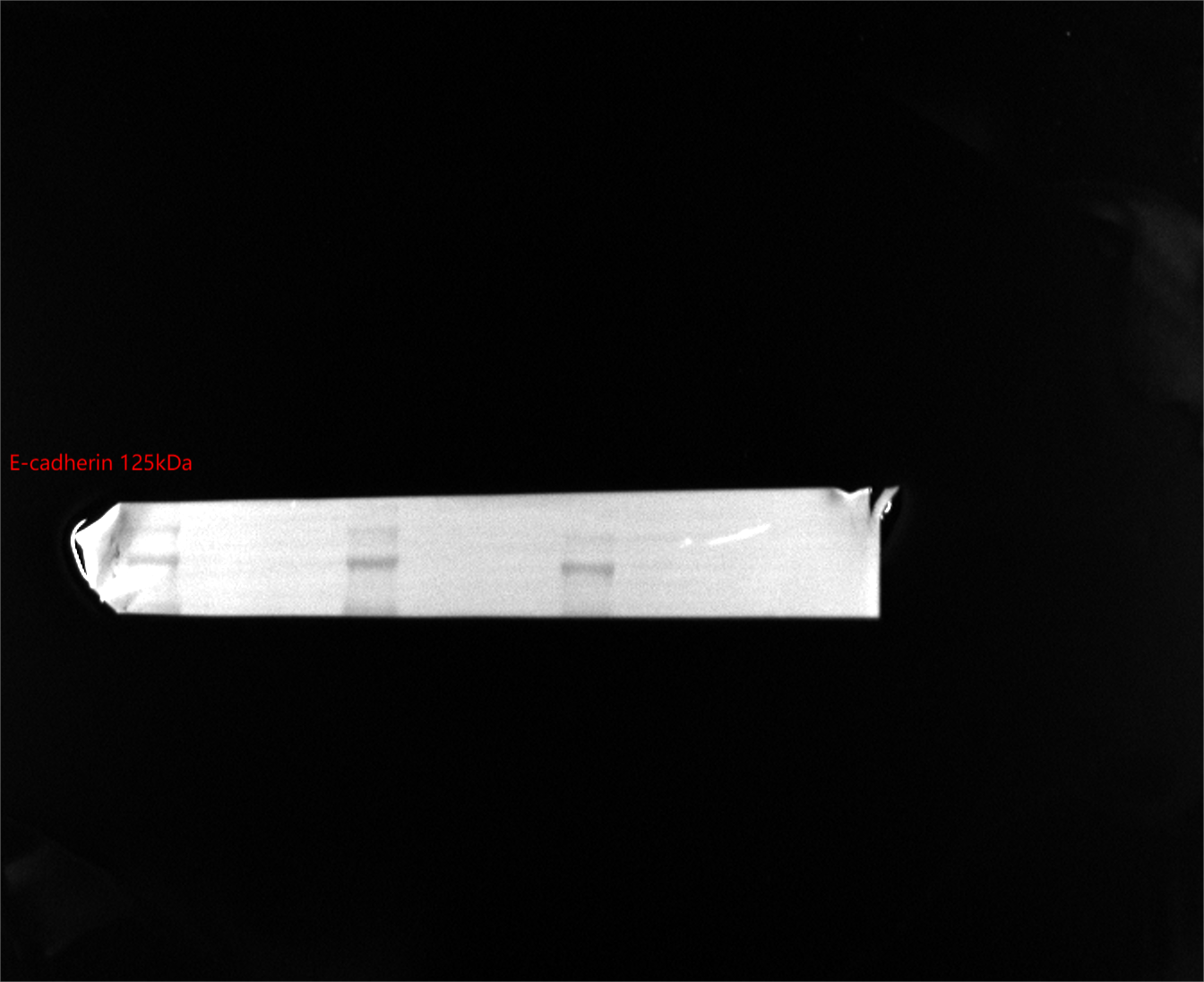

Supplement: Supplemental Information 2 [file peerj-11-15700-s002.zip › raw data 2-western blot/Original Image for Fig 9B/E-cadherin-original drawing.png]

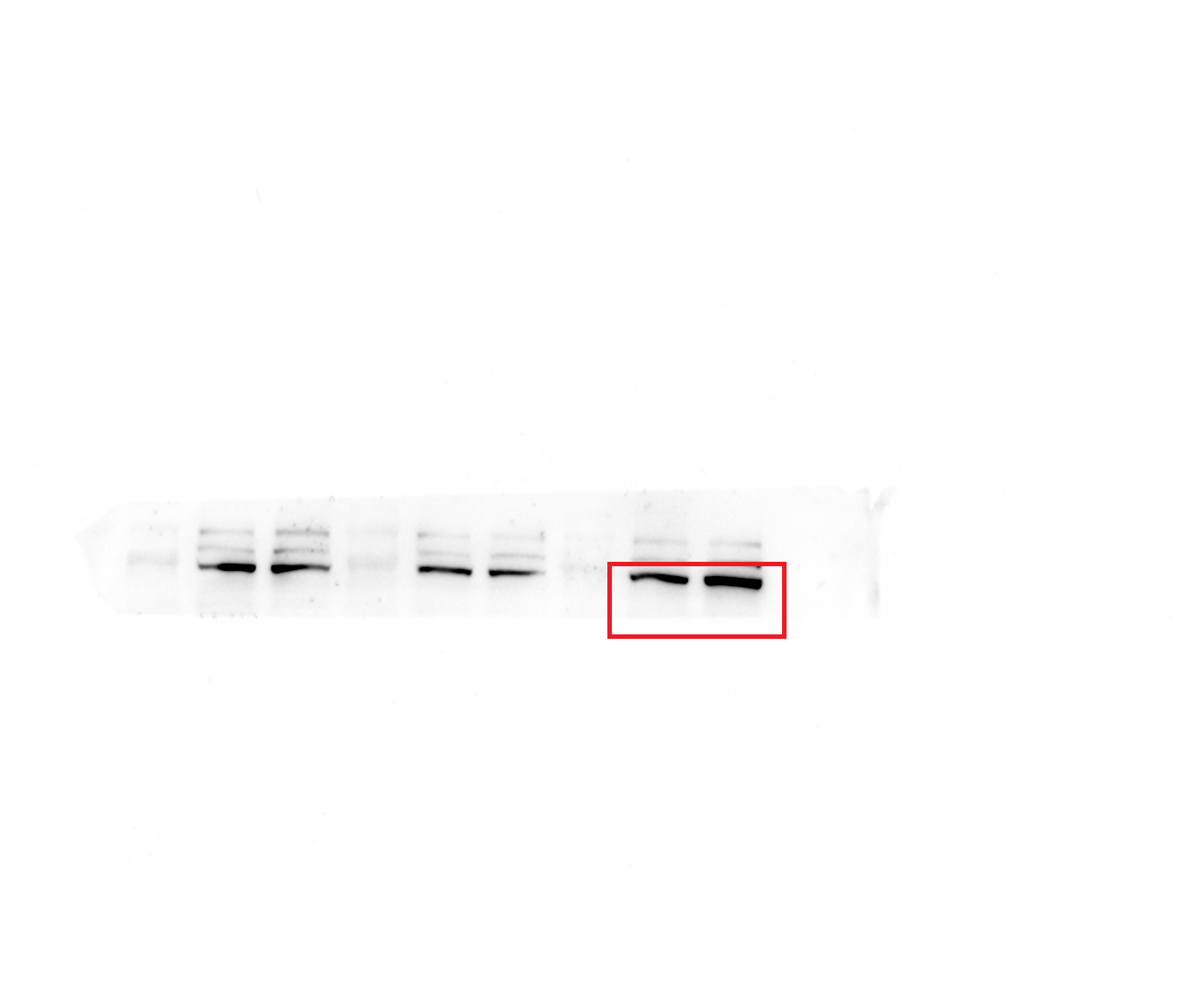

Supplement: Supplemental Information 2 [file peerj-11-15700-s002.zip › raw data 2-western blot/Original Image for Fig 9B/E-cadherin.Tif]

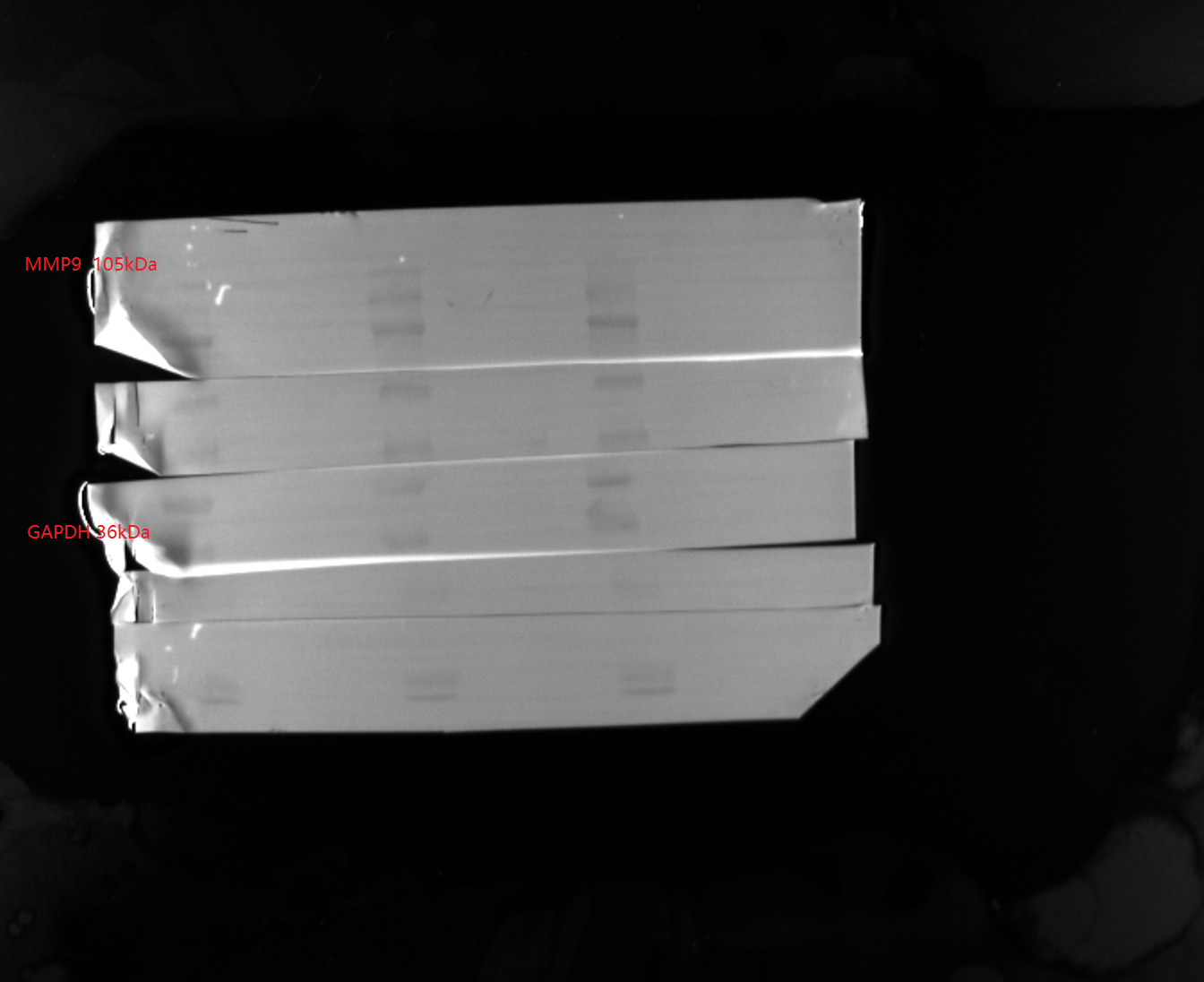

Supplement: Supplemental Information 2 [file peerj-11-15700-s002.zip › raw data 2-western blot/Original Image for Fig 9B/MMP9 and GAPDH intact membrane.Tif]

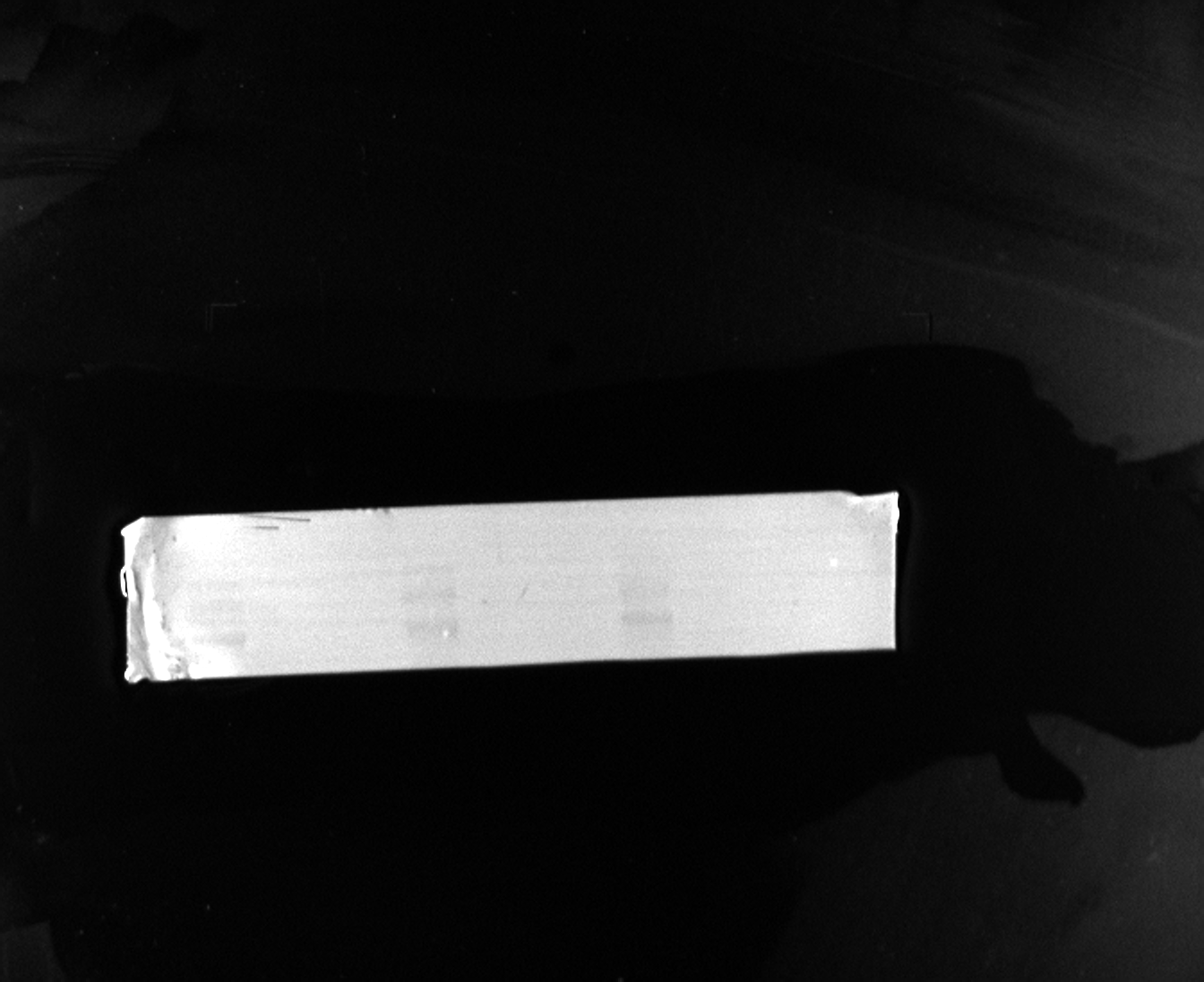

Supplement: Supplemental Information 2 [file peerj-11-15700-s002.zip › raw data 2-western blot/Original Image for Fig 9B/MMP9 original drawing.Tif]

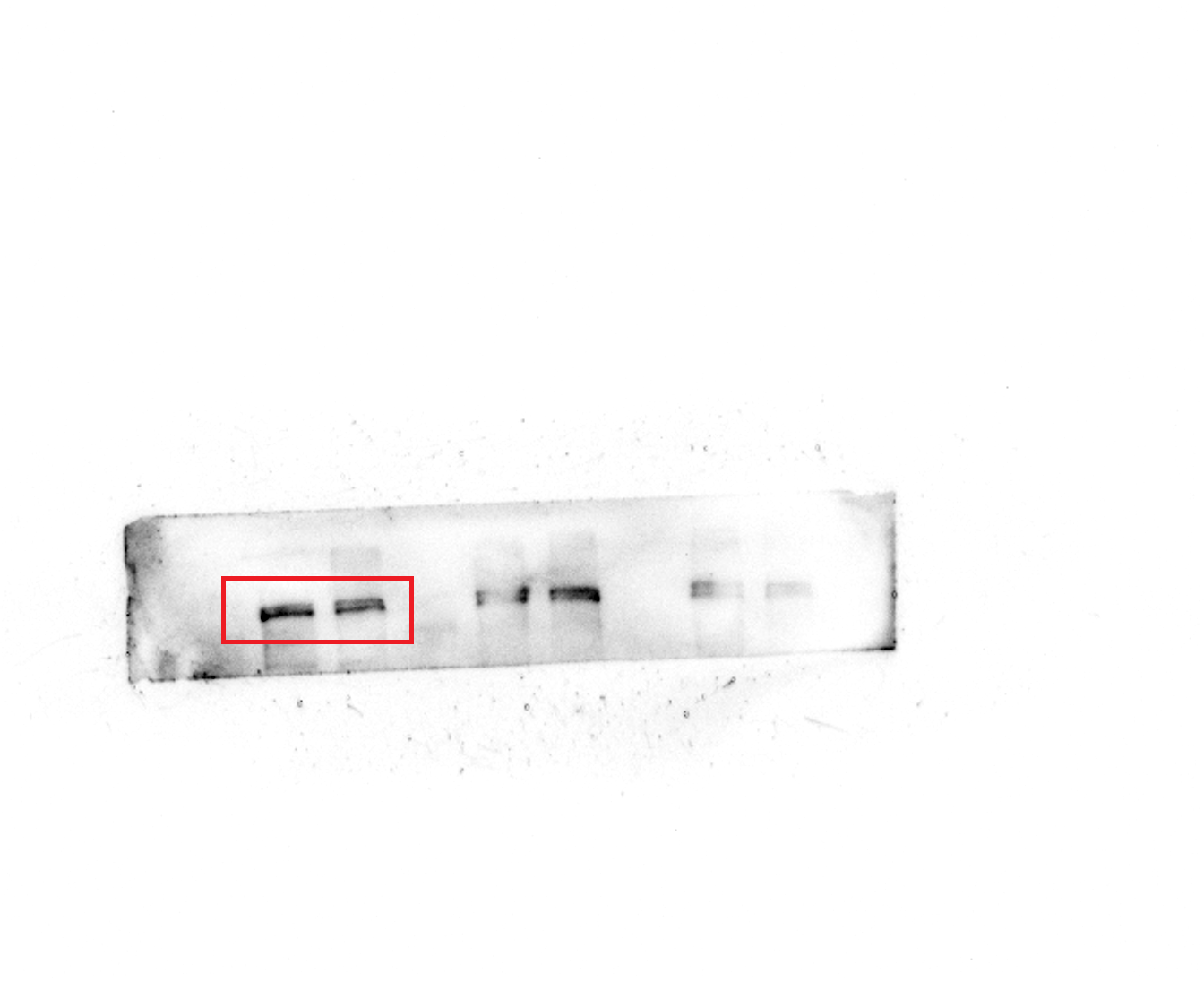

Supplement: Supplemental Information 2 [file peerj-11-15700-s002.zip › raw data 2-western blot/Original Image for Fig 9B/MMP9.Tif]

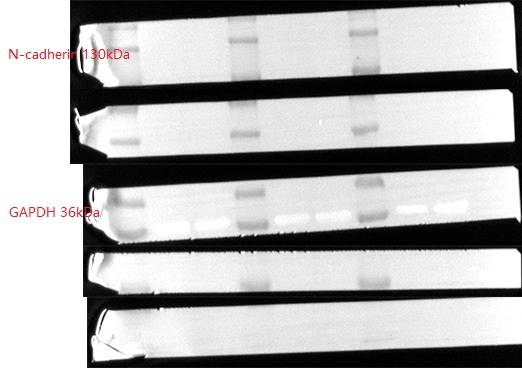

Supplement: Supplemental Information 2 [file peerj-11-15700-s002.zip › raw data 2-western blot/Original Image for Fig 9B/N-cadherin and GAPDH intact membrane.jpg]

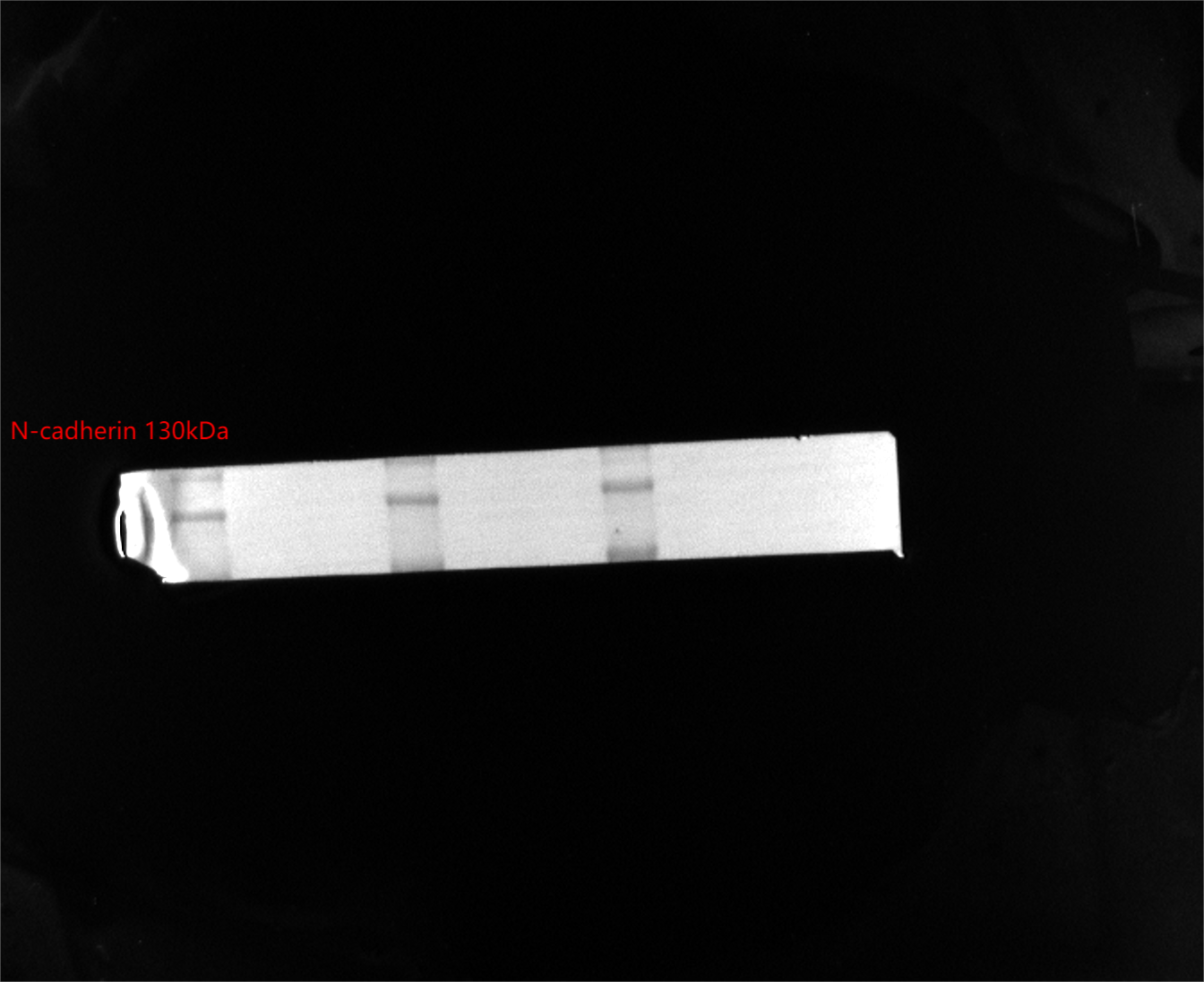

Supplement: Supplemental Information 2 [file peerj-11-15700-s002.zip › raw data 2-western blot/Original Image for Fig 9B/N-cadherin-original drawing.png]

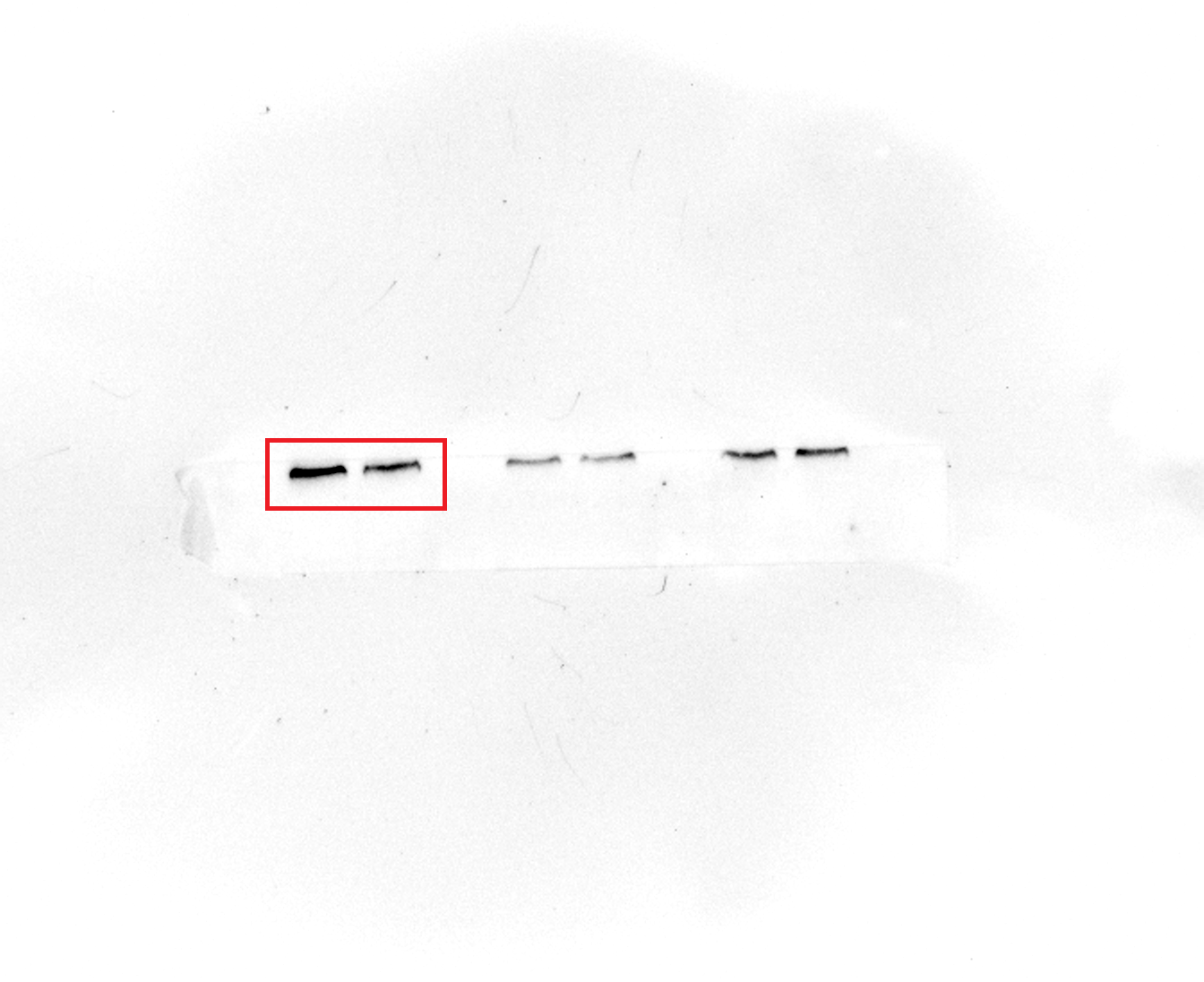

Supplement: Supplemental Information 2 [file peerj-11-15700-s002.zip › raw data 2-western blot/Original Image for Fig 9B/N-cadherin.Tif]

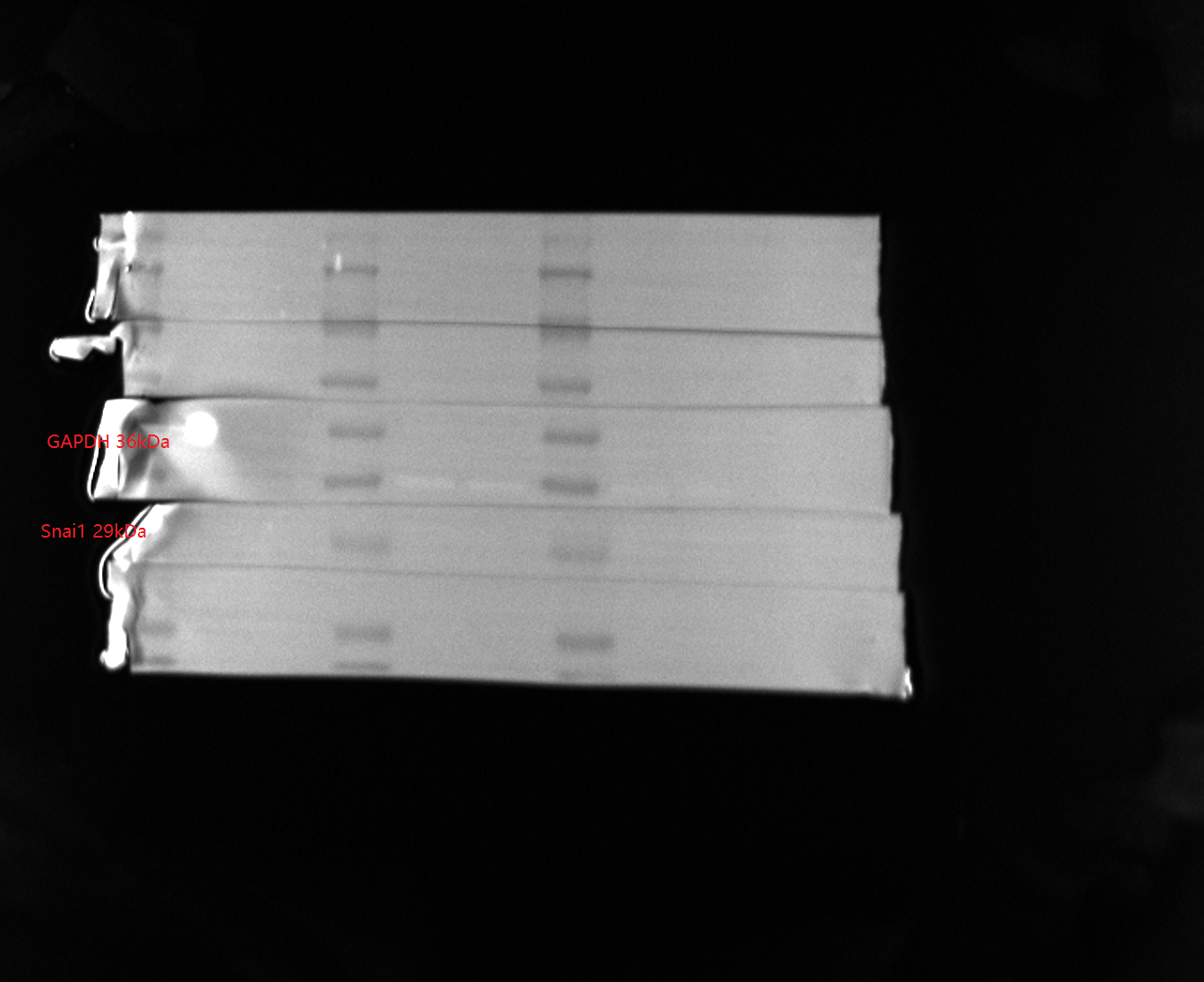

Supplement: Supplemental Information 2 [file peerj-11-15700-s002.zip › raw data 2-western blot/Original Image for Fig 9B/Snai1 and GAPDH intact membrane.Tif]

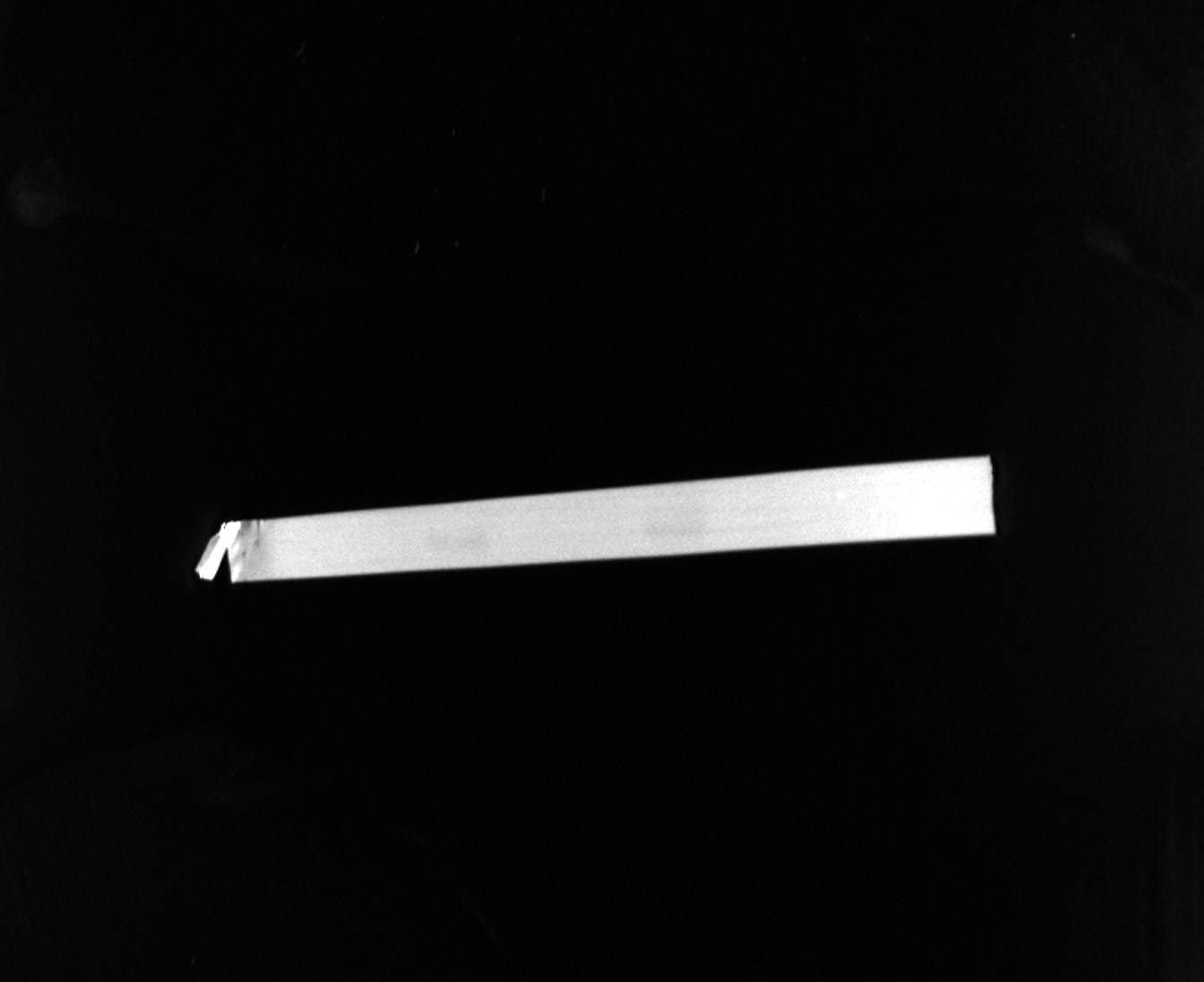

Supplement: Supplemental Information 2 [file peerj-11-15700-s002.zip › raw data 2-western blot/Original Image for Fig 9B/Snai1-original drawing.Tif]

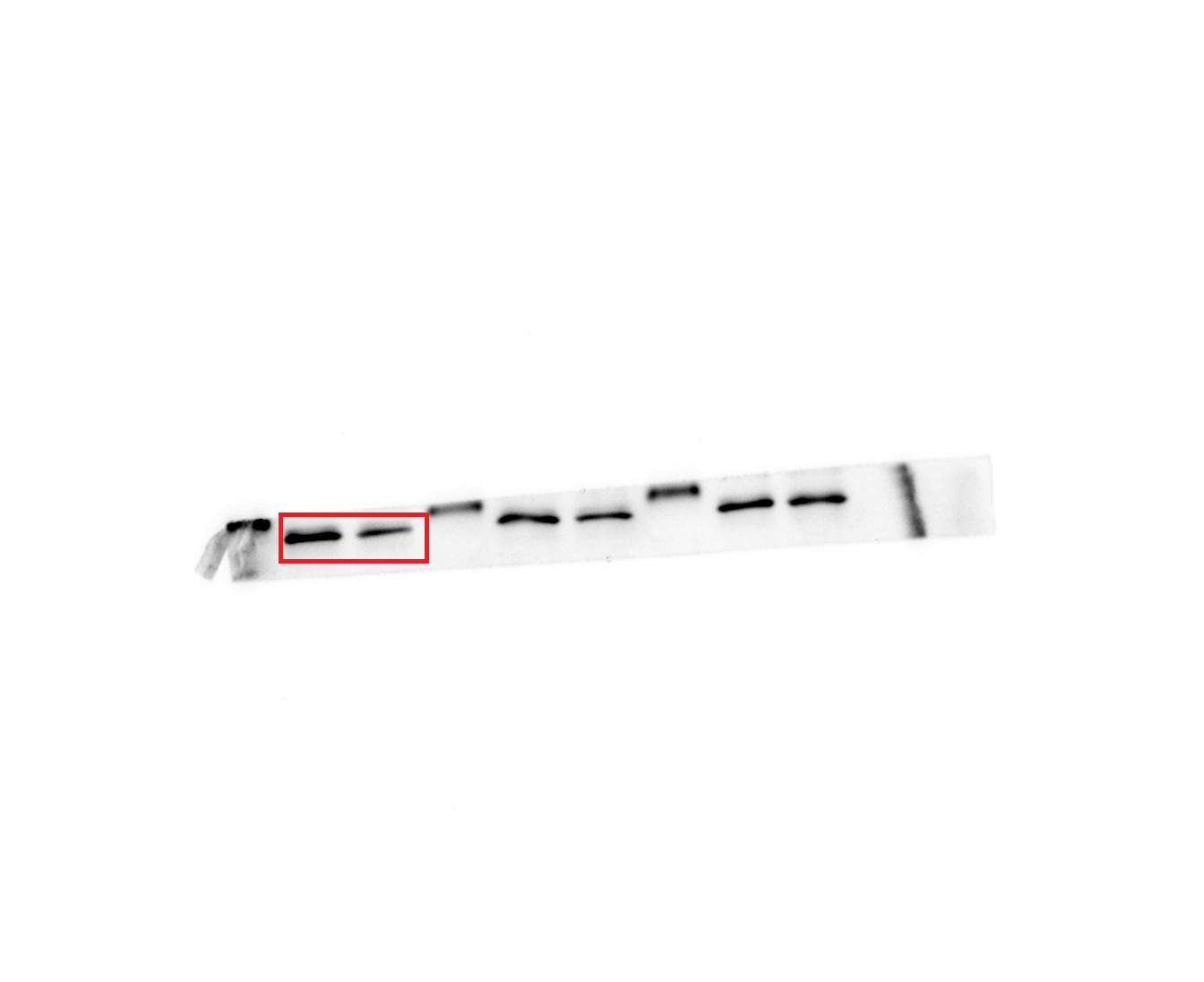

Supplement: Supplemental Information 2 [file peerj-11-15700-s002.zip › raw data 2-western blot/Original Image for Fig 9B/Snai1.Tif]

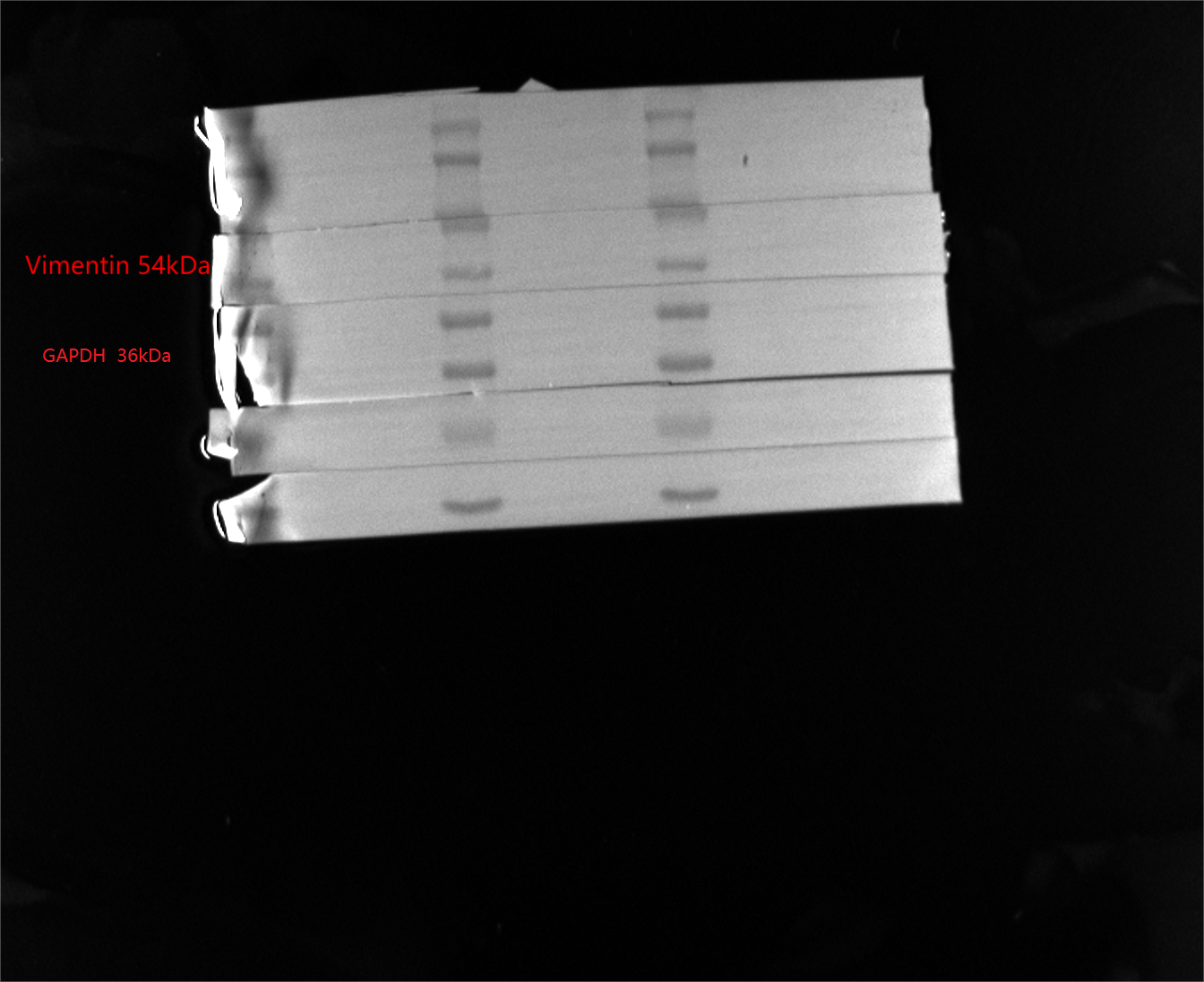

Supplement: Supplemental Information 2 [file peerj-11-15700-s002.zip › raw data 2-western blot/Original Image for Fig 9B/Vimentin and GAPDH intact membrane.png]

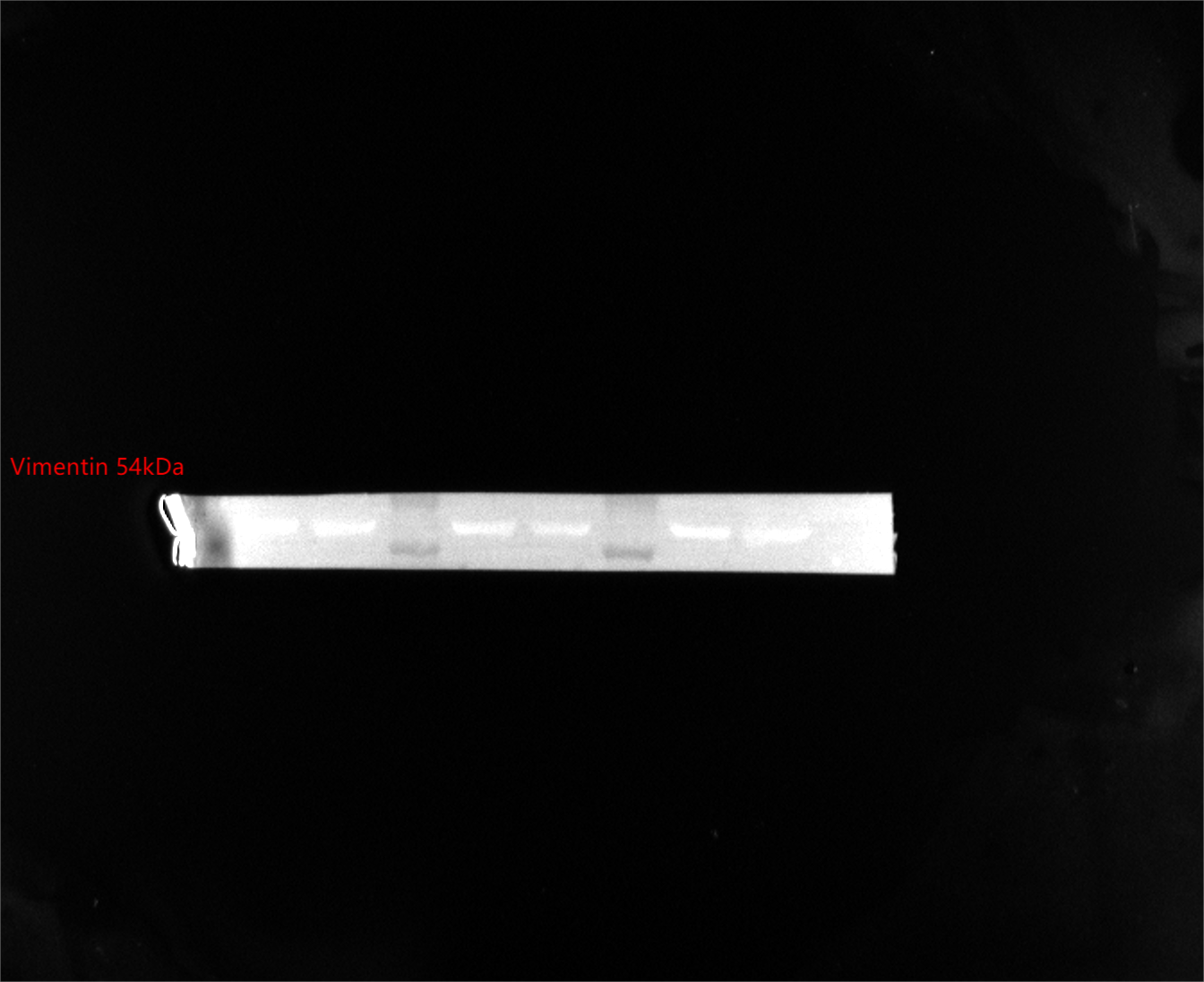

Supplement: Supplemental Information 2 [file peerj-11-15700-s002.zip › raw data 2-western blot/Original Image for Fig 9B/Vimentin-original drawing.png]

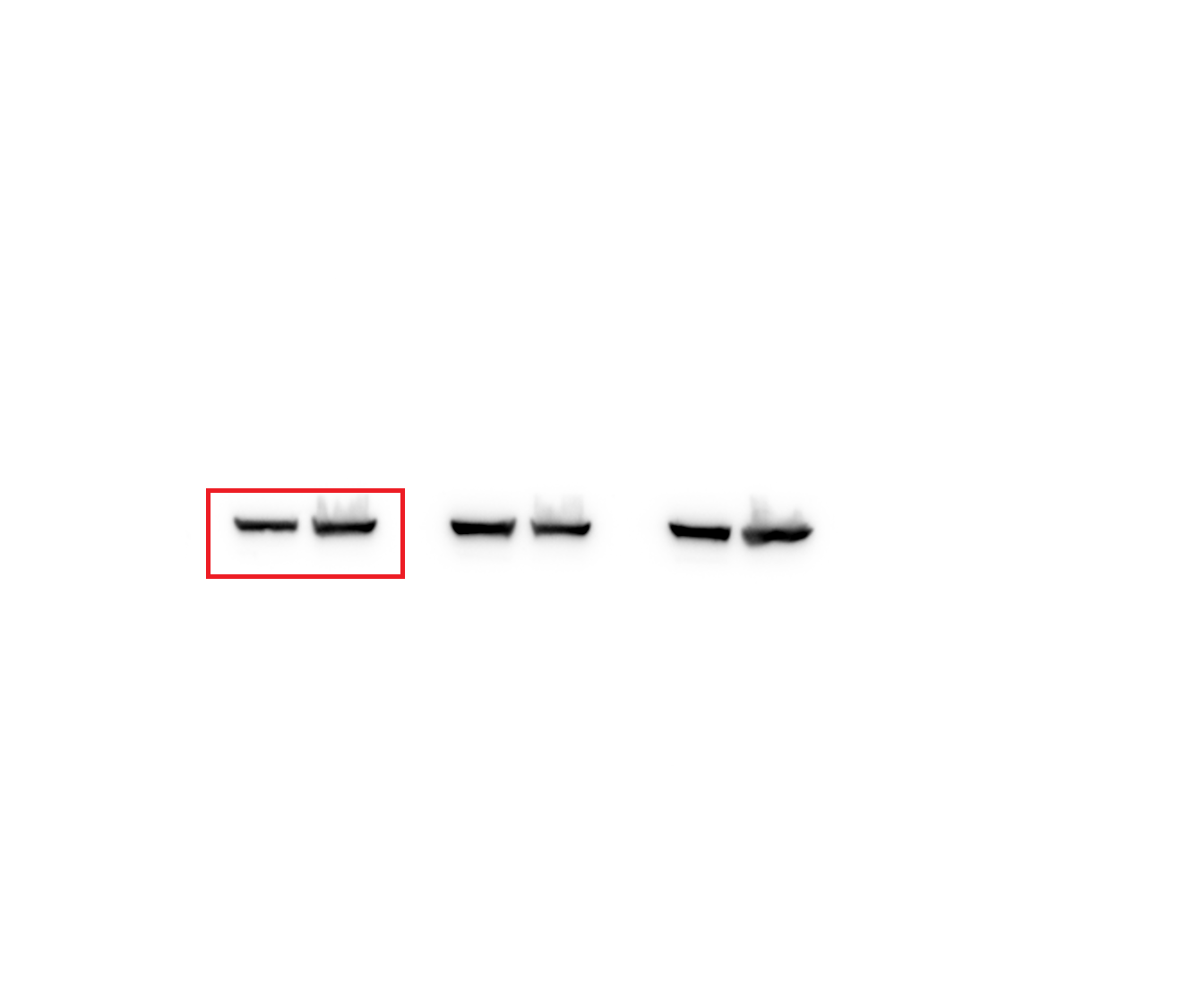

Supplement: Supplemental Information 2 [file peerj-11-15700-s002.zip › raw data 2-western blot/Original Image for Fig 9B/Vimmentin.Tif]
